# Evidence for Transcript Networks Composed of Chimeric RNAs in Human Cells

**DOI:** 10.1371/journal.pone.0028213

**Published:** 2012-01-04

**Authors:** Sarah Djebali, Julien Lagarde, Philipp Kapranov, Vincent Lacroix, Christelle Borel, Jonathan M. Mudge, Cédric Howald, Sylvain Foissac, Catherine Ucla, Jacqueline Chrast, Paolo Ribeca, David Martin, Ryan R. Murray, Xinping Yang, Lila Ghamsari, Chenwei Lin, Ian Bell, Erica Dumais, Jorg Drenkow, Michael L. Tress, Josep Lluís Gelpí, Modesto Orozco, Alfonso Valencia, Nynke L. van Berkum, Bryan R. Lajoie, Marc Vidal, John Stamatoyannopoulos, Philippe Batut, Alex Dobin, Jennifer Harrow, Tim Hubbard, Job Dekker, Adam Frankish, Kourosh Salehi-Ashtiani, Alexandre Reymond, Stylianos E. Antonarakis, Roderic Guigó, Thomas R. Gingeras

**Affiliations:** 1 Bioinformatics and Genomics, Centre for Genomic Regulation and Universitat Pompeu Fabra, Barcelona, Catalonia, Spain; 2 Affymetrix Inc., Santa Clara, California, United States of America; 3 Department of Genetic Medicine and Development, University of Geneva Medical School, University Hospitals of Geneva, Geneva, Switzerland; 4 Wellcome Trust Sanger Institute, Hinxton, Cambridgeshire, United Kingdom; 5 The Center for Integrative Genomics, University of Lausanne, Lausanne, Switzerland; 6 Center for Cancer Systems Biology and Department of Cancer Biology, Dana-Farber Cancer Institute, and Department of Genetics, Harvard Medical School, Boston, Massachusetts, United States of America; 7 Cold Spring Harbor Laboratory, Cold Spring Harbor, New York, United States of America; 8 Structural Biology and Biocomputing Programme, Spanish National Cancer Research Centre, Madrid, Spain; 9 Institute of Research in Biomedicine and Barcelona Supercomputer Center, Joint Program on Computational Biology. Parc Científic de Barcelona, Universitat de Barcelona, Facultat de Biologia, Barcelona, Catalonia, Spain; 10 Department of Biochemistry and Molecular Pharmacology, University of Massachusetts Medical School, Worcester, Massachusetts, United States of America; 11 University of Washington, Seattle, Washington, United States of America; 12 New York University Abu Dhabi, Abu Dhabi, United Arab Emirates, and Center for Genomics and Systems Biology, Department of Biology, New York University, New York, New York, United States of America; 13 Departament de Ciènces Experimentals i de la Salut, Universitat Pompeu Fabra, Barcelona, Catalonia, Spain; The John Curtin School of Medical Research, Australia

## Abstract

The classic organization of a gene structure has followed the Jacob and Monod bacterial gene model proposed more than 50 years ago. Since then, empirical determinations of the complexity of the transcriptomes found in yeast to human has blurred the definition and physical boundaries of genes. Using multiple analysis approaches we have characterized individual gene boundaries mapping on human chromosomes 21 and 22. Analyses of the locations of the 5′ and 3′ transcriptional termini of 492 protein coding genes revealed that for 85% of these genes the boundaries extend beyond the current annotated termini, most often connecting with exons of transcripts from other well annotated genes. The biological and evolutionary importance of these chimeric transcripts is underscored by (1) the non-random interconnections of genes involved, (2) the greater phylogenetic depth of the genes involved in many chimeric interactions, (3) the coordination of the expression of connected genes and (4) the close *in vivo* and three dimensional proximity of the genomic regions being transcribed and contributing to parts of the chimeric RNAs. The non-random nature of the connection of the genes involved suggest that chimeric transcripts should not be studied in isolation, but together, as an RNA network.

## Introduction

The complex repertoire of RNAs found in cells from yeast to human is unexpected and at times seemingly daunting. In part, this complexity is composed of transcripts whose sequences are chimeras formed from sequences found in separate genes. The origins of such chimeric RNAs are derived from multiple biological mechanisms as well as technical artifacts. The biological sources of chimeric RNAs have been seen to stem from both DNA and RNA mediated events. DNA mediated event include such mechanisms as chromosomal rearrangements, gene duplications, retrotransposition and retrotransduction [1]. Each of these mechanisms provide for the construction of novel chimeric transcriptional units that are composed of sequences that are distally separated within a genome of a cell type. Reciprocally, the detection of chimeric RNAs has recently proven to provide an informative means of identifying potentially novel structural variations (SV) in genomes [2]. In several specific cases, the detection of chimeric transcripts has been shown to be unrelated to the presence of SV such as the formation of the JAZF1-JJAZ1 found in normal endometrial stromma cells and made from 5′ exons of transcripts from the *JAZF1* gene on chromosome 7p15 and the 3′ exons of *JJAZ1* (also known as *SUZ12*) located on chromosome 17q11. In this case no SV could be detected [3]. This RNA has been observed to be translated into a chimeric anti-apoptotic protein. This and other identified chimeric transcripts expand the coding potential encoded in genomes by joining together non-contiguous and non-linear regions of genomes [4]. The apparent absence of genomic SVs and the highly specific and reproducible occurrence of the junction sites joining the chimeric sequences suggest a RNA-mediated mechanism for the creation of these transcripts [3], [5], [6], [7], [8], [9], [10], [11].

Complicating the identification of chimeric RNAs is the clear possibility of technical artifacts caused by the template switching capabilities of reverse trancriptase (RT) in both *in vitro* [12] and *in vivo* [13] conditions. First observed in the replication of retroviruses [14], [15], this property was suggested to be the basis for a copy choice mechanism of recombination in retroviruses and has been seen to be operational during *in vitro* experiments leading to RT-mediated chimeric products (RT). With the development of high throughput RNA sequencing (RNAseq) methods that are reliant on RT for conversion of RNA into double stranded cDNAs, the template switching activity of RT has been observed in these sequencing methods by two Drosophila species mixture experiments [16]. These observations prompt a careful verification of any observed chimeric transcripts.

The focus of the studies presented here was to systematically analyze the diversity of transcripts found within and extending from the annotated boundaries of genic loci in the human genome. These studies follow in the footsteps of a set of earlier works performed with a limited number of human genes that were located in the 1% of the human genome selected by the Encyclopedia of DNA Elements (ENCODE) project [5], [17]. In these earlier studies two main observations were reported. First was the pervasive transcription across the analyzed 1% of the genome (93% coverage) and the second was that many of the genic loci were connected to other genic loci resulting in the formation of chimeric transcripts. In these current studies we extended our analyses to genes on chromosomes 21 and 22 and sought to determine if chimeric transcripts can be detected among the genes analyzed, if their detection originates because of technical artifacts and to determine if evidence can be collected that supports the biological importance of any detected chimeric transcripts.

## Results

### Discovery of novel chimeric transcripts through RACE reactions and tiling arrays

**Figure 1:**
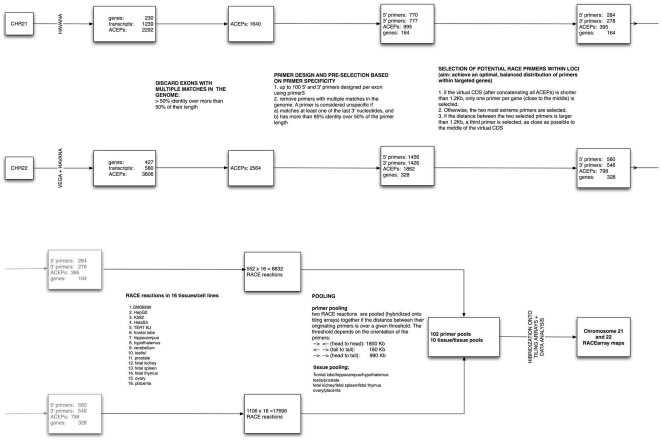
RACEarray experiment flowchart. The successive steps of the RACEarray experiments are represented as a flowchart for both chromosomes 21 and 22. It should be read from left to right and from top to bottom.

Protein coding genes encoded on human chromosomes 21 and 22 were interrogated using a combination of methods including rapid amplification of cDNA ends (RACE) and tiling arrays [6], and deep RNA sequencing (RNAseq). Figure 1 describes the overall experimental design used for the RACE and tiling array experiments. In summary, we first selected 1,193 exons from 492 annotated gene loci present on chromosomes 21 and 22 for which we could select highly specific 5′ and 3′ RACE primers. We designed 844 5′-RACE and 824 3′-RACE primers, and carried out the corresponding RACE reactions using polyadenylated (poly A+) selected RNA isolated from 11 normal human tissues and five transformed cell lines In total, 26,688 RACE reactions were performed (see Material and Methods).

The products of the RACE reactions were then analyzed using chromosome 21 and 22 tiling arrays interrogating the non-repeat portions of these chromosomes at 17 nucleotide resolution. RACE reactions were pooled prior to hybridization to the tiling arrays. First, RACE reactions for a given primer originating from different tissues were pooled together (pairing: prostate and testis; ovary and placenta; brain frontal lobe, brain hippocampus and brain hypothalamus; and fetal kidney, fetal spleen and fetal thymus, Figure 1). Second, within a given sample or mixture, RACE reactions originating from different primers were pooled together. A pooling strategy was designed to maximize the genomic distance between primers pooled together, while minimizing the number of pools required (see Materials and Methods). The minimum genomic distance between RACE primers was set to 1.8 Mb when primers were in head to head orientation, 180 Kb when they were in tail to tail orientation, and 990 Kb when they were in head to tail orientation. A total of 102 primer pools were required (see Figure S1 for the actual distribution of genomic distances between primers). Each one of the primer pool-tissue pool combinations was hybridized separately into a tiling array. A total of 1,020 array hybridizations were conducted.

Intensities from the arrays were processed using specially developed software to determine continuous sites of transcription (Materials and Methods). This software discarded unspecific probes (that is, array probes that appear more than once in the genome, 7% in total) and builds sites of transcription with parameters optimized to mirror the features of known exons (Figures S2 and S3). These sites of transcription are referred to as RACEfrags. In total, 306,368 RACEfrags were initially obtained. To eliminate RACEfrags coming from unspecific priming and/or unspecific cross-hybridization, an *in silico* RACEarray simulator was used ([6], Materials and Methods, and Figure S4). Furthermore, to eliminate products originating in very highly abundant transcripts not targeted by our RACE reactions, negative controls were utilized in which the RNA samples or mixtures were hybridized into the arrays without RACE amplification (Materials and Methods). In total, 262,472 RACEfrags remained after applying these filters.

RACEfrags typically correspond to detection of exon expression of the interrogated genes or to unspliced transcripts. The pooling of primers however does challenge the correct assignment of RACEfrags to the originating primer. To meet this challenge a heuristic method was developed that assigns RACEfrags to the corresponding primers. The heuristic attempts to satisfy several constraints: genomic proximity (in compatible orientation) to the primer, consistency of connectivity across multiple samples, and support of connectivity by multiple primers (see Materials and Methods and Figure S5). After this analysis a total of 195,982 RACEfrags were assigned to the original RACE primers

**Figure 2:**
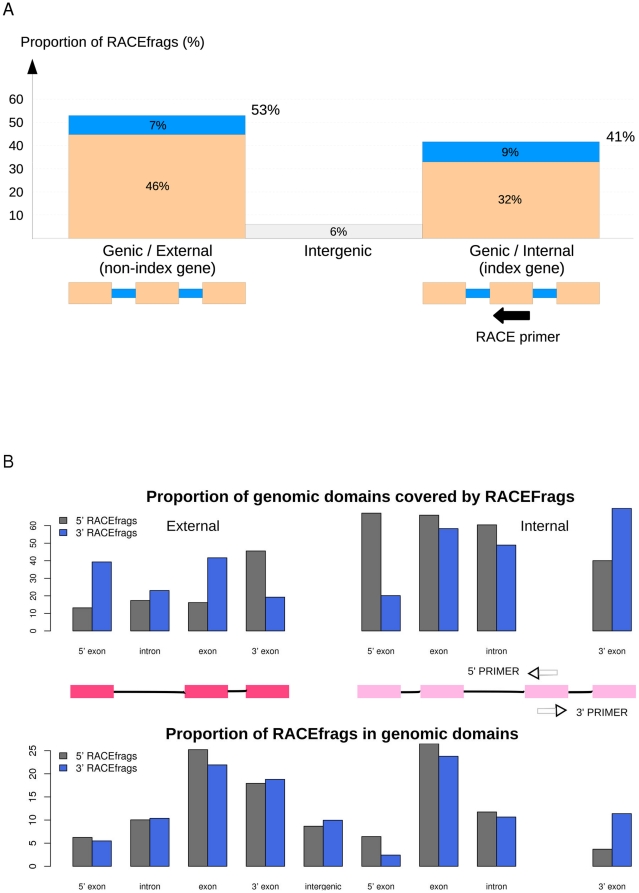
RACEfrag transcription map statistics. **A- Distribution of RACEfrags among annotated genomic domains.** The proportion of RACEfrags overlapping different annotated genic features is represented in this histogram. Blue: intronic RACEfrags; Light orange: exonic RACEfrags; Light grey: intergenic RACEfrags. The three categories on the X axis are, from left to right: (1) - external genic RACEfrags (i.e. RACEfrags falling within the boundaries of a gene not interrogated by RACE, (2) - intergenic RACEfrags, (3) - internal RACEfrags (i.e., RACEfrags detected within the RACE-primed gene). **B- RACEfrag descriptive analysis.** The top bar plot represents proportions of genomic domains covered by RACEfrags, and the bottom bar plot represents proportions of RACEfrags in different genomic domains (refinement of part A). As RACE is carried out in the two possible directions, 5′ and 3′, each bar plot is thus sub-divided into two sub-bar plots: proportions relative to 5′ RACEfrags in gray, and proportions relative to 3′ RACEfrags in blue. As expected: (1) RACEfrag coming from a given gene covers this gene more than any other gene; (2) for a given RACE-interrogated gene, internal exons and introns are equally covered by 5′ and 3′ RACEfrags, whereas 5′ most exons are more covered by 5′ RACEfrags and 3′ most exons by 3′ RACEfrags. The bottom bar plot also shows that most RACEfrags are exonic, then intronic and finally intergenic, and that exonic RACEfrags are first found in internal exons, then in most 3′ exons and finally in most 5′ exons.

The identified and assigned RACEfrags displayed a length ranging from 47 to 4,493 base pairs, and a median length of 133 base pairs. As expected this is similar to the median length of exons (128 nt) in the Gencode [18] annotated human transcriptome. The proportion of annotated exons covered by at least one RACEfrag is 85.7%. While internal exons and introns of a gene are equally covered by 5′ and 3′ RACEfrags assigned to this gene, most 5′ exons/3′ exons are covered by 5′ RACEfrags/3′ RACEfrags respectively, as we expected (Figure 2B). We evaluate the sensitivity of the RACEarray map in terms of number of annotated exon connections that could be detected given the primer configuration (called detectable exon connections), and that are indeed detected by the (RACEfrag, primer) pairs. Of the total 19,578 exon connections that could be detected, 15,296 were, representing a sensitivity of about 78%.

The total 195,982 RACEfrags assigned to primers across all tissues and mixtures corresponded to 79,560 distinct loci of transcription. When overlapping RACEfrags found in different biological samples were melded, a total of 17,450 projected RACEfrags, covering 5.1 Mb in chromosomes 21 and 22 (15%), resulted. The distributions of detected RACEfrags both within the gene from which the RACE reaction started (index gene) and the genomic domains outside of the index gene are given in Figure 2A. Although there is a majority of exonic and genic RACEfrags (78% and 94% respectively) once projected onto the genome, RACEfrags represent an increase of 11,394 over the set of exons previously annotated (65% of all projected RACEfrags), and of 2.9 Mb over the bases previously known to reside in transcripts in chromosome 21 and 22.

### Incidence of observations of chimeric RNAs

**Figure 3:**
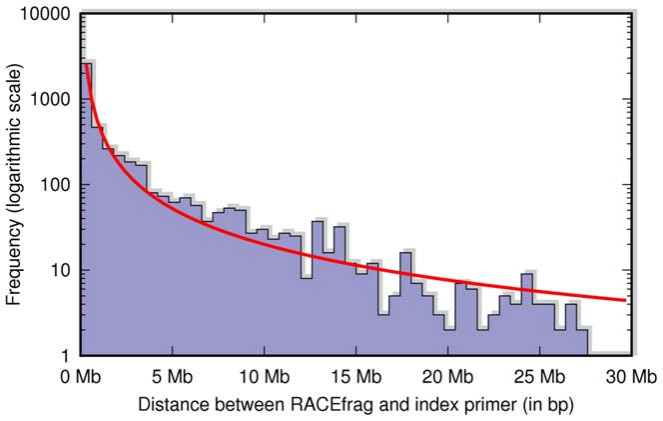
Distribution of genomic distances between RACEfrags and their respective index RACE primers. The raw histogram is shown in purple, the corresponding curve fit in red [25].

**Figure 4:**
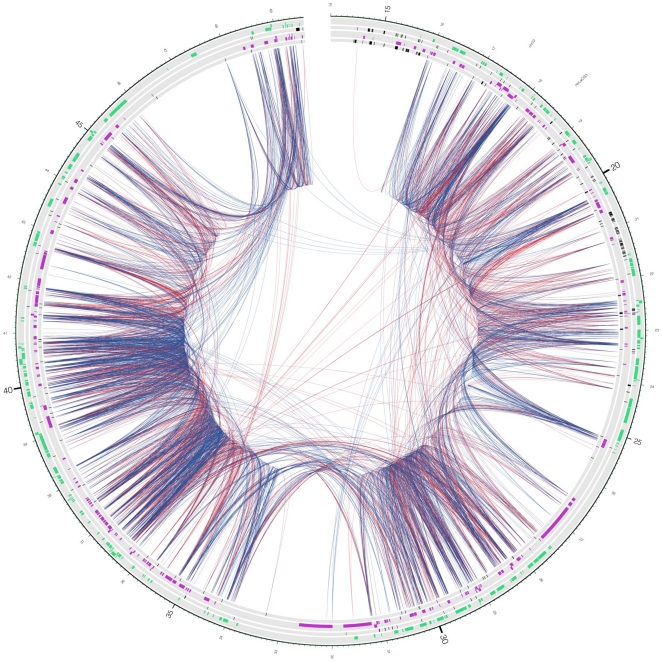
Transcriptional network on chromosome 22 in a pool of testis and prostate tissues. The chromosome is depicted as a circle [45], and RACEfrag connections as inner links between genomic regions (5′ and 3′ RACE connections are red and blue, respectively). The circular tracks are, going inwards: (1) - chromosome scale (in megabases, starting at 14 Mb), (2) - plus-strand annotated genes (green), (3) - plus-strand annotated pseudogenes (black), (4) - minus-strand annotated genes (purple), (5) - minus-strand annotated pseudogenes (black).

Interestingly, more than half of the gene anchored RACEfrags (59%) map outside of the annotated boundaries of the index genes (Figure 2A). The median distance between the index primer and the RACEfrags assigned to the primer is 321,600 nucleotides (nts). Genomic distances as large as 34 Mb (index gene: ICOSLG on chromosome 21 and a RACEfrag in cell line K562) were observed. The frequency distribution of distances between index exon and assigned RACEfrags can be fitted well to a power law, with exponent varying across different tissues, but generally in the range between -1.4 and -1.6 (Figure 3). A circular representation map [19] of chromosome 22 that depicts the gene locations that are connected using 5′ or 3′ RACE analysis on a pool of RNA obtained from testis and prostate tissues is depicted in Figure 4 (for RACEfrag maps obtained for chromosomes 21 and 22 using RNAs from other sources, see Material S1 section 5a and Figures S6 and S7). It is notable that 72% of all RACEfrags mapping outside of index genes map to exons of other genes, supporting a non-random characteristics of the observed chimeric RNAs.

**Figure 5:**
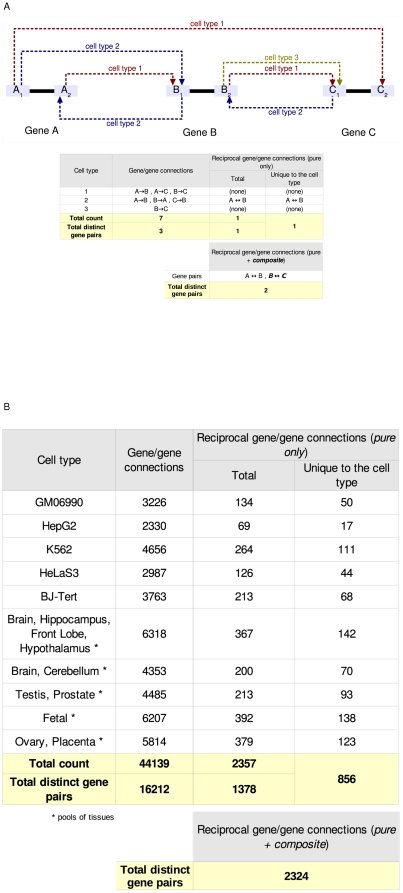
Reciprocal gene/gene connections. **A - General definition of reciprocal gene/gene connections**. Top panel: graphical illustration of reciprocity. Exons are symbolized by light blue boxes, introns by solid black lines. Dashed arrows, directed from the index exon to the RACEfrag, correspond to chimeric connections in distinct cell types, which are rendered in different colors. Two reciprocal gene/gene connections can be observed in this example, between genes A and B, and B and C. The (A–B) reciprocal pair is said to be (i), *unique to cell type 2*, and (ii), *pure* (*i.e.*, its reciprocity is observed at least once in the same condition, cell type *2* in this example), whereas (B–C) is *composite* (*i.e.*, its reciprocity can only be deduced from connections observed in different cell types). The counts of each connection type in this example are summarized in the tables in the bottom panel. **B - Observed numbers of reciprocal gene/gene connections across 10 different cell types**. This table is based on the template used in part A.

Because of the systematic 5′ and 3′ RACE interrogations of the same exons within a gene, we often observe genes that are reciprocally connected through the RACE reactions. Specifically, we define this condition in the following way: two genes A and B are reciprocally connected if and only if, a RACE primer in A originates a RACEfrag within locus boundaries of B, and a RACE primer in B originates a RACEfrag in A (Figure 5A: e.g. exon2 of gene A with exon1 of gene B). Thus, a reciprocal set of RACE results independently confirm each other and provide a high confidence set of RACEfrags and RACEfrag to primer connections. The total number of gene to gene reciprocal connections for each biological sample tested is summarized in Figure 5B (illustrated in Figure 5A), and detailed in Table S1. A total of 2,324 reciprocal connections (Figure S8) were observed (corresponding to 14% of the total gene to gene connections), of which 37% are cell type specific (Figure 5B). This number of reciprocal gene-gene connections is between 2- and 3-fold larger than the number expected given the underlying primer to RACEfrag connections (see Material S1 section 5b). The reciprocal gene to gene connections across all conditions are displayed for both chromosomes in Figures S9 and S10 respectively. Approximately 50% of these chimeric connections were observed to be originating from loci annotated on different genomic strands.

### Characterization and validation of chimeric transcripts

#### RT-PCR, cloning and sequencing

**Figure 6:**
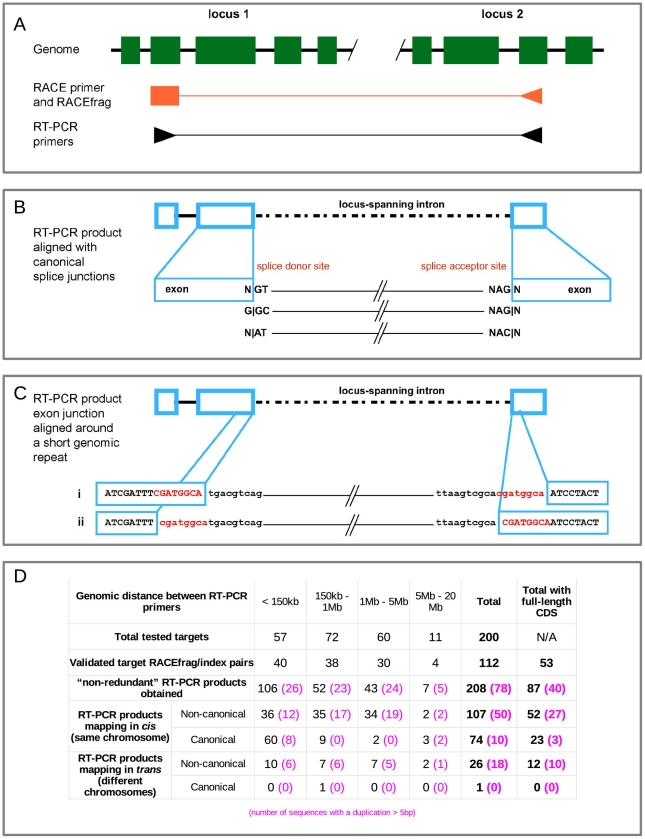
Experimental results from RT-PCR cloning and sequencing of chimeric connections. **A- Experimental design**. The presence of a chimeric transcript between loci 1 and 2 was tested using a pair of nested primers, depicted as two black arrow heads at the bottom, targeting the index exon on one side, and the detected RACEfrag on the other side (both in orange). **B- Annotation of novel locus-joining canonical splice sites in RT-PCR products**. The three types of canonical exon junctions considered are enumerated at the bottom. **C- Example of short genomic duplication around locus-joining exon junctions**. Two alignments of such a junction are possible, as depicted in the bottom part of the figure. Intronic and exonic sequences are represented in lowercase and uppercase letters, respectively. Note that the duplicated sequence (in red letters) is present only once in the RT-PCR product. **D- Results of the mapping of RT-PCR products to the genome.** Detailed results for each genomic distance bin are reported. Statistics for RT-PCR sequences affected by the type of short duplications illustrated in Figure 6C are noted in pink, between parentheses. Results of the analysis of the coding potential in each category are presented in the rightmost column. RT-PCR products mapping “*in trans*” are products that include sequences from chromosomes other than the chromosome that contained the index gene and the connected RACEfrag.

The array detected chimeric transcripts were first characterized and validated by molecular cloning and sequencing. Starting from the RNA pools from which they were detected, a total of 200 RACEfrags were selected for RT-PCR amplification, full-length cloning and sequencing. These RACEfrags were comprised of 67 reciprocal gene to gene connections and 133 additional chimeric cases stratified according to increasing distances from the index gene to the RACEfrag (Figure 6A–C). A total of 112 chimeric connections (56%) were confirmed by sequencing, corresponding to 208 distinct transcript sequences (Figure 6D). These sequences were then analyzed by the HAVANA manual annotation pipeline (Material and Methods) and two features of note were noticed. First, while the majority of the transcripts sequences corresponding to relatively short extensions (less than 150 Kb) mapped to the genome with introns exhibiting canonical splicing sequences at the junction sites forming the chimeric regions (57%), the trend was the opposite for extensions beyond this distance. Of these more distal connections 85% of the sequences exhibited non-canonical splicing sequences at the junction sites. (Figure 6D). Second, a proportion of the non-canonical junction regions (51%) contain small genomic duplications (between 5 bp and 10 bp) at the genomic sequences flanking the junction sites of the chimeric RNAs. The duplicated sequence appears only once within the sequence of the RT-PCR product (see Figure 6C). Interestingly, the presence of similar length short repeats have been reported previously at the junction sites of chimeric RNAs found in fly, mouse and human [8]. A larger proportion of duplicated sequences is observed in annotated non-canonical introns (21%) compared to canonical ones (10%). Among these chimeric transcripts were the 27 that included sequences from chromosomes other than the chromosome containing the index gene. (Figure 6D). Thus, a genome-wide analysis for chimeric RNAs may reveal substantial extensive gene to gene connections occurring among all chromosomes and indicating similar functionally related genes involved in this fashion.

#### RACEarray RNA chimeras are reproducible across RNA samples

While many gene to gene connections observed in this study are specific to a given cell line/tissue type, a majority can also be detected in more than one sample. Indeed, 9,647 gene to gene connections were detected in two or more samples (57.9%), 6,264 in three or more (37.6%) and 169 (1.0%) in all of the samples analyzed. Further the pairwise correlation involving reciprocal gene to gene connections observed between cell types was determined. These correlations range from 0.05 to 0.35, and were all found to be statistically significant (P-values<2.2*10^−16^, Materials S1 section 5c and Figure S11).

#### Same chimeric RNA transcripts found in tiling array and RNAseq experiments

Results observed in this analysis were compared to those recently published using deep sequencing of RNA (RNAseq) ([20] and the Human Body Map 2.0 data generated on Illumina HiSeq 2000). First, comparison of the tiling array data of total polyA+RNA from K562 cells with the results of PET ditag sequencing [20] of cytosolic polyA+RNA from the same cell line. A total of 4,656 gene to gene connections were detected by RACE-array compared to 412 detected by PET ditag sequencing. Out of these, a total of 56 (p<0.0001) were found to be in common to the two datasets (Materials S1 section 4 and Figure S15).

In a second set of analyses we compared our RACEarray results with the results of paired end RNAseq analysis of total polyA+RNA from pooled human testes/prostate and brain tissues (Human Body Map 2.0 data). A total of 4,485 and 7,448 gene to gene connections (Figure 5B) were detected by tiling arrays compared to 2,013 and 1,066 chimeric RNAs detected by RNAseq analysis. Of these a total of 150 and 163 gene to gene connections were found to be in common to the two datasets (p<0.0001, Materials S1 section 4 and Figure S16).

#### RT- Independent Validations of RACEarray chimeric transcripts

As stated earlier, a serious concern is the potential that these observations are caused by the capability of RT to switch templates during primer extension leading to technically created chimeric artifacts. This capability of RT has been observed during the RNAseq protocol [21] and in vitro RT-PCR reactions [12]. In addition, the presence of short repeats at the junction sites of chimeric RNAs has also been shown to be associated to the event of reverse transcriptase template switching [12], [22].

In order to evaluate further the origin of the observed chimeric transcripts, two sets of RT-independent validation experiments were performed.

**Figure 7:**
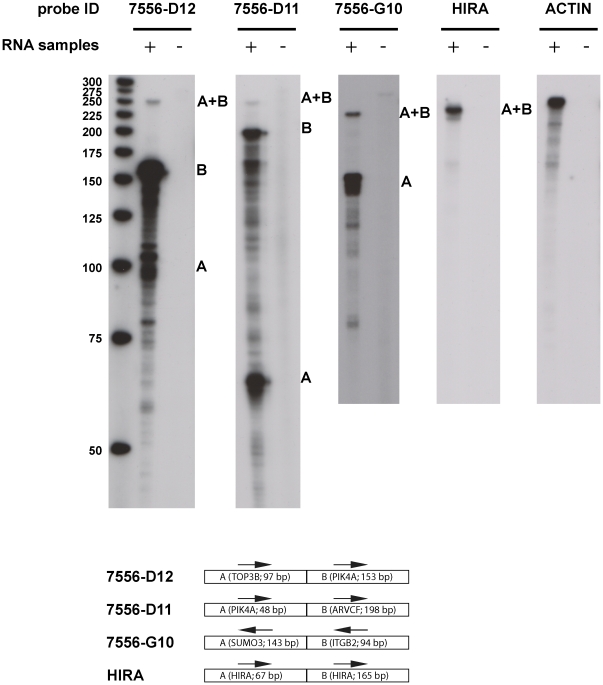
RNAse protection assays to validate predicted fusion/chimeric transcripts. Each panel on top shows the autoradiographs of the probe that covers the predicted chimeric RNA fragment against human RNA from tissue pools detailed in table S2. In each panel, “+” and “−” indicate the tracks with presence and absence of input RNA, respectively. The RNA size ladder is shown on the left of the figure (unit: nucleotides). The bottom of the figure schematically shows the two components of each predicted chimeric transcript. For example, clone 7556-D12 contains 97 bases of an exon of gene TOP3D (designated A) joined to a 153-base exonic fragment of gene PIK4A (designated B); both fragments are in the same genome strand orientation (shown by arrows). In the top first panel corresponding to clone 7556-D12, the protected chimeric fragment in the RNA“+” lane is labeled A+B. The protected (non-chimeric) exons B and A are also shown. The other 2 validated chimeric fragments are shown in the second and third top panels. The fourth panel contains a control exon-exon junction of gene HIRA. The fifth panel contains a control actin gene fragment. A total of 3 out of 15 predicted and tested chimeric fragments were validated using this method. For more details on these experiments see Material and Methods, and for the genomic coordinates of the fragments see table S2.

The first set consisted of RNAse protection assays (RPA). Although the level of sensitivity of this hybridization-based assay has conventionally been understood to be considerably lower than traditional RT-PCR [23], [24], 15 chimeric sequences validated by the RT-PCR and cloning assay were selected to undergo RPA. Evidence of presence of molecules whose structure is clearly chimeric in the pools of RNAs tested was found for 3 out of these 15 cases (Figure 7, and Table S2). This confirmation rate is understandable given the limited sensitivity of the method and the low copy number of the chimeric transcripts. Interestingly, two out of the three cases that were validated by RPA exhibit short duplicated sequences (see Figure 6D), thus indicating that such duplications are not necessarily confirmatory hallmarks for template switching of RT.

The second set consisted of estimating the amounts of possible contribution of technical artifacts to our results. These experiments are based on the analysis of RNAseq libraries prepared from mixtures of human GM12878 cell and Drosophila melanogaster adult female RNAs. This strategy allowed us to analyze in isolation a control population of chimeric reads consisting exclusively of technical artifacts (inter-genomic reads), and to compare its properties to that of our populations of interest (intra-genomic chimeras).

We first analyzed two libraries prepared from pure total RNA from either human or fly, and one library prepared from a 1∶1 mix of RNA from both sources. We obtained about 10 M reads per library that could map uniquely to either of the two reference genomes. Figure S17 summarizes the results. The values are normalized for each library, and expressed in reads per 10 million uniquely mapped reads. Based on at least 25 bases mapping on each side of a chimeric junction, a very small number of inter-genomic reads in the pure human library (9 reads), and a somewhat greater number in the pure fly library (350) were detected. These populations consist exclusively of sequencing and mapping artifacts – an interpretation supported by the fact that their size shrinks dramatically as we increase the stringency of our mapping criteria, requiring larger portions of the reads to map on either side of the junctions. In the mixed library, the number of inter-genomic reads (831) is about 4.6 times the mean of these two numbers (180), which indicates that roughly 20% of the inter-genomic reads in this dataset are explained by sequencing/mapping errors, the other 80% being contributed by library preparation artifacts. Under a null model that all chimeric reads are artifactual, the number of intra-genomic chimeric reads for each species in the 1∶1 mix library should be half the number of inter-genomic reads. The fly population conforms roughly to this expectation, and therefore seems largely accounted for by technical factors. The human population, on the other hand, exceeds the expectations by a factor of 5 (e.g., 2,050 reads instead of an expected 831/2 = 415) – suggesting that a number of chimeric sequences may be present in the human transcriptome. More stringent mapping leads to identical conclusions.

Considering that this simple null model may not accurately describe the generation of artifactual chimeric reads, and that some factors – such as sequence similarity, for instance – may favor the formation of intra-genomic artifacts, we had also sequenced additional libraries designed specifically to distinguish between molecules of technical versus biological origin within the population of human chimeric reads. All were prepared from a mix of human and fly total RNA, keeping the total amount of RNA constant but varying the human-to-fly ratio from 1∶1 down to 1∶100. In this type of dilution series, molecules of biological origin and library preparation artifacts are expected to have drastically different behaviors. The number of the former should decrease linearly with the dilution factor, whereas that of the latter should decrease linearly with the square of the dilution factor. Using the most stringent mapping criteria (min 2×25 bases) the decrease is very close to linear (i.e. 2,050 expected, 2,050/5 = 410 & 2,050/50 = 41 reads, and observed 2,050, 317 & 51. The most stringent mapping policy shows a slightly faster-than-linear decay, revealing a minor artifactual component. These results further support the notion that the human transcriptome does naturally include some chimeric molecules.

### Chimeric RNAs form non-random interconnections between genes suggesting they should be collectively studied as RNA networks

The detection of chimeric transcripts connecting distal gene loci poses a question of whether these gene to gene interactions are random. Several lines of evidence suggest that these connections are not random. First, across all tissues and cell lines examined, the median number of the reciprocal gene-gene RNA connections is 9 and the maximum number observed is 33 (adenosine deaminase, RNA-specific, B1 gene [ADARB1]). This number of connections is statistically greater than a median of 2 that is expected in a random network (Figure S12). More generally, the degree distribution of the gene to gene connections exhibits a broad tail indicating that many genes are poorly connected while few are highly connected (see Materials S1 section 5d.i).

**Figure 8:**
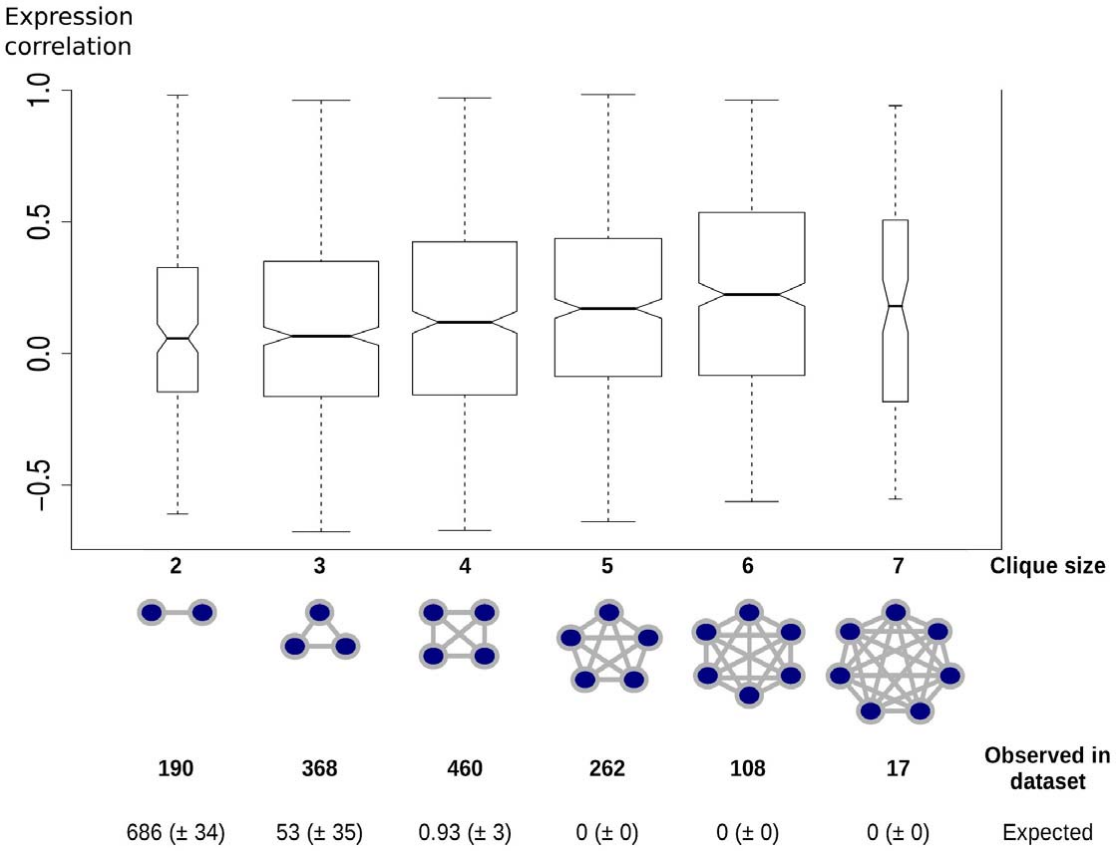
Gene expression coordination within cliques as a function of clique size. The width of the box plots is proportional to the number of connections involved in cliques of a given size. The number of observed cliques of sizes 2 to 7 is reported, as well as the numbers in randomized networks (mean, +/− standard deviation), in the form of a table at the bottom. Randomized networks are generated so that they have the same degree distribution as the original network (see Materials S1).

Second, a detailed examination of the gene to gene interconnections indicated that the network was enriched in “cliques”, that is, sets of genes that are all pairwise connected (see Materials S1 section 5e.i) (Figure 8). At total of 368 cliques were observed mapping to either chromosomes 21 or 22 of size 3 (7-fold the number expected by chance), which included 190 out of the total 2,324 distinct reciprocal gene to gene connections observed across all cell lines/tissues. Also observed were 460 size 4 cliques (460-fold the number expected by chance), which included 487 of the reciprocal gene to gene connections (for the over-representation of cliques, see Materials S1 section 5e.ii and Table S5). Several of the cliques were found in multiple cell lines/tissues; among these were a total of 11 cliques conserved in two cell types, while one clique was found in three cell types (see Materials S1 section 5e.iii and Table S6).

Such properties of non-random gene to gene connections and the structure of these interconnections to form cliques show characteristic of many biological network [25].

#### Transcriptional network hubs tend to be highly expressed and evolutionary conserved

A set of 74 highly connected genes were identified in this analysis that exhibited a substantially larger number of interconnections than expected given their length and the underlying network structure (Materials S1 section 5 d, Figure S13 and Table S3). These genes are referred to as “hubs”. To filter out that hubs could correspond to genes belonging to highly similar paralogous families and to confirm that the observed enrichment of connections is not the result of cross hybridization involving gene family members, we identified RACEfrag sequences that could have potentially been mis-mapped because of cross-hybridization. No enrichment of potentially cross-hybridized RACEfrags in hubs versus non hubs was identified (see Materials S1 section 5 d.iv).

**Figure 9:**
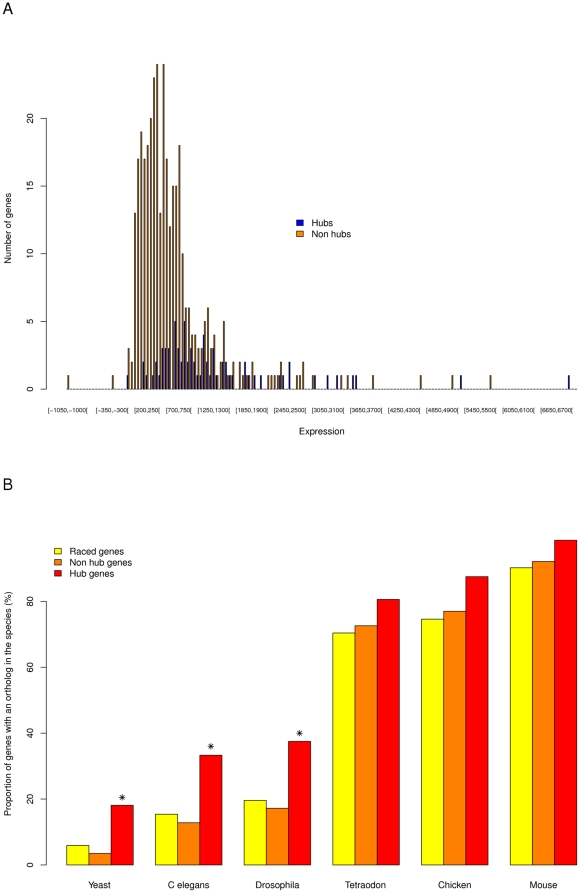
Characteristics of hub genes. A- Expression of hub genes. The distribution of expression of the 74 hubs and of the 362 non hubs is plotted in blue and orange respectively. The expression of a gene is computed based on tiling array experiments performed on the same 16 cell lines and tissues as the RACE experiments (see details in the text). As we can see hubs tend to have higher expression values than non hubs. **B-** **Phylogenetic conservation of hub genes.** In each of the three gene network categories (*i.e.*, hubs, non-hubs, and all RACEd genes), the proportion of genes having a detected ortholog in each eukaryotic species represented on the X axis (ordered by decreasing phylogenetic distance from human) is reported on the Y axis. Instances where the proportion of orthologs found in the *hub* category is significantly higher than for *non-hubs* (*p*<0.01, Fisher test) are marked with an asterisk.

Interestingly, the identified hubs appear to be more highly expressed than other genes. To measure gene and transcript expression we performed hybridizations of the 16 RNA samples onto chromosomes 21 and 22 (see Materials and Methods). From the hybridization signal, we computed the average expression of the gene's coding exons. The expression of an exon is the average over all intensities of the tiling array probes overlapping the exon. We finally computed the average expression of a gene over the 10 different samples (see Materials S1 section 5 d.ii). Figure 9A compares the distribution of average gene expressions in hubs and non hubs.

Finally, hub genes appear to be evolutionarily older than genes that are not hubs. ENSEMBL was interrogated using Biomart (http://www.biomart.org), to identify potential orthologs of all our RACE interrogated genes. This was done by examining six different eukaryotic species representing a panel of increasing evolutionary distances from human species: *Saccharomyces cerevisiae, Caenorhabditis elegans, Drosophila melanogaster, Tetraodon nigroviridis, Gallus gallus *and *Mus musculus* (Materials S1 section 5 d.iv). The hub genes display greater numbers of recognizable orthologs in other species compared to non-hub genes—although the differences are only statistically significant for non vertebrate species (figure 9B). To rule out the possibility that the deeper phylogenetic depth observed for hub genes is an indirect consequence of their higher expression level (and of older genes being more highly expressed), a randomization test was performed in which hub genes were partitioned in four expression classes. The same number of non-hub genes was randomly picked in each expression class. The results indicated that hub genes have greater number of orthologous genes in each of the six species as measured in each of the expression classes (see Materials S1 section 5 d.iv and Table S4).

#### Connected genes tend to exhibit coordination of expression

We have found that transcriptional connected genes tend to have coordinated expression. For each gene, we monitored expression over 16 tissues (Material and Methods); we therefore computed the correlation of expression profiles for each gene pair. We found that connected genes exhibit a higher correlation of expression profile than non-connected genes (0.2 vs. 0.07, see Materials S1 section 6 and Table S7). While such genes are also closer on the genome (1.6 Mb vs. 6.1 Mb), we found that genome proximity could not be considered as an indirect cause for the relation between connectivity and expression coordination (ANCOVA, p-value = 1.24*10^−40^).

We further examined if this result was reinforced for genes involved in a greater numbers of connections. We established that genes within cliques had a higher coordination of expression than genes simply connected but not belonging to cliques. Furthermore, we found that genes within cliques of size 4 and larger had a higher correlation of expression profiles than genes within cliques of smaller sizes. Overall, the larger the clique, the higher the correlation of expression profiles (R = 0.122, p = 4.1*10^−9^, Figure 8). Again, genomic distance was not a confounding factor for the relation between connectivity and expression (Materials S1 section 6). It is important to note that the levels of expression for genes involved in cliques varied considerably although remained coordinated.

#### Connected genes reside in close physical proximity in the nucleus

**Figure 10:**
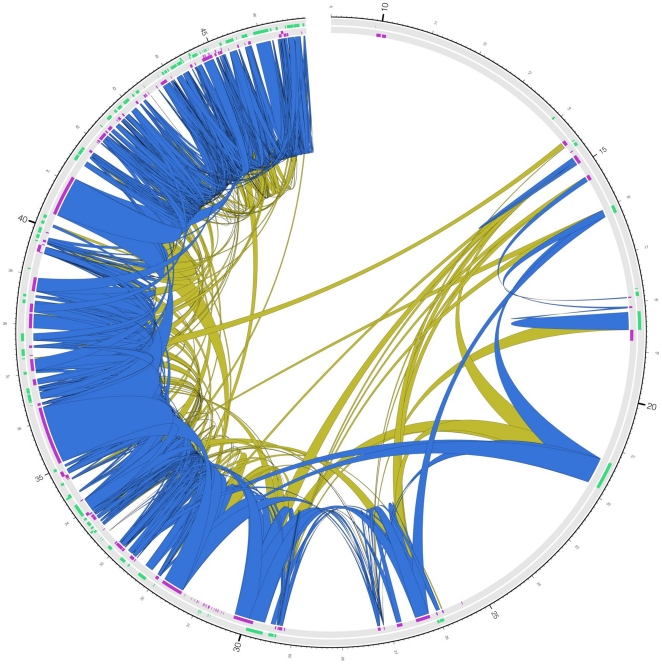
Reciprocal gene/gene connections supported by 5C on chromosome 21. The 638 reciprocal gene/gene connections on chromosome 21 are represented as blue inner links if they are supported by 5C, and as yellow inner links otherwise.

Further underscoring the non-random interconnections seen involving genes comprising the structure of the observed chimeric RNAs, results from studies designed to reveal regions of genomes which in vivo are positioned close to each other (i.e. using Carbon-Copy Chromatin Conformation Capture (5C) method [26] indicate that a statistically significant number of genomic regions encoding genes involved in chimeric RNA synthesis are close to each other in 3D space. Two cell lines were used in this topological analysis: GM06990 and K562. Considering only 785 gene to gene connections (top 10%) with the strongest evidence for being involved in forming chimeric transcripts on chromosome 21 in the respective cell lines, a total of 496 (78%) were also supported by 5C interactions (p-value<10^−3^) (Figure 10, see Material and Methods and Figure S18).

### RNA Chimeras may contribute to expand the protein coding capacity of the human genome

Finally, a total of 208 cDNA sequences resulting from our RT-PCR validation experiments focused on the chimeric junctions were examined for open reading frames (ORFs) with the result that 42% (87) such sequences included at least one ORF in at least one of the six translational frames (Figure 6D) (p = 0.01, Materials S1 section 7). These results while preliminary are consistent with the possibility that chimeric transcripts may be translated into fusion proteins. To further investigate this hypothesis we focused in the subset of 61 chimeric sequences which maintained the annotated CDS sequence and reading frame of the 5′ (N-terminal) gene. The characterization of the chimeric isoforms included the following aspects: domain characterization, binding site mapping, structure modeling, trans-membrane segment and signal peptide prediction. Based on the manual curation of this information we have identified: 5 examples that were previously identified in protein sequence databases (Uniprot), 4 examples in which the proteins encode new dual combined functions (Figure S20), and 7 examples where the fusion of the two proteins will likely lead to a changed localization (Materials S1 section 8 and Figures S18 and S19).

## Discussion

The data from these studies raises several questions concerning the potential biological function(s) and the mechanism of their formation. While a definitive answer to these questions is not available as yet, the significant enrichment of exonic sequences derived from coordinately expressed genes, which often are part of multigenic networks, seems striking and suggestive of an underlying biological function. However, it is important to emphasize the limitations of this primer pooling approach. Because the chimeric RNAs cover very large distances in genome space, unequivocal assignment of a RACEfrag to a primer/gene is problematic. Thus this approach should contain a non-negligible number of false positive assignments—artificially favoring proximal ones. This fact strongly mandates and emphasizes that after additional filtration (e.g. using reciprocal RACE reactions) and biochemical validation (non-RT dependent experiments) is carried out on the candidate chimeric RNAs, the resulting observed chimeric transcripts should possess non-random and biologically meaningful characteristics.

Additional experiments such as capturing the chimeric RNAs by hybridization to junction oligonucleotides followed by high throughput sequencing could be done, but this approach does not avoid the problem of RT template switch. The direct, RT-independent sequencing of RNA molecules, as recently described [27], is an attractive alternative, but currently this approach is plagued by the inconvenience and mapping uncertainty of very short reads from the existing instruments.

The functional attributes of the chimeric transcripts could be in theory evaluated by disruption or silencing of these RNAs. However, the phenotypic readouts of these experiments are largely unknown and unpredicted, and one could use a litany of cellular assays in order to identify a potential phenotypic signature.

The statistically significant interconnections of genes involved, the greater phylogenetic depth of the genes involved in many chimeric interactions, the coordination of the expression of connected genes and the close *in vivo* and three dimensional proximity of the genomic regions being transcribed and contributing to parts of the chimeric RNAs all point to candidates that are likely not to be caused by technical artifacts and biologically important.

Importantly, the synthesis of chimeric RNAs can be envisioned to provide a molecular record of transcribed regions. Speculatively, functions of such an accounting system or the cellular processes could be myriad. One such function could be to provide a means to feedback information to regulate transcription or post-transcriptional levels of the transcripts involved in chimeric RNA production. Evidence from the 5C experiments pointing to co-localization of the genomic regions giving rise to chimeric RNAs suggests the opportunity that the origin of these RNAs may be associated with transcription factories [28] and the mechanism of their formation may play a part in the establishment or maintenance of these sub-nuclear compartments [29].

Several features of the characterized chimeric RNAs suggest that the mechanism(s) involved in their formation is (are) likely not to be limited to the canonical splicing processes. These features include: 1) the very long genomic distances separating some of the exons found in the chimeric transcripts; 2) the presence of chimeric transcripts containing sequences from different chromosomes (Figure 6D); 3) the decrease in the utilization of canonical splicing signals in transcripts with large genomic distances separating exons; and 4) the occurrence of short repeat sequences bracketing the junction sites of chimeric transcripts. Additional information concerning the mechanism(s) underlying the formation of these RNAs could potentially come from analysis of the proteins found associated with them in immunoprecipitation experiments.

Finally, observations of the existence of chimeric RNAs in not novel [5], [9], [11], [30], [31]. With these reports there have been studies published warning of potential technical [22], [32] and biological reasons [3], [9] that could be the underlying causes of these earlier observations. These confounding causes include: template switching capabilities of RT, mis-mapping of sequence or tiling array results and cryptic genomic rearrangements present in the samples analyzed. These confounding issues are real and serious. However, considerable effort has been taken to evaluate and estimate the level of the false positive occurrences present in the data presented. Overall, the presence of chimeric RNAs as molecular events present in normal tissues and cell lines is strongly supported and while their biological importance is uncertain, a number of characteristics of the observed RNAs argue for them to be functional. The results of pending genetic and biochemical studies are required to provide a definitive answer to this question.

## Materials and Methods

### Design of the RACEarray experiments

#### Selection of genes (annotation)

The high-quality HAVANA annotation [18] was used as a reference for chromosome 21. Due to its unfinished nature on chromosome 22 at the time of the experimental design (only the first 35 5′-most clones had been thoroughly re-annotated at that time), we merged it with its VEGA counterpart [33] on this chromosome, removing redundancy between the two annotation sets. The exhaustiveness of the HAVANA annotation is reflected by the numbers of annotated alternative isoforms per gene in both chromosomes: while in chromosome 21 there is an average of 5.4 annotated transcripts per gene, this number is as low as 1.34 on chromosome 22. For both chromosomes, only protein-coding transcripts were targeted in the experiment. The list of manually annotated exons used in this study is provided at the following address:

ftp://genome.crg.es/pub/Encode/ChimericRNAChr2122/suppl_materials_manual_annotation.zip

#### Selection of target exons and RACE primers

RACE primers were designed within what we call Atomic Coding Exon Projections (ACEPs), namely, coding exon segments not interrupted by any annotated exon boundary. This ensured that all RACE primers would match an actual exon sequence, rather than a “collapsed” version of many.

In order to limit cross-hybridization of RACE products, all ACEPs were searched in the entire human genome for possible repeats with BLAT [34]; gfClient/gfServer version with default parameters, except: tileSize = 10, stepSize = 5, minScore = 0, minIdentity = 0). Only those ACEPs having only one genome-wide match (i.e., a BLAT hit with more than 50% nucleotide identity over at least 50% of its length) were kept. Surviving ACEPs were then fed into the primer3 program [35] to design a maximum of 100 alternative RACE primer sequences per unit and per RACE type (5′ and 3′), with the following parameters: 23≤primer size≤27, optimal size = 25, 68°C≤primer Tm≤72°C, optimal Tm = 70°C, 50%≤primer GC percentage≤70%. Within each ACEP, 5′ and 3′- RACE primers obtained were separately ranked according to primer3′s quality score. Then, and for each type of RACE, only the best unique element of each ACEP (i.e., best primer3-ranked element satisfying the following condition: only one genome match with more than 95% sequence identity over at least 50% of its length, and at least one of its three 3′-most nucleotides matching) was included in a set of potential RACE primers.

To achieve an optimal, balanced final distribution of primers along genes (ref), the following procedure was applied, to both 5′- and 3′-RACE potential primer sets:

- for genes whose virtual CDS sequence (i.e., concatenation of ACEPs) was shorter than 1.2 kb, only one putative RACE primer per gene was picked, as close as possible to the middle of it.
- when the virtual CDS sequence of a gene was longer than 1.2 kb, the two most extreme primers were selected. If these two were separated by more than 1.2 kb of virtual CDS sequence, a third primer was picked, as close as possible to the middle of it.

This resulted in a final set of primers, consisting in 844 5′-RACE and 824 3′-RACE oligo-nucleotides, targeting 492 genes in total (see detailed statistics in figure 1). Performing an exhaustive search of these 1,668 primers along the genome using the GEM program (http://gemlibrary.sourceforge.net/), we find that all of them have a unique hit in the genome with no mismatch, 3 of them have a hit in more than one location with 1 mismatch, and 31 with two mismatches.

The list of primers is provided in gff format at the following address:

ftp://genome.crg.es/pub/Encode/ChimericRNAChr2122/suppl_materials_RACE_primer.zip

### Pooling of RACE reactions

Ideally, a distinct RACE reaction is realized per primer, and the corresponding products are hybridized on a single tiling array. In practice, this is not feasible in large-scale projects (for instance, in the project here it would have required more than 25,000 tiling array hybridizations). A cost effective strategy is to pool together RACE products prior to hybridization. This compounds, however, the assignment of RACEfrags to the originating primers. To facilitate the assignment, it is desirable to maximize the genomic distance between the primed regions of the RACE experiments that are pooled together, so that each resulting signal from the array can be assigned to its corresponding primer as unambiguously as possible. The composition of each pool in terms of RACE primers is thus designed with the aim of simultaneously achieving two goals: Minimizing the number of pools (and consequently the cost of the experiment), and maximizing the genomic distance between the genomic positions of the primers that are pooled together.

In order to address this optimization problem, the pooling strategy is organized in two steps. First, i/Given the genomic positions of the primers, compute the minimum number of pools required in order to guarantee a minimum distance between two consecutive primers from the same pool. The longer the distance, the more the pools. ii/Given this total number of pools, distribute the primers within pools, in order to maximize the distance between each two consecutive primers. The constraint is the minimum distance between primers. This depends on the orientation of the primers. Indeed, as primers should only allow amplification from their 3′ ends, the minimum distance from their 5′ end can be lower than from the 3′. Two minimum distances can thus be defined: distance L in 3′ of the primers, and distance l in 5′ of the primers. In practice, primers boundaries are initially extended accordingly (i.e., by L base pairs in 3′ and by l base pairs in 5′) before being input to the pooling process. The values that we employed were L = minimum distance from 3′: 900,000 bp, and l = minimum distance from 5′: 90,000 bp.

The two steps of the algorithm are described in more details in Materials S1 section 1.a. The minimum number of pools is estimated independently on chromosome 21 and 22. The largest of the two pool number values is used independently in each of the chromosomes to distribute the primers along the chromosomes. Note that primers from different chromosomes are pooled together. We implicitly assume thus that there are not inter-chromosomal RNA chimeras.

In our case, given the distance constraints above, we ended up with 102 pools. Figure S1 provides the distance distribution between consecutive pooled primers according to their relative position and chromosome.

### RACE reactions and RACEarray hybridization

5′ and 3′ RACE reactions were performed as published [5], [6](refs). Briefly, we performed 5′- and 3′ RACEs of 1,193 ACEPs corresponding to 492 genes of human chromosome 21 and 22 on polyA+ RNAs from 11 tissues (Brain Frontal Lobe, Brain Hippocampus, Brain Hypothalamus, Cerebellum, Ovary, Placenta, Prostate, Testis, Fetal Kidney, Fetal Spleen, Fetal Thymus; all from BD Clontech) and 5 cell lines (GM06990, HeLaS3, HepG2, K562, Tert-BJ; all from American Type Culture Collection (ATCC)) using the BD SMARTTM RACE cDNA amplification kit (BD Clontech Cat. No. 634914). This combination of 16 cell types was previously shown to be the optimal combination in a set of 48 tissue/cell lines [6] allowing to capture about 80% of the transcript diversity. Double-stranded cDNA synthesis, adaptor ligations to the synthesized cDNA were performed according to the manufacturers' instructions. The 26,688 RACE reactions were performed in a 12.5 µl final volume, set up in a 384 well plate format using a Freedom EVO 200 robot (TECAN) and run in a BioRad DNA Engine Tetrad following the conditions suggested by the provider.

RACE reactions were pooled in two ways. First, between 14 and 19 reactions (mean 16.4) from a single tissue/cell line were pooled together respecting the greatest possible genomic distance between the different primers used (see above, “Pooling of RACE reactions” section) to create a set of 102 pools for each tissue/cell line. Second, for cost efficiency the same pools from Prostate and Testis, Ovary and Placenta, Brain Frontal Lobe, Brain Hippocampus and Brain Hypothalamus, as well as Fetal Kidney, Fetal Spleen and Fetal Thymus were pooled before being hybridized to the tiling arrays. Thus this two-step procedure generated a set of 1,020 pools.

RACE PCR reactions were pooled, purified using QIAquick PCR purification kit (Qiagen) following the vendor's instructions, ethanol-precipitated, labeled and hybridized to Affymetrix Chromosome 21/22 2.0R arrays (P/N 900936) as previously described [6].

### Optimization of RACEfrag calling

RACEfrags are usually called from Affymetrix tiling array probe intensities using TAS (http://www.affymetrix.com/support/developer/downloads/TilingArrayTools/index.affx). In order to address the issues of pool-unspecific RACEfrag (namely, RACEfrags that repeatedly appear in many different unrelated experiments) and unspecific probes (we observed that 7% of the probes in chromosome 21–22 tiling arrays had multiple exact matches in the genome), we discarded array signals coming from these two types of unspecific probes and optimized the parameters of the RACEfrag calling, using a RACEfrag caller developed in-house (software available upon request). The caller depends on four parameters: (1) the percentile intensity threshold I above which we consider a probe to produce a positive signal. Given this threshold, the set of positive probes can be defined for a given experiment, (2) the maximum number nucleotides that is allowed between two consecutive positive probes to be included in the same RACEfrag, (3) the minimum number of probes that are needed to call a RACEfrag, and (4), a binary flag that determines whether RACEfrags are called from the middle of the first probe to the middle of the last probe, or from the start of the first probe to the end of the last probe.

The RACEfrag caller is described in detail in Materials S1 section 2.

### RACEfrag filtering

In order to eliminate RACEfrags coming from unspecific priming and/or unspecific cross-hybridization, we used our in-house *in silico* RACEarray simulator [6]. This simulator starts from a known set of transcripts, primers and array probes, and generates simulated RACEfrag maps from which we can confidently discriminate between *bona fide* RACEfrags (i.e., originating from specific priming and specific array hybridization) and artifactual ones (i.e., arising from RACE mis-priming and/or array cross-hybridization). The RACEarray simulator is described in detail in Materials S1, see also Figure S4).

On the other hand, transcripts that have not been targeted by any RACE primer but that are present at high levels in a given tissue may provide artifactual signals on the corresponding tiling array, and thus give rise to unspecific RACEfrags in this tissue. In order to eliminate such RACEfrags, we performed negative control experiments, i.e., exactly the same RACEarray experiments without any specific RACE primer in the mixture on which RACE reactions are performed (the mixture only contains Master mix, buffer, polymerase, unspecific primers, cDNA, dNTP), and with more sensitive parameters for RACEfrag calling (I = 99%ile, M = 25 bp, m = 3 probes, c = middle to middle). These negative control experiments thus provide us with 10 RACEfrag sets, one for each of the 10 tissue pools, which are then subtracted to the RACEfrags remaining from the USPP filter in a tissue specific manner. More precisely, any RACEfrag overlapping a negative control RACEfrag of the same tissue is eliminated. On the 302,699 RACEfrags remaining from the USPP filter, 40,227 were eliminated by the negative control filter (14%), and we kept the remaining 262,472.

### RACEfrag assignment

As a given experiment (pool) corresponds to the hybridization onto a tiling array of a mixture of products obtained from RACE reactions performed with several different RACE primers, the information as to which RACEfrag comes from which primer of its pool is not known a priori. Still, it is crucial to be able to assign an originating RACE primer to each RACEfrag, in a pool-by-pool fashion.

RACEfrag assignment is an optimization problem; however we lack a proper underlying model to solve it as such. We instead use heuristic rules based on the three following remarks or definitions: i) a given pool includes primers of different chromosomes and pointing in different directions (5′ and 3′ RACE primers located on genes on the forward and the reverse strands); ii) within a pool, a primer is said to be compatible with a RACEfrag if it is located on the same chromosome as the RACEfrag and is pointing in its direction; iii) a primer is said to be active in pool p and tissue t, if it is overlapped by a RACEfrag that appears in p and t.

Given a RACEfrag r of pool p and tissue t, if the closest compatible primer of pool p is active in p and t, then we assign it to r, else no primer is assigned to r and r is eliminated from the rest of the study.

The fact that several tissues or/and several primers per gene are used in the RACEarray strategy can be utilized to attribute a confidence score to the assignments (i.e., RACEfrag/locus pairs). More precisely, for any RACEfrag r and locus l, we define the assignment confidence score of the pair (r,l), ACS(r,l), as:
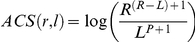



Where:

R is the number of times RACEfrag r appears in total (in all experiments),
L is the number of times r is assigned to a primer of locus l,
P is the number of primers of locus l to which RACEfrag r is assigned.

L is raised in order to favor RACEfrags appearing in multiple experiments: it is raised to the power of P+1 to favor RACEfrags assigned to multiple primers from the same locus, while not penalizing too much RACEfrag-locus pairs co-occurring only in a small number of pools. Raising R to the power of one plus the number of times RACEfrag r is not assigned to l greatly penalizes “ubiquitous” (unspecific) RACEfrags, while taking into account the number of times r appears. As a result, a RACEfrag r is all the more confidently assigned to a locus l as the corresponding assignment score is low.

Figure S5 illustrates the assignment of primers to RACEfrags as well as the scoring of these assignments. On the 262,472 RACEfrags that remained from the negative control filter, 195,982 (75%) could be assigned and thus constitute the RACEarray map used in the rest of the analyses.

The list of these RACEfrags is also provided in gff format at the following address:

ftp://genome.crg.es/pub/Encode/ChimericRNAChr2122/suppl_materials_RACEfrags.zip

### Tiling array hybridizations

PolyA+RNA from the same 16 cell types as described above hybridized to Affymetrix Chromosome 21/22.0R arrays (P/N 900936) as previously described [6]. Each probe of the array is thus associated to a signal or intensity value in each of the 16 cell types, which is then used to associate an intensity to each projected exon and gene in the 16 cell types. The intensity of a projected exon is obtained by taking the mean of the intensities of the probes overlapping it, and the intensity of a gene is obtained by taking the mean of the intensities of its projected exons. This data is provided at:

ftp://genome.crg.es/pub/Encode/ChimericRNAChr2122/suppl_materials_gene_expression_profiles.zip

### RT-PCR, cloning and sequencing of RACE connections

#### Selection of cases for experimental verification

We performed RT-PCR, cloning and sequencing on 245 RACE connections (200 of which consisted in gene-gene links, the rest comprising intergenic RACEfrag connections), so as to validate them and determine their detailed exon structures. These 245 cases were selected as follows.

All 67 reciprocal gene-gene connections supported by a direct primer-primer link were included in this part of the experiment, together with 178 simple (non-reciprocal) primer/RACEfrag pairs. The latter set underwent the following selection procedure: first, all 195,982 candidate connections from the complete RACEarray map were grouped into two primer/RACEfrag distance bins, (i) 0–350 kb (short) and (ii) more than 350 kb (long). 96 primer/RACEfrag pairs were randomly picked in the short-distance bin. Pairs from the long-distance bin were ranked according to their ACS (see above) and the 82 most confident ones (namely, with a low ACS) were selected for RT-PCR. Within the 245 test cases, 200 corresponded to chimeric cases, namely, connections involving two different annotated loci.

#### Experimental verification

To experimentally verify the RACE-array defined connections (both “reciprocal” and “simple” cases), nested PCR were carried out to increase sensitivity and specificity of the reactions. Amplification was done using two sources of templates: reverse-transcribed RNA from six adult human tissues (brain, testis, liver, placenta, heart and ovary) and the relevant set of original reverse-transcribed RNA used in 3′ and 5′ RACE reactions. Reverse transcription of adult tissue RNAs was done as previously described [36]. The first set of PCR reactions were done (in 50 µl reaction volume) for 40 cycles. Following completion, 0.5 µl of the products were used as templates for the second round of PCR (for 30 cycles) with the nested primers. Products from the nested PCRs were visualized by agarose gel electrophoresis and recombinationally inserted into a Gateway compatible plasmid (pDONR223) using the BP reaction [37], and transformed into chemically competent DH5α E. coli. The transformants were spread on LB-agarose plates containing 100 µg/ml of spectinomycin.

Eight colonies were picked for sequencing from either the RACE-template reactions or the six-tissue RT-PCR clones with priority given to the RACE-template reactions. For cases in which a positive product was not observed in the RACE-templated reactions but product(s) were present in the adult tissue RT-PCRs, the latter were chosen for further analyses. In each case, colonies were grown in deep 96-well plates containing 100 µg/ml spectinomycin LB. A small volume of these cultures served as template to amplify the cloned inserts using universal vector primers. The resulting PCR amplicons were sequenced at Agencourt Bioscience Corp. (Beverly, MA, USA). Forward and reverse sequence reads were vector-trimmed with cross_match (version 0.990329, default options) and assembled using Phrap [38]. In cases where the end reads did not overlap (i.e., a contig could not be assembled), internal primers were synthesized and additional internal sequencing was carried out to generate full-length contigs.

To monitor RT-PCR and cloning efficiency, we carried out amplification of canonical open reading frames from four genes using the six-tissue RNA and RACE templates. These ORFs were from TP53 (expected size 1,182 bp), CIRH1A (2061 bp), ORC1L (2,586 bp), and THRAP4 (2,970 bp). Following Gateway recombinational cloning, 16 colonies were picked (8 from each of the 6-tissue RT-PCR and the RACE templated experiments) and end sequenced. For TP53 and CIRH1A, all colonies (i.e., 16 each) generated expected sequences. For ORC1L and THRAP4, 5 out of 16 clones, and 8 out of 16 colonies yielded the expected sequences, respectively.

Second round of RT-PCR (“primer walking” inside cloned sequences).

A second round of RT-PCR was performed using the same procedure as the one described in the above subsection. However, some clone inserts could not be completely sequenced.

#### Analysis of the sequences

(i) – Genome mapping and RT-PCR success rate.

In total, 3,503 RT-PCR clone sequences (both assembled contigs and singlet reads) were obtained. After trimming off vector sequences and filtering out sequences of less than 100 nucleotides, 1,572 of them remained and were mapped onto the human genome (NCBI 35/hg17 version) using BLAT [34] (default parameters, version 34). 1,551 of them could be mapped. Due to a hard-coded limit in the maximum intron size allowed (750 kb) by BLAT, all hits were further processed to chain sequence parts affected by this issue. The best-in-genome hit was then selected for each mapped sequence, and only those sequences containing the sequences of both internal oligonucleotides from the intended nested PCR were kept – a very stringent filter, since all unfinished clone sequences are discarded at this step. Surviving RT-PCR sequences were subsequently clustered based on their mapped transcript structure, so as to reduce sequence redundancy. This resulted in a total of 208 distinct transcripts, originating from 112 initial chimeric RT-PCR targets, and 931 different RT-PCR products. The overall RT-PCR success rate was thus 56% (112 connections validated by RT-PCR, out of 200).

The 208 clusters of RT-PCR products were fed into the HAVANA manual annotation pipeline [18], using the aforementioned BLAT genome mapping as a basis. Having identified the genomic span of an aligned RT-PCR sequence, a session containing the corresponding region was opened in the Otterlace annotation interface [39] and potential novel splice variants were investigated using its integrated Zmap genome viewer. RT-PCR sequences were aligned to the genomic contig using BLAST [40] and the resulting alignments were navigated using the Blixem alignment viewer [41]. Visual inspection of the dot-plot output from the Dotter tool (ibid.) was used to resolve any alignment with the genomic sequence that was unclear or absent from Blixem. Where very short sequences (<15 bases) are missing from an alignment, a dot-plot is unsuitable due to the difficulty in seeing very short alignments (particularly at the edges of the display) and the Zmap DNA Search tool (essentially a pattern matching tool [42]) was used to try to identify any alignment with the genome.

Where the RT-PCR sequence was shown by any of the approaches described above to support locus-joining canonical splice sites (defined as N|GT-AG, G|GC-AG and N|AT-AC), a novel transcript structure was annotated and tags indicating the coding potential of the variant were added [43]. Detailed annotation statistics are presented in Figure 6D.

A large proportion of the RT-PCR confirmed transcript structures were found to contain small genomic duplications around the locus-joining exon junction (Figures 6C and 6D), a tendency that was all the more important for non canonical introns and for distant locus-joining junctions. More generally we looked at HAVANA manually annotated introns and found that some of them also contain such genomic duplications at the exon-intron and intron-exon boundaries. More precisely out of the 351,490 introns of the July 2008, hg18 HAVANA version, 35,864 (10.2%) contain duplicated sequences ≥5 nt around the donor and acceptor sites, and this proportion is 10.0% for the canonical introns and 20.7% for the non canonical ones.

### RNAse protection assays to confirm chimeric connections found by RACEarray

In order to evaluate the genuineness, RT-PCR independent nature of the chimeric connections, we randomly picked 15 RT-PCR clones arising from reciprocal RACE connections to undergo RNAse protection assays (RPA). Each RT-PCR clone to be tested was sub-cloned into the pDONR223 vector. DNA fragments covering each chimeric junction were synthesized by PCR (100–300 base pairs) using specific primers containing SP6 (forward primer) and T7 (reverse primer). Using this template, antisense and sense riboprobes were prepared with SP6 and T7 polymerase MAXIscript in vitro transcription kit (Ambion). Designed riboprobes were checked for uniqueness using BLAT on the hg18 version of the human genome.

RNase protection assays were performed by using the RPA III kit from Ambion according to the manufacturer's instructions. 500 ng of each Poly(A+) RNA (Clontech) were used per reaction. Protected fragments were separated on a 10% denaturing (8 M Urea) polyacrylamide gel and signals were analyzed by autoradiography. On the 15 chimeric transcripts tested by this method, 3 were found positive, and 2 out of the 3 positive cases were found to include a small duplicated sequence around the tested junction, as shown in table S2. Table S2 is available at:

ftp://genome.crg.es/pub/Encode/ChimericRNAChr2122/tabS2.xlsx

### 5C data validates RACEarray gene to gene connections

In order to know whether some RACEarray reciprocal gene to gene connections could be due to the three dimensional proximity between the two genes involved in the connection, we used 5C studies (Chromosome Conformation Capture Carbon Copy) performed on chromosome 21 on cell lines GM06990 and K562. The 5C technique indeed provides pairs of genomic regions supposed to physically interact. In the rest of the paragraph and for simplification purposes, a reciprocal gene to gene connection will simply be called a gene to gene connection.

Let us first introduce some definitions. A gene to gene connection g1–g2 is:

Detectable by RACEarray if there exists a RACE primer in g1 pointing in the direction of g2 and a RACE primer in g2 pointing in the direction of g1;
Validated by RACEarray if g1–g2 is a reciprocal gene to gene connection;
Detectable by 5C if there exists a 5C pair (FW,RV) such that either g1 overlaps FW and g2 overlaps RV, or g1 overlaps RV and g2 overlaps FW;
Validated by 5C if there exists a 5C pair (FW,RV) in the 10% best 5C pairs, such that either g1 overlaps FW and g2 overlaps RV, or g1 overlaps RV and g2 overlaps FW.

Among the 785 gene to gene connections validated by RACEarray on chromosome 21, 638 are detectable by 5C, and 496 are validated by 5C, yielding a proportion of 77.7% gene to gene connections validated by 5C (496/638). We were thus interested in testing the following hypothesis: “from the gene to gene connections validated by RACEarray (638), is the number of gene to gene connections validated by 5C (496) higher than expected by chance?” To do this, we sampled 1,000 times 638 gene to gene connections within the 8,852 that we find detectable by both techniques while keeping the same distributions of distance between connected genes, and of length of connected genes as in the 638 connections validated by RACEarray (details not shown), and for each sample we computed the proportion of gene to gene connections validated by 5C (see distribution on Figure S18). From these simulations, we found that the proportion of RACEarray gene to gene connections validated by 5C (77.7%) was significantly higher than the proportion one would expect by chance given the distribution of distance and the distribution of length of connected genes (70.8%±1.9%, p-value<10−3). Note that three different thresholds for defining validation by 5C were tested: 5, 10 and 20%, and although all three thresholds were providing significant p-values, better results were obtained for 10%ile, which is the reason why this threshold was chosen (details not shown).

The 5C data used for this analysis is available here:

ftp://genome.crg.es/pub/Encode/ChimericRNAChr2122/suppl_materials_5C_data.zip

### RNA mixture experiment

A series of control experiments were conducted to investigate a possible contribution of technical artifacts – from library preparation, sequencing, or mapping – to our sequencing results. These experiments are based on the analysis of RNAseq libraries prepared from mixes of RNA from different sources –namely, human GM12878 cells and *D. melanogaster* adult females. This strategy allowed us to analyze in isolation a control population of chimeric reads consisting exclusively of technical artifacts (inter-genomic reads), and to compare its properties to that of our populations of interest (intra-genomic chimeras).

We first analyzed two libraries prepared from pure total RNA from either human or fly, and one library prepared from a 1∶1 mix of RNA from both sources. We obtained about 10 M reads per library that our alignment algorithm (STAR, http://gingeraslab.cshl.edu/STAR/ [44]) could map uniquely to either of the two reference genomes. We scored the numbers of intra- and inter-genomic chimeric reads identified in each dataset, under different mapping policies. The numbers reported in the table below are normalized for each library, and expressed in reads per 10 million uniquely mapped reads. If we required a minimum of 20 bases to be mapped on each side of a chimeric junction, we identified a very small number of inter-genomic reads in the pure human library (183 reads), and a slightly greater number in the pure fly library (1,660). These populations consist exclusively of sequencing and mapping artifacts – an interpretation supported by the fact that their size shrinks dramatically as we increase the stringency of our mapping policy, requiring larger portions of the reads to map on either side of the junctions. In the mixed library, the number of inter-genomic reads (1,809) is about twice the mean of these two numbers (922), which shows that roughly half of the inter-genomic reads in this dataset are explained by sequencing/mapping errors, the other half being contributed by library preparation artifacts. Under the null model that all chimeric reads are artifactual, the number of intra-genomic chimeric reads for each species in the 1∶1 mix library should be half the number of inter-genomic reads. The fly population conforms roughly to this expectation, and therefore seems largely accounted for by technical factors. The human population, on the other hand, exceeds the expectations by a factor of 5 (e.g., central row: 2,050 reads instead of an expected 831/2 = 415) – suggesting that a number of chimeric sequences may be present in the human transcriptome. More stringent mapping leads to identical conclusions.

Bearing in mind that this simple null model may not accurately describe the generation of artifactual chimeric reads, and that some factors – such a sequence similarity, for instance – may favor the formation of intra-genomic artifacts, we had also sequenced additional libraries designed specifically to distinguish between molecules of technical versus biological origin within the population of human chimeric reads. All were prepared from a mix of human and fly total RNA, keeping the overall amount of RNA constant but varying the human-to-fly ratio from 1∶1 down to 1∶100. In this type of dilution series, molecules of biological origin and library preparation artifacts are expected to have drastically different behaviors. The number of the former should decrease linearly with the dilution factor, whereas that of the latter should decrease linearly with the square of the dilution factor (as does the probability of independently picking 2 human transcripts by random sampling). With our least stringent mapping policy (minimum mapped length of 2×20 bases) the decrease is less than linear, suggesting that the analysis is confounded by sequencing/mapping artifacts – which we know not to be filtered efficiently by this policy, as explained above. With more stringent mapping (min 2×25 bases) the decrease is very close to linear: we expected 2,050, 2,050/5 = 410 & 2,050/50 = 41 reads, and observed 2,050, 317 & 51. The most stringent mapping policy shows a slightly faster-than-linear decay, revealing a minor artifactual component. These results further support the notion that the human transcriptome does naturally include some chimeric molecules.

## Supporting Information

Materials S1 **Supplementary text.**
(DOC)

Figure S1 **Distance between consecutive pooled primers.** This figure represents the distribution of distances between consecutive primers of the same pool for each chromosome and each relative position of primer pairs: head to head (→ ←), head to tail (→→) and tail to tail (←→), as a box plot. The gene density of chromosome 22 being higher than that of chromosome 21, the distance between consecutive pooled primers is smaller for the latter than for the former. Also, as expected, the distance between consecutive pooled primers is higher for head to head than for head to tail configuration, and higher for head to tail than for tail to tail configuration.
(TIFF)

Figure S2 **Two measures to assess RACEfrag sets.** This figure describes the two measures used to assess RACEfrag sets while optimizing the parameters used for RACEfrag calling: the exonic accuracy and the splice site score. The exonic accuracy assesses the RACEfrag set with respect to a reference set: the projected internal exons. More precisely for each projected internal exon, considered as a reference, the exonic accuracy assesses the accuracy with which the RACEfrags overlapping this reference mimics this reference. This accuracy is measured in terms of intersection over union of the projected internal exons and the RACEfrags, i.e., for each projected internal exon with overlapping RACEfrags, the number of nucleotides in common between the two sets is divided by the number of nucleotides in either of the two sets. The exonic accuracy of a RACEfrag set is then the median of the exonic accuracy of projected internal exons with overlapping RACEfrags. Unlike the exonic accuracy the splice site score of a RACEfrag set does not depend on any reference but is rather intrinsic to the RACEfrag set. More precisely, the spice site score is divided into two sub-measures: the acceptor score and the donor score. Both scores involve the scanning of two windows around the RACEfrag boundaries, W1 around the left boundary and W2 around the right boundary, where both acceptor and donor sites have previously been found by the geneid program. The RACEfrag acceptor score is then defined as the score of the best acceptor site on the 2 windows, and the RACEfrag donor score as the one of the best donor site on the 2 windows.
(TIFF)

Figure S3 **RACEfrag calling.** This figure represents the exonic accuracy of 10 RACEfrag sets coming from 10 randomly chosen experiments, as a function of the intensity threshold (I) and the maxgap (M), for 3 different minrun values (m): 3, 4 and 5. The blue arrows indicate the maximum exonic accuracy found over all the possible values of the three parameters, and the red arrows the minimum exonic accuracy. The maximum is reached for I = 99.1%ile, M = 59 bp, m = 5 probes.
(TIFF)

Figure S4 **USPP filter.** This simulation involves two steps: (1) RACE from a set of primers and a set of known transcripts; (2) hybridization of the obtained RACE products on tiling arrays. The RACEarray simulator generates a set of tiling array probes that are highlighted by the RACE products and that we call simulated positive probes (SPPs). These SPPs can be further divided into two categories: (1) bona fide SPPs, i.e. overlapping an exon of the target locus; (2) unspecific SPPs, also called USPPs, i.e. mapping outside of the target locus exons. In our model these USPPs correspond to false positives that originate from RACE mis-priming and/or from array cross-hybridization (see text for a more detailed explanation).
(TIFF)

Figure S5 **RACEfrag assignment.** This figure is divided into three parts: 1) on the top, the annotations of a given chromosome are represented, which are here the different alternative transcripts of three loci: A, B and C; 2) in the middle, the primers and RACEfrags of three different pools in several tissues are represented; 3) on the bottom, the formula of the assignment confidence score is provided again as well as its application on 5 different (RACEfrag, locus) pairs (note that here two RACEfrags with the same coordinates are given the same identifier). The first two parts of the figure are thus dedicated to the description of the assignment method, while the third part shows how the assignment score behaves on already assigned RACEfrags associated to their locus. Primers are named and colored after the locus they are originating from, and RACEfrags after the locus they have been assigned to. In pool 1, primer C1 is active and points in the direction of all RACEfrags, so all RACEfrags of pool 1 are assigned to primer C1. In pool 2, it is the same with primer C2, and in pool 3 the same with primer A1. Then the ACS formula is applied to 5 different (RACEfrag, locus) pairs, and the lower the score the more confidence we have in the assignment of the RACEfrag to the locus. Here the (RACEfrag, locus) pair we are the most confident in is (3,C) since RACEfrag 3 appears 4 times in total and each time it appears it is assigned to locus C. Also, the fact that it is assigned to two different primers of locus C, primers C1 and C2, strengthens the confidence we have in this pair. The pair (4,C) is similar to the pair (3,C) except that RACEfrag 4 appears in 2 experiments instead of 4. It thus also has a good score, although less than the one of (3,C). The pair (2,C) is like the pair (3,C) except that RACEfrag 2 also appears in pool 3, tissue 1 where it is assigned to locus A. This makes it more uncertain we should assign RACEfrags 2 to locus C, as compared to RACEfrag 3, and this is why the score of (2,C) is lower than the one of (3,C). The pair (5,C) is similar to the pair (4,C) except that RACEfrag 5 is only assigned to 1 primer of locus C (primer C1), compared to two primers of locus C for RACEfrag 4 (primers C1 and C2). This explains the lower score of (5,C) with respect to the one of (4,C). Finally the pair (2,A) is given a very bad score since RACEfrag 2 appears 5 times but is assigned only once to locus A.
(TIFF)

Figure S6 **Chromosome 21 transcriptional networks.** RACE connection networks in all 10 assayed cell types are represented. In each plot, the chromosome is depicted as a circle, and RACEfrag connections as inner links between genomic regions (5′ and 3′ RACE connections are red and blue, respectively). The circular tracks are, going inwards: (1) - chromosome scale (in megabases, starting at 14 Mb), (2) - plus-strand annotated genes (green), (3) - plus-strand annotated pseudogenes, (4) - minus-strand annotated genes, (5) - minus-strand annotated pseudogenes.
(JPG)

Figure S7 **Chromosome 22 transcriptional networks.** See legend of figure S6.
(JPG)

Figure S8 **Reciprocal gene to gene connections in chromosome 21 (A) and 22 (B).** All 2,324 pure and composite gene/gene reciprocal connections observed in the 10 cell types studied are represented as blue (connection involving two genes on the same chromosome strand) and orange (connection involving two genes on different strands) inner ribbons. See Figure 2A for further legend details. Pseudogene tracks were removed for clarity purposes (See Figures S9 and S10 for reciprocal gene/gene connections in each cell type).
(JPG)

Figure S9 **Reciprocal gene to gene connections observed in each cell type on chromosome 21.** Networks of reciprocal gene to gene connections observed in each of the 10 assayed cell types are represented as blue (connection involving two genes on the same chromosome strand) and orange (connection involving two genes on different strands) inner ribbons. See Figures S6 and S7 for further legend details. Pseudogene tracks were removed for clarity purposes.
(JPG)

Figure S10 **Reciprocal gene to gene connections observed in each cell type on chromosome 22.** See legend of Figure S9.
(JPG)

Figure S11 **Pairwise correlations between cell types based on pure reciprocal gene to gene connections.** This figure represents the pairwise correlations between the cell types used in the RACEarray experiments as a heatmap: the closer to the white, the more correlated. More precisely for each pair of cell types, the Pearson's product moment correlation between them was computed based on the number of reciprocal gene to gene connections commonly observed, in the universe of all possible reciprocal gene to gene connections. This number is the one indicated in the corresponding cell of the heatmap. Note that genes g1 and g2 form a possible reciprocal gene to gene connection if and only if there is a RACE primer in g1 pointing in the direction of g2 and a RACE primer in g2 pointing in the direction of g1.
(PNG)

Figure S12 **Number of observed (left) and of expected (right) gene to gene connections on chromosomes 21 (top) and 22 (bottom).** The shape of the observed distributions is similar for the two chromosomes, as well as the shape of the expected ones, however the distributions are decreasing much more rapidly for the expected connections compared to the observed connections.
(PNG)

Figure S13 **Difference between number of observed and number of expected gene to gene connections on chromosome 21 (A) and on chromosome 22 (B).** These two histograms (A and B) represent the distributions of the difference between the number of observed and the number of expected gene to gene connections for reciprocally connected genes on chromosomes 21 and 22 respectively. These distributions are shifted towards the positive values, and have a mean of 6 and 5 respectively. In our analysis the difference between the number of observed and the number of expected gene to gene connections of reciprocally connected genes is used as a score for those genes and is used to delineate a set of genes much more connected than we would expect given their length and number of primers: the hubs.
(PNG)

Figure S14 **Different categories of genes used in the RACEarray experiments.** Proportional Venn diagram representation of inclusion relationships between some of the most used sets of genes used in this study. The area highlighted in light blue corresponds to non-hub genes, which are all reciprocally connected.
(PNG)

Figure S15 **Expected number of gene to gene connections found by RACEarray and RNA PET ditags in K562.**
(JPG)

Figure S16 **Expected number of gene to gene connections found by RACEarray and Illumina Human Body Map PE50 RNAseq (A) in Testes+Prostate and in Brain (B).**
(JPG)

Figure S17 **Interspecies chimeric RNAs used as a metric of technical artifacts.** The number of reads/10 M total reads for intra-genomic and inter-genomic chimeric junction sites is plotted human and fly alone and various ratios of RNAs from human and fly (mixtures). A total of at least 25 nucleotides on each side of a chimeric junction site was chosen as a minimum to allow for unique mapping in each genome.
(JPG)

Figure S18 **5C data validates RACEarray gene to gene connections.** This figure represents the distribution of the proportion of gene to gene connections validated by 5C in 1,000 sets of gene to gene connections detectable by RACEarray and by 5C with the same distributions of distance between connected genes and of length of connected genes as in the 638 connections detectable by both techniques that are actually observed. The mean of this distribution is 70.8 (standard deviation = 1.9), which is significantly lower than the observed proportion (496/638 = 77.7%, depicted by the arrow on the right, p-value<10−3).
(JPG)

Figure S19 **Domain organization for chimera OTTHUMP00000221101.** Chimera OTTHUMP00000221101 results from the fusion of two receptors involved in immune response, Interferon-alpha/beta receptor 2 (IFNAR, N-terminal section) and Interleukin-10 receptor subunit beta (IL10RB, C-terminal section). The resulting protein will have an extra-cellular domain that is double the size of the usual extra-cellular receptor domain and that is composed of a repeat of paired tissue factor (green) and alpha/beta interferon receptor (red) domains. The chimeric protein also conserves a signal peptide signal and a single trams-membrane helix. A similar domain configuration is recorded in Uniprot for the chicken interferon receptor (Q5XPI1_CHICK).
(PDF)

Figure S20 **Model of possible structure of fused fragments for chimera OTTHUMP00000221101.** Models for the N- and C-terminal sections have been obtained respectively from structures 2hym and 3g9v by comparative modeling (Modeller, http://salilab.org/modeller). Linker region (shown as a gap in the structure) is located in flexible regions for both templates. Domain folds could then be maintained independently.
(PNG)

Table S1 **Number of gene to gene and number of reciprocal gene to gene connections by distance.** This table is similar to Figure 5B and provides numbers of gene to gene and of reciprocal gene to gene connections detected in each cell type, split by distance bins: - <150 kb -150 kb – 1 Mb -1 Mb – 5 Mb ->5 Mb. This table shows that (1) the number of connections is similar in cell lines and tissues, (2) the distribution of connections in distance classes changes if we consider all or only reciprocal connections, (3) between one third and half of the reciprocal connections are cell-type specific and (4) all the figures are quite high meaning that chimeras are far from being exceptional.
(JPG)

Table S2 **Validation results of chimeric transcripts by RNase protection assays.** This table lists the RACE name, the pool of poly-A+RNA used, description of the probes, a summary of the RNase Protection Assay screening with a detailed interpretation of the results based on the autoradiography gel.
(XLSX)

Table S3 **Names and characteristics of the hubs.** For each of the 74 hubs the table provides: - the number of observed connections, - the number of expected connections, - the difference between the two, which could be seen as their connectivity score.
(JPG)

Table S4 **Hubs have higher phylogenetic depth than non hubs.** We consider four different gene sets (see Figure S14 for a description): - RACE interrogated (“raced”) genes - reciprocally connected genes – hubs - non hubs and for each of them, we provide the number of genes with an Ensembl gene ID, and the number and the proportion of this total, that has an ortholog in the 6 following species, as found using biomart on ensembl51: • Yeast • *C. elegans* • *Drosophila* • *Tetraodon* • Chicken • Mouse. For each of these species we then provide the Fisher exact test p-value obtained while testing the following hypothesis: “Being a hub is independent on having an ortholog in a given species”. A star above this number on the table means the p-value is significant (less than 0.01). Note that both the proportions of genes with an ortholog in each of the 6 species for RACE interrogated (“raced”) genes, hubs and non hubs, and the significance of the Fisher tests mentioned here are provided on Figure 9B.
(JPG)

Table S5 **Over-representation of cliques in chromosomes 21 and 22.** For each chromosome, we report the number of cliques observed, as well as the mean and the standard deviation of the number of cliques expected.
(JPG)

Table S6 **Constitutive cliques.** For each constitutive clique (the maximum size is 3), we provide: • the names of the genes involved in the clique, • the chromosome where the clique is observed, • the list of cell types in which the clique is observed.
(JPG)

Table S7 **Overlap of maximal cliques.** For each clique size, we report: - the number of cliques observed, - the number of corresponding edges if cliques were not overlapping, - the number of observed edges.
(JPG)
