# Supplementary material for: Evidence for Transcript Networks Composed of Chimeric RNAs in Human Cells
Source: PLoS One. 2012 Jan 4;7(1):e28213. doi: 10.1371/journal.pone.0028213 (PMC3251577; doi:10.1371/journal.pone.0028213)
Supplement: Materials S1 — Supplementary text. (DOC) [file pone.0028213.s001.doc]

**SUPPLEMENTARY MATERIALS**

1. **Pooling of RACE reactions.**
2. Method.

Ideally, a distinct RACE reaction is realized per primer, and the corresponding products are hybridized on a single tiling array. In practice, this is not feasible for large scale projects. Indeed, with 16 cell types and 1,668 primers (up to 3 index exons per gene and 2 types of RACE: 5' and 3'), about 25,000 tiling array hybridizations would then be required in our experiment.
Therefore, results of some RACE experiments are pooled together and put on a same array. In order to simplify the downstream data analysis, it is desirable to maximize the genomic distance between the primed regions of the RACE experiments that are pooled together, so that each resulting signal from the array can be assigned to its corresponding primer as unambiguously as possible. The composition of each pool in terms of primers has to be carefully designed, with the aim of simultaneously achieving two goals:
1. Minimizing the number of pools (and consequently the cost of the experiment),
2. Maximizing the genomic distance between the genomic positions of the primers (defined as the coordinates of the corresponding ACEP) that are pooled together.
In order to address this optimization problem, the pooling strategy is organized in two steps:
1. Given the genomic positions of the primers, compute the minimum number of pools required in order to guarantee a minimum distance between two consecutive primers from the same pool. The longer the distance, the more the pools.
2. Given this total number of pools, distribute the primers within pools, in order to maximize the distance between each two consecutive primers.

These two steps are described in more details below.
Given a minimum distance *d* required between two consecutive primers from the same pool, the minimum number of pools *N* needed to achieve this condition is given by the *ComputePoolNumber* function. The general idea of this function is to stack as many primers as possible in the first pools (ideally only one), taking into account the minimum distance. The creation of another pool is required when a primer does not fit in the previously created pool(s). The computational complexity of the algorithm is linear in time and in memory. After this first step, the minimum number of pools is known, but the primers are not homogeneously distributed across the pools: the first pool contains the most primers, and the last one the least. The *DistributePrimers* function is designed to redistribute the primers evenly, so that the average distance between consecutive primers from the same pool is as high as possible. Going along the list of primers ordered by genomic position, the idea is to distribute them into the pools sequentially, a process resembling that of distributing cards to players. This way two properties are guaranteed (proof not shown):
1. the minimum distance between two consecutive primers from the same pool is not lower than *d*;
2. the average distance between two consecutive primers from the same pool is as high as possible.

The scripts *ComputePoolNumber* and *DistributePrimers* are available upon request.

1. Results on chromosomes 21 and 22.

In this study, two chromosomes are interrogated: chromosomes 21 and 22. The *ComputePoolNumber* function is applied independently on each one, and the largest of the two *N* values output by this function is then provided as input to the second *DistributePrimers* function, which is also independently applied to each chromosome. Note that primers from different chromosomes are pooled together.

In fact, a slight modification of the algorithms has been made in order to take into account the orientation (strand) when considering the minimum distance. Indeed, as primers should only allow amplification from their 3' ends, the minimum distance from their 5' end can be lower than from the 3' end (no amplification is supposed to originate in this direction, which reduces the assignment issue of the RACEfrags signals located in this area). Two minimum distances can thus be defined: distance *L* in 3' of the primers, and distance *l* in 5' of the primers. In practice, primers boundaries are initially extended accordingly (*i.e.*, by *L* base pairs in 3' and by *l* base pairs in 5') before being input to the pooling process.
The final values resulting from the pooling process are:

- *L*=minimum distance from 3': 900,000 bp
- *l*=minimum distance from 5': 90,000 bp
- number of pools: 102

For these final values, figure S1 provides the distance distribution between consecutive pooled primers according to their relative position and chromosome.

1. **RACEfrag calling.**

RACEfrags are usually called from Affymetrix tiling array probe intensities using TAS (http://www.affymetrix.com/support/developer/downloads/TilingArrayTools/index.affx). In order to address the issues of pool-unspecific RACEfrag (namely, RACEfrags that repeatedly appear in many different unrelated experiments) and unspecific probes (we observed that 7% of the probes in chromosome 21-22 tiling arrays had multiple exact matches in the genome), we discarded array signals coming from these two types of unspecific probes and optimized the parameters of the RACEfrag calling, using a RACEfrag caller developed in-house (software available upon request). Similarly to TAS, we used four different parameters for calling RACEfrags from probes:

(1) the percentile intensity threshold *I* above which we consider a probe to produce a positive signal. Given this threshold, the set of positive probes can be defined for a given experiment.

(2) the maximum number *M* (*Maxgap*) of nucleotides that is allowed between two consecutive positive probes to be included in the same RACEfrag.

(3) the minimum number *m* (*minrun*) of probes that are needed to call a RACEfrag.

(4) the calling mode *c* of the RACEfrag, relatively to the genomic coordinates of the probes. This parameter can take only one of the two following values: "middle-middle" when RACEfrags are called from the middle of the first probe to the middle of the last probe, and "start-stop" when RACEfrags are called from the start of the first probe to the end of the last probe.

The optimization of RACEfrag calling was run on a random subset of 10 array experiments, with all possible combinations among the following values of the four calling parameters:

- I between 98.1%ile and 99.9%ile with 0.2 steps iterations (10 values),
- M values of 25, 42, 59, 76, 93 bp (5 values),
- m values of 3, 4, 5 probes (3 values),
- c values middle-middle and start-stop (2 values).

To evaluate these 300 sets of parameters, the resulting RACEfrags were compared with annotated exons using 2 different measures: (1) the exonic accuracy (see top of figure S2), computed for RACEfrags that overlap internal exons, represents the proportion of predicted and annotated nucleotides that are overlapping. This measure is computed on RACEfrags of a given experiment and projected internal exons. (2) The RACEfrag acceptor and median donor scores (see bottom of figure S2) is computed by considering the scores of the potential splice sites around the RACEfrag boundaries as predicted by the *geneid* software[7]. For fixed values of I, M and m, these two measures always gave better results for c=start-stop than for c=middle-middle, so the former only was considered for the rest of the study. For a given m value, as an exonic accuracy value can be computed for each combination of M and I value, a three dimensional space was used to represent the results, as shown in figure S3. Note that, in general, the RACEfrag acceptor and donor scores were better for higher intensity thresholds. From these initial results, another round of optimization was performed, including this time the whole set of 1,020 experiments. Parameters c and m were fixed respectively to c=start-stop and m=3, and the following values were assessed for the combination of (I,M): (99.7,76), (99.7,59), (99.7,42), (99.7,25), (99.35,25), (99.0,25), (98.5,25), and (98.0,25). The (I,M) value giving the best exonic accuracy and splice site scores was (99.7,76), therefore resulting in the final optimal set of parameters:

- I=99.7%ile,
- M=76bp,
- m=3 probes,
- c=start-stop.

1. **“*In Silico*” RACEfrag filtering.**

In order to eliminate RACEfrags coming from unspecific priming and/or unspecific cross-hybridization, we used our in-house *in silico* RACEarray simulator [5]. This simulator starts from a known set of transcripts, primers and array probes, and generates simulated RACEfrag maps from which we can confidently discriminate between *bona fide* RACEfrags (i.e., originating from specific priming and specific array hybridization) and artefactual ones (i.e., arising from RACE mis-priming and/or array cross-hybridization). More precisely this simulation involves two steps (see figure S4):

1. *in silico* RACE , which consists in searching each RACE primer against the transcriptome (we use RefSeq RNAs + Unigene + Gencode transcripts). A primer is considered matching an mRNA if it aligns to it with >95% ID over >60% of its sequence, and if the alignment includes one of the 4 3'-most nucleotides of the primer. When a match is found, the primer is elongated until the end of the transcript, in either the 5' or the 3' direction. At this point, and for each primer, we end up with a population of RACE products, arising from both specific and unspecific priming.
2. *in silico* array hybridization , which consists in scanning the two strands of all these RACE products with all probes of the tiling array. We can allow for 0 or more mismatches for a probe to be considered positive. Since our starting material consists in known transcripts that have been manually mapped to the genome, we do not expect any of the RACE products obtained at step 1 to highlight probes outside of the annotated exons of the target locus; if they do, these highlighted probes will be considered unspecific. The result of this step is a set of Simulated Positive Probes (SPPs), that we can split into the following categories:
3. *Bona fide* SPPs : These are SPPs that overlap the annotated target locus exons.
4. Unspecific SPPs (USPP s): These are SPPs that map outside of the target locus exons. In our model, they clearly correspond to false positives, originating from RACE mis-priming and/or array cross-hybridization.

Thus, any RACEfrag overlapping USPPs present in its pool, is eliminated (*ibid*, supplementary section 1.2.2). When used with Unigene and RefSeq transcripts, our RACEarray simulator filter eliminates 3,669 RACEfrags out of our initial 306,368 (1%).

1. **RACEarray chimeras are also found by RNAseq experiments.**
   1. Comparing RACEarray to PET RNA ditag.

We compared the chimeras found by RACEarray in K562 to the ones found by GIS RNA PET ditag performed on K562 cytosolic poly-A+ RNA in the context of the ENCODE project (hg18 mapping downloaded from the UCSC). More precisely whenever the 5’ and the 3’ tags of a given PET ditag map to two different genes on chromosomes 21 and 22, this pair is recorded. Doing so, a total of 412 different gene pairs were identified by the RNA PET ditag experiment. On the other hand, a total of 4,656 directed gene to gene connections were found by RACEarray in K562 (Figure 5B, direction is from primer to RACEfrag), corresponding to a total of 4,392 gene pairs. The comparison of the gene pairs provided by the two techniques yielded a total number of 56 gene pairs common to both techniques, a number that is much higher than expected by chance as shown by independence test (Figure S15, p-value < 10^-4^).

- 1. Comparing RACEarray to Paired end RNAseq.

We also compared the chimeras found by RACEarray in Testes and Prostate and in the Brain tissues, to the chimeras found by the Illumina Human Body map Paired End 50nt RNAseq experiments performed in Testes and Prostate and in Brain respectively. Using the same protocol as explained above, we found a total of 4,272 and 7,448 gene pairs detected by RACEarray in Testes+Prostate and in the Brain tissues respectively, and a total of 2,013 and 1,066 gene pairs detected by RNAseq in Testes+Prostate and in Brain respectively. The comparison of the gene pairs found by both techniques in each tissue revealed a total of 150 and of 163 common gene pairs respectively, which were both much higher than expected by chance (Figure S16, p-value < 10^-4^).

1. **Network analysis.**
   1. Circos RACEarray maps.

Individual RACEfrag connections from each cell type are represented in figures S6 (chromosome 21) and S7 (chromosome 22). See also legend of Figure 4 in the main article.

- 1. Over-representation of reciprocal gene to gene connections.

For each chromosome, let us consider the directed graph made from the external genic RACEfrags observed in this chromosome, *i.e.,* the graph where each directed edge represents a directed connection between two genes: the gene where a primer lies and the gene comprising an external genic RACEfrag assigned to this primer (note that when a directed edge is present several times, it is only considered once). For chromosome 21 the number of edges in this graph is 5,153, and for chromosome 22 it is 11,512. The number of reciprocal gene to gene connections for each chromosome is then obtained by counting the pairs of vertices connected by two directed edges going in opposite directions in such a graph: we count 792 reciprocal gene to gene connections for chromosome 21, and 1,532 for chromosome 22. In order to determine if these numbers are more than expected by chance, we used simulations. We first generated random graphs that preserve the degree sequence (numbers of edges per vertex) of the original network. We then counted the number of reciprocal connections in all randomized graphs which gave us an empirical distribution of the count, from which we estimated the mean and the standard deviation of the count. Both the generation of graphs and the count of reciprocal connections were carried out using the program mfinder [16].

We generated 10,000 random graphs for each chromosome, and found 508±15 reciprocal connections for chromosome 21,527±19 for chromosome 22 (*i.e.*, 1.6 fold less than observed for chromosome 21, and 2.9 fold less than observed for chromosome 22). From now on, we restrict the network analysis to the network of reciprocally connected genes, depicted on figure S8.

- 1. Concordance of reciprocal gene to gene connections across cell types

The pairwise Pearson's product-moment correlations between all possible cell type pairs is computed based on the number of reciprocal gene to gene connections commonly observed between them. This is computed in the universe of all gene pairs that could potentially be reciprocally connected given their primer configuration (i.e., there exists a primer in the first gene located on the same chromosome and pointing in the direction of the second one, and a primer in the second gene pointing in the direction of the first one). Contingency tables are computed for each cell type pair, and the corresponding Pearson's product moment correlations are displayed in figure S11 (all p-values less than 2.2*10^-16^) together with a heatmap.

- 1. Degree distribution and hubs.

i. Characterization of the degree distribution and definition of hubs.
We found that the degree distributions of the transcriptional networks of both chromosome 21 and 22 clearly deviate from a Poisson distribution (goodness of fit chi2 test, p=1.4*10^-70^, p=3.4*10^-230^), which is the type of distribution that would be expected from an Erdos-Renyi random graph (a random graph model where all connections have the same probability). As many other biological networks, the degree distribution of our network exhibits a broad tail, that is, many nodes have a low degree while few nodes have a high degree [17]. We further find that the degree of a node is positively correlated with the length of the gene and the number of primers used for the RACE reactions. The longer a gene, the more connections it may be involved in. The more primers designed in a gene, the more connections it may be involved in. Hence, focusing on genes with a high degree would merely capture these two effects. Next, we concentrate on genes that are more connected than expected given their length and number of primers.

More precisely the probability of the existence of a reciprocal connection between genes A and B, should be proportional to: (1) the number of primers of gene A, (2) the number of primers of gene B, (3) the cDNA length of gene A, (4), the cDNA length of gene B. Approximating the cDNA length of a gene by its cumulative projected exon length, and calling pr(G) and lg(G) the number of primers and the length of a gene G respectively, since we want to have the following property satisfied:

the probability of observing a reciprocal connection between gene A and gene B is given by the following formula:

And the expected number of reciprocal connections of gene G is thus given by this formula:

.

Figures S12 displays the number of observed and of expected reciprocal gene to gene connections for chromosomes 21 and 22.

Next, we concentrate on genes that are much more connected than expected given their length and number of primers, and call them hubs. A hub is a gene in the top 15%ile of the distribution of the difference between the number of observed and the number of expected reciprocal connections (figure S13), which qualifies 74 genes as hubs (see characteristics of these genes in table S3). Also, as we are working on the network of reciprocally connected genes, we define a reciprocally connected gene as non hub if its ranking falls below 15%ile. Figure S14 shows a Venn diagram explaining this categorization of genes and connections.

ii. Hubs are more highly expressed than non hubs.

We computed the expression of a gene from tiling array experiments performed on the same 16 different cell lines and tissues as the RACE experiments have been performed. More precisely the expression of a gene in a tissue is computed as the average expression of its projected coding exons. The expression of an exon is computed as the average over all the intensities of the tiling array probes overlapping the exon. Finally, the general expression of a gene is computed as the average of its expression over the 16 different cell lines and tissues (data provided at <ftp://genome.crg.es/pub/Encode/ChimericRNAChr2122/suppl_materials_gene_expression_profiles.zip>). The distribution of the expression of the 74 hubs and of the 362 non hubs, is given on figure 9A.

iii. Hubs do not have higher similarity to their connected genes compared to non hubs.

If the nucleotide sequence of a given transcript T (from gene G) is very similar to that of another transcript T' (from gene G'), then RACE products originating from a primer of transcript T will cross-hybridize and generate artefactual RACEfrags within transcript T', thus forming an artefactual connection between G and G'. To check whether the formation of hubs was due to such a cross-hybridization, for each experiment (*i.e.*, combination of pool/tissue) we performed an all-against-all, per-chromosome (as our assignment method does not allow for inter-chromosomal connections) BLAST search of RACEfrag sequences (with BLASTN default parameters). RACEfrags exhibiting significant nucleotide similarity (i.e., at least one HSP of more than 50 nucleotides) were flagged as Potentially Cross-Hybridized RACEfrags (PCHR). We then computed the respective proportion of PCHR in three different sets: (i) all RACEfrags, (ii) RACEfrags involved in a non hub connection, (iii) RACEfrags involved in a hub connection. There was no enrichment of PCHR in hubs compared to non-hubs (0.44% vs. 0.51%), nor were PCHR over-represented in these two sets when compared to all RACEfrags, where this proportion was 0.5%. Hub genes are therefore not more likely to be due to cross-hybridization events than other genes, nor do reciprocal gene connections seem to arise from cross-hybridized RACE products.

iv. Hubs have higher phylogenetic depth than non hubs.

In order to test the hypothesis : “Being a hub is independent on having an ortholog in a given species”, we selected 6 different species representing a large panel of evolutionary distances from human: *Saccharomyces cerevisiae,* *Caenorhabditis elegans*, *Drosophila melanogaster*, *Tetraodon nigroviridis*, *Gallus gallus* and *Mus musculus.* Using *biomart* (http://www.biomart.org/) we interrogated the ensembl51 database to determine for each RACE interrogated gene whether it had an ortholog in each of the 6 species. We then computed the number and the proportion of genes with an ortholog in each of the species for four different gene categories: RACE interrogated genes, reciprocally connected genes, hubs and non hubs. For each species, we have at hand a 2*2 contingency table indicating whether human genes are hubs or non hubs and if they do or do not have an ortholog in this species. We test our hypothesis using a Fisher exact test, and we report the results in Table S4. As one can see, p-values are significant for the 3 most distant species (yeast, C. *elegans* and drosophila), but not for the 3 less distant ones (*Tetraodon*, chicken and mouse). This is likely to be due to the fact that the vast majority of our initial RACE interrogated genes have an ortholog in each of these 3 species, making it difficult to differentiate hubs and non hubs with respect to this criterion.

In order to see to which extent the overall high expression of hubs (see paragraph ii. of the present subsection), could explain their overall higher phylogenetic depth over the non-hubs, we did the same test as before but instead of comparing the 74 hubs to the whole set of 362 non-hubs, we compared the 74 hubs to 74 randomly picked non-hubs with the same expression distribution as the 74 hubs. More precisely we made 4 expression classes based on the expression levels of hubs:

- Expression < 284,
- 284 ≤ Expression < 536,
- 536 ≤ Expression < 896,
- Expression ≥ 896,

and then randomly picked the same number of non-hubs in these 4 classes as were present in the set of 74 hubs, thus resulting in a set of 74 non-hubs with the same expression distribution as the 74 hubs. We then redid the same 6 fisher-tests associated to the 6 species as described above for the comparison of the 74 hubs against these 74 non-hubs, and found the following:

- Hubs have more orthologs than non-hubs in each of the 6 species,
- The difference between hubs and non-hubs is significant for yeast.

This shows that the overall high expression of hubs cannot totally account for the higher phylogenetic depth of hubs with respect to other reciprocally connected genes.

v. Hubs are depleted in repeat sequences compared to other connected genes.

In order to see whether hubs are enriched in repeat sequences compared to other connected genes, we made use of the RT-PCR confirmed cases presented in the previous section (8). There is at least one RT-PCR product emerging from 34 hub genes, out of the 74, and there are 77 RT-PCR clones involved in these 34 hub connections. Then we found that:

- 24 of the 77 RT-PCR products involved in hubs contain repeats (30%),
- 58 of the 131 RT-PCR products not involved in hubs contain repeats (44%).

It thus seems that hub connections are depleted in repeat sequences compared to other connected genes.

- 1. Clique analysis.

i. Definition of cliques.

In order to determine if the gene to gene reciprocal network was locally structured, we investigated if it was enriched in any network motif. A network motif has been defined as a pattern of interconnection occurring in a network at numbers that are significantly higher than those in randomized networks [16]. Using the software mfinder with default parameters, we found that our network was enriched in a specific type of motif: cliques (when looking for motifs of size 3 in our networks, we found 3,594 cliques and 23,505 pseudo-cliques, while expecting 1,119±43 and 30,930±130 respectively).
A clique is a set of nodes which are all pairwise connected. By definition, a clique of size k contains k cliques of size k-1. However, not all cliques of size k-1 are included in cliques of size k. We can therefore define two types of cliques: maximal and non maximal. A clique of size k is maximal if it is not included in a larger clique. It is non maximal otherwise.

Non maximal cliques correspond to partial descriptions of clusters of genes, in the sense that they can be extended. In this work, since we are interested in the size of the clusters, we focus on maximal cliques. Whenever the term clique is used in the paper, it implicitly refers to maximal cliques.

Maximal cliques were identified in the reciprocal gene to gene network using the R package (*igraph*) [18].

ii. Over-representation of cliques.

We assess the over-representation of cliques using simulations. In order to determine if the number of maximal cliques that we had found was significantly different from the numbers expected by chance, we performed simulations, that is, we also enumerated maximal cliques in 10,000 randomized networks. Randomized networks were generated using the function *degree.sequence.game* of the R package “igraph”, so that they have the same degree sequence as in the original network. We tested both the “stubs” method and the Vigier-Latapy algorithm for generating networks with prescribed degree sequence (the “switch” method in not implemented in igraph)). Both methods guarantee uniform sampling of networks. The stubs method may generate non simple networks (i.e. containing multiedges and/or loops), whereas the Vigier-Latapy algorithm generates only connected networks. In both cases, we found very similar values for the mean and the variance of the number of expected maximal cliques. For instance, the expected number of maximal cliques of size 3 was found to be 40.05±18.02 using the stubs methods whereas it was found to be 40.24±20.15 using the Vigier-Latapy algorithm.
The two networks corresponding to chromosomes 21 and 22 were considered independently. The results of the two series of simulations were then merged to produce the final results for the combination of both chromosomes. Table S5 summarizes the results for each chromosome.

iii. Constitutive cliques.

We define a constitutive clique as a clique observed in at least 2 cell types. We found 11 size 3 constitutive cliques present in 2 cell types, and one size 3 constitutive cliques present in 3 cell types. Names of the genes involved in these cliques as well as chromosome and cell types where these cliques appear, are indicated in table S6.

1. **Network structure and coordination of expression.**

Expression levels for each gene for each tissue were obtained as described in section 5.e.ii. For each gene, we have an expression profile, that is, a collection of 16 expression levels, 1 for each tissue. For each gene pair, we compute the correlation coefficient of their expression profiles. This is our measure of co-expression CE. Finally, for each gene pair, we compute the genomic distance GD as the distance between their centers, irrespective of the strand. The center of a gene is the average position between its boundaries.
For each gene pair, we compute its clique size CS as the size of the largest maximal clique it belongs to.

If all cliques were non overlapping, the number of connections participating in n cliques of size k would be n*k*(k-1)/2. Due to the overlap between maximal cliques, the number of edges participating in cliques of size k is lower, as indicated in table S7.

**Connected genes are co-expressed:**
In order to establish the relation between co-expression and connectivity, we tested the following models. Let CN be a variable indicating whether two genes are connected. For all models, the error is assumed to be normally distributed and the connections are assumed to be independent. We considered the following models:

M_0_: CE_i_ = a + e_i_,

M_1_: CE_i_ = a + b*CN_i_ + e_i_,

M_2_: CE_i_ = a + b*GD_i_+ e_i_,

M_3_: CE_i_ = a + b*GD_i_ + c*CN_i_+ e_i_.

We compared models using analysis of covariance ANCOVA and showed that co-expression was related to connectivity (M_1_ Vs M_0_, p=4.724*10^-42^), co-expression was related to genomic distance (M_2_ Vs M_0_, p=2.757*10^-6^). We further showed that these effects were additive and genomic distance was not a confounding factor (M_3_ Vs M_2_, p=1;244*10^-40^). Overall, connected genes have a higher correlation of expression profile than non connected genes, and this is true even when taking into account the fact that connected genes are closer on the genome.

**The larger the clique, the more correlated the expression profiles:**
Within the set of connected genes, we found that genes within cliques had a higher correlation of expression profile than genes not in cliques (*t* test, p=4.8*10^-3^). We further examined if large cliques contained genes with higher coordination of expression than smaller cliques. We tested several thresholds to define large cliques. Performing t tests, we found no difference between large and small cliques when the threshold was set to 6 (p=0.119) or 7 (p=0.175). On the other hand, we did find a difference between large and small cliques when the threshold was set to 3 (p=4.8*10^-3^), 4 (p=7.3*10^-6^) or 5 (p=7.1*10^-3^).

We further showed that there was a linear dependence between co-expression and clique size. We found that the Pearson correlation coefficient was R=0.12 which was significantly different from 0 (p=4.14*10^-9^).

Finally, in order to establish the general relation between co-expression and clique size, controlling for the effect of genomic distance, we considered the following models:
M_4_: CE_i_ = a + b*GD_i_ + e_i_,

M_5_: CE_i_ = a + b*GD_i_ + c*CS_i_+ e_i_,

We compared models using ANCOVA and showed that there was a linear dependence between co-expression and clique size, and genomic distance was not a confounding factor (M_5_ Vs M_4_, p=2*10^-6^).

The expression levels of each gene of chromosome 21 and 22 in 16 different tissues and cell lines are provided at:

<ftp://genome.crg.es/pub/Encode/ChimericRNAChr2122/suppl_materials_gene_expression_profiles.zip>

1. **Protein-coding potential of the RT-PCR validated chimeric RNAs**

Each of the 208 RT-PCR clusters considered positive (see section 8-d-(i)) was submitted to an *in silico* translation in all six possible reading frames. Only those exhibiting a full-length CDS (*i.e.*, with no stop codon, but without any requirement for a start codon) in at least one of the six frames were counted as coding. 87 of them (42%) fell in this category. Surprisingly, a higher proportion of non-canonically spliced clusters were coding (64 / 133, *i.e.* 48%), when compared to those splicing canonically (23 / 75, *i.e.* 31%).

In order to determine if these proportions were significantly higher than one would expect by chance, we created and translated a shuffled set of sequences using the following procedure, *in silico*. First, each of 208 RT-PCR sequences was randomly paired with another sequence from the same set. A hybrid RT-PCR sequence was then generated, using the original chimeric split points (namely, the exon junction from which the existence of the chimeric RNA was deduced) of both sequences, thus consisting of a concatenation of sequence #1, from its start to its split point, and sequence #2, from its split point to its end. The shuffling procedure, applied to each 208 sequences and repeated 1,000 times, produced a set of 208,000 hybrid sequences with a length distribution highly similar to the real one (mean: 581 nucleotides (real set) vs. 583 (random set)). This realistic sequence length distribution prevents us from underestimating the coding potential of the shuffled sequences. Applying the aforementioned translation procedure to the 208,000 shuffled sequences revealed that 63,696 of them (31%) bore a full-length CDS, a significantly lower fraction than the observed 42% (p-value=0.01).

We derived the RT-PCR sequences’ coding statistics described above to compute the proportion of coding, RT-PCR-confirmed RACE connections. To do so, we counted how many of the 112 confirmed chimeric connections (see section 8-d-(i)) were supported by at least one of the 208 underlying RT-PCR products detected as coding in the previous step. 53 of them (47.3%) did, highlighting their high coding potential.

1. **Structural and functional analyses of the potentially coding RT-PCR validated chimeric RNAs**

In order to investigate the potential protein structure and function of the RT-PCR validated chimeric transcripts, we focused on a subset of 61 potentially coding transcripts that (1) were revealed by reciprocal gene to gene connections, and (2), maintained the annotated CDS sequence and frame of the 5' (i.e., N-terminal) gene.

More precisely the characterization of the 61 coding chimeric transcripts included:

- Domain characterization,
- Binding site mapping,
- Structure modeling,
- Trans-membrane segment and signal peptide prediction.

Based on the manual analysis of this information we have characterized 5 cases previously identified in databases, 4 cases in which the functions of the two protein fragments product of trans-splicing will most likely produce a protein with a new dual combined function, 7 cases where the fusion of the two proteins will likely lead to a change in cellular localization and 21 cases in which the new proteins produced by trans-splicing will contain incomplete fragments that most likely will lead to a non-functional peptide. This analysis points to at least three cases of potential biological relevance that will be most interesting for a detailed experimental validation (see figures S19 and S20).

The RT-PCR primer sequences as well as the positive RT-PCR products are provided at the following address:

<ftp://genome.crg.es/pub/Encode/ChimericRNAChr2122/suppl_materials_RT-PCR_sequences.zip>

**Figure legends**

**Figure S1: Distance between consecutive pooled primers.**

This figure represents the distribution of distances between consecutive primers of the same pool for each chromosome and each relative position of primer pairs: head to head (→ ←), head to tail (→ →) and tail to tail (← →), as a box plot. The gene density of chromosome 22 being higher than that of chromosome 21, the distance between consecutive pooled primers is smaller for the latter than for the former. Also, as expected, the distance between consecutive pooled primers is higher for head to head than for head to tail configuration, and higher for head to tail than for tail to tail configuration.

**Figure S2: Two measures to assess RACEfrag sets.**

This figure describes the two measures used to assess RACEfrag sets while optimizing the parameters used for RACEfrag calling: the exonic accuracy and the splice site score. The exonic accuracy assesses the RACEfrag set with respect to a reference set: the projected internal exons. More precisely for each projected internal exon, considered as a reference, the exonic accuracy assesses the accuracy with which the RACEfrags overlapping this reference mimics this reference. This accuracy is measured in terms of intersection over union of the projected internal exons and the RACEfrags, i.e., for each projected internal exon with overlapping RACEfrags, the number of nucleotides in common between the two sets is divided by the number of nucleotides in either of the two sets. The exonic accuracy of a RACEfrag set is then the median of the exonic accuracy of projected internal exons with overlapping RACEfrags. Unlike the exonic accuracy the splice site score of a RACEfrag set does not depend on any reference but is rather intrinsic to the RACEfrag set. More precisely, the spice site score is divided into two sub-measures: the acceptor score and the donor score. Both scores involve the scanning of two windows around the RACEfrag boundaries, W1 around the left boundary and W2 around the right boundary, where both acceptor and donor sites have previously been found by the geneid program. The RACEfrag acceptor score is then defined as the score of the best acceptor site on the 2 windows, and the RACEfrag donor score as the one of the best donor site on the 2 windows.

**Figure S3: RACEfrag calling.**

This figure represents the exonic accuracy of 10 RACEfrag sets coming from 10 randomly chosen experiments, as a function of the intensity threshold (I) and the maxgap (M), for 3 different minrun values (m): 3, 4 and 5. The blue arrows indicate the maximum exonic accuracy found over all the possible values of the three parameters, and the red arrows the minimum exonic accuracy. The maximum is reached for I=99.1%ile, M=59 bp, m=5 probes.

**Figure S4: USPP filter.**

This simulation involves two steps: (1) RACE from a set of primers and a set of known transcripts; (2) hybridization of the obtained RACE products on tiling arrays. The RACEarray simulator generates a set of tiling array probes that are highlighted by the RACE products and that we call simulated positive probes (SPPs). These SPPs can be further divided into two categories: (1) bona fide SPPs, i.e. overlapping an exon of the target locus; (2) unspecific SPPs, also called USPPs, i.e. mapping outside of the target locus exons. In our model these USPPs correspond to false positives that originate from RACE mis-priming and/or from array cross-hybridization (see text for a more detailed explanation).

**Figure S5: RACEfrag assignment.**

This figure is divided into three parts: 1) on the top, the annotations of a given chromosome are represented, which are here the different alternative transcripts of three loci: A, B and C; 2) in the middle, the primers and RACEfrags of three different pools in several tissues are represented; 3) on the bottom, the formula of the assignment confidence score is provided again as well as its application on 5 different (RACEfrag, locus) pairs (note that here two RACEfrags with the same coordinates are given the same identifier). The first two parts of the figure are thus dedicated to the description of the assignment method, while the third part shows how the assignment score behaves on already assigned RACEfrags associated to their locus. Primers are named and colored after the locus they are originating from, and RACEfrags after the locus they have been assigned to. In pool 1, primer C1 is active and points in the direction of all RACEfrags, so all RACEfrags of pool 1 are assigned to primer C1. In pool 2, it is the same with primer C2, and in pool 3 the same with primer A1. Then the ACS formula is applied to 5 different (RACEfrag, locus) pairs, and the lower the score the more confidence we have in the assignment of the RACEfrag to the locus. Here the (RACEfrag, locus) pair we are the most confident in is (3,C) since RACEfrag 3 appears 4 times in total and each time it appears it is assigned to locus C. Also, the fact that it is assigned to two different primers of locus C, primers C1 and C2, strengthens the confidence we have in this pair. The pair (4,C) is similar to the pair (3,C) except that RACEfrag 4 appears in 2 experiments instead of 4. It thus also has a good score, although less than the one of (3,C). The pair (2,C) is like the pair (3,C) except that RACEfrag 2 also appears in pool 3 , tissue 1 where it is assigned to locus A. This makes it more uncertain we should assign RACEfrags 2 to locus C, as compared to RACEfrag 3, and this is why the score of (2,C) is lower than the one of (3,C). The pair (5,C) is similar to the pair (4,C) except that RACEfrag 5 is only assigned to 1 primer of locus C (primer C1), compared to two primers of locus C for RACEfrag 4 (primers C1 and C2). This explains the lower score of (5,C) with respect to the one of (4,C). Finally the pair (2,A) is given a very bad score since RACEfrag 2 appears 5 times but is assigned only once to locus A.

**Figure S6: Chromosome 21 transcriptional networks.**

RACE connection networks in all 10 assayed cell types are represented. In each plot, the chromosome is depicted as a circle, and RACEfrag connections as inner links between genomic regions (5' and 3' RACE connections are red and blue, respectively). The circular tracks are, going inwards: (1) - chromosome scale (in megabases, starting at 14 Mb), (2) - plus-strand annotated genes (green), (3) - plus-strand annotated pseudogenes, (4) - minus-strand annotated genes, (5) - minus-strand annotated pseudogenes.

**Figure S7: Chromosome 22 transcriptional networks.**

See legend of figure S6.

**Figure S8: Reciprocal gene to gene connections in chromosome 21 (A) and 22 (B).**

All 2,324 pure and composite gene/gene reciprocal connections observed in the 10 cell types studied are represented as blue (connection involving two genes on the same chromosome strand) and orange (connection involving two genes on different strands) inner ribbons. See Figure 2A for further legend details. Pseudogene tracks were removed for clarity purposes (See figures S9 and S10 for reciprocal gene/gene connections in each cell type).

**Figure S9: Reciprocal gene to gene connections observed in each cell type on chromosome 21.**

Networks of reciprocal gene to gene connections observed in each of the 10 assayed cell types are represented as blue (connection involving two genes on the same chromosome strand) and orange (connection involving two genes on different strands) inner ribbons. See figures S6 and S7 for further legend details. Pseudogene tracks were removed for clarity purposes.

**Figure S10: Reciprocal gene to gene connections observed in each cell type on chromosome 22.**

See legend of figure S9.

**Figure S11: Pairwise correlations between cell types based on pure reciprocal gene to gene connections.**

This figure represents the pairwise correlations between the cell types used in the RACEarray experiments as a heatmap: the closer to the white, the more correlated. More precisely for each pair of cell types, the Pearson's product moment correlation between them was computed based on the number of reciprocal gene to gene connections commonly observed, in the universe of all possible reciprocal gene to gene connections. This number is the one indicated in the corresponding cell of the heatmap. Note that genes g_1_ and g_2_ form a possible reciprocal gene to gene connection if and only if there is a RACE primer in g_1_ pointing in the direction of g_2_ and a RACE primer in g_2_ pointing in the direction of g_1_.

**Figure S12: Number of observed (left) and of expected (right) gene to gene connections on chromosomes 21 (top) and 22 (bottom).**

The shape of the observed distributions is similar for the two chromosomes, as well as the shape of the expected ones, however the distributions are decreasing much more rapidly for the expected connections compared to the observed connections.

**Figure S13: Difference between number of observed and number of expected gene to gene connections on chromosome 21 (A) and on chromosome 22 (B).**

These two histograms (A and B) represent the distributions of the difference between the number of observed and the number of expected gene to gene connections for reciprocally connected genes on chromosomes 21 and 22 respectively. These distributions are shifted towards the positive values, and have a mean of 6 and 5 respectively. In our analysis the difference between the number of observed and the number of expected gene to gene connections of reciprocally connected genes is used as a score for those genes and is used to delineate a set of genes much more connected than we would expect given their length and number of primers: the hubs.

**Figure S14: Different categories of genes used in the RACEarray experiments.**

Proportional Venn diagram representation of inclusion relationships between some of the most used sets of genes used in this study. The area highlighted in light blue corresponds to non-hub genes, which are all reciprocally connected.

**Figure S15: Expected number of gene to gene connections found by RACEarray and RNA PET ditags in K562.**

**Figure S16: Expected number of gene to gene connections found by RACEarray and Illumina Human Body Map PE50 RNAseq in Testes+Prostate (A) and in Brain (B).**

**Figure S17:** **Interspecies chimeric RNAs used as a metric of technical artifacts.**

The number of reads/10M total reads for intra-genomic and inter-genomic chimeric junction

sites is plotted human and fly alone and various ratios of RNAs from human and fly

(mixtures). A total of at least 25 nucleotides on each side of a chimeric junction site was

chosen as a minimum to allow for unique mapping in each genome.

**Figure S18: 5C data validates RACEarray gene to gene connections.**

This figure represents the distribution of the proportion of gene to gene connections validated by 5C in 1,000 sets of gene to gene connections detectable by RACEarray and by 5C with the same distributions of distance between connected genes and of length of connected genes as in the 638 connections detectable by both techniques that are actually observed. The mean of this distribution is 70.8 (standard deviation=1.9), which is significantly lower than the observed proportion (496/638=77.7%, depicted by the arrow on the right, p-value <10^-3^).

**Figure S19: Domain organization for chimera OTTHUMP00000221101.**

Chimera OTTHUMP00000221101 results from the fusion of two receptors involved in immune response, Interferon-alpha/beta receptor 2 (IFNAR, N-terminal section) and Interleukin-10 receptor subunit beta (IL10RB, C-terminal section). The resulting protein will have an extra-cellular domain that is double the size of the usual extra-cellular receptor domain and that is composed of a repeat of paired tissue factor (green) and alpha/beta interferon receptor (red) domains. The chimeric proteins also conserves a signal peptide signal and a single trams-membrane helix. A similar domain configuration is recorded in Uniprot for the chcken interferon receptor (Q5XPI1_CHICK).

**Figure S20: Model of possible structure of fused fragments for chimera OTTHUMP00000221101.**

Models for the N- and C-terminal sections have been obtained respectively from structures 2hym and 3g9v by comparative modelling (Modeller, http://salilab.org/modeller). Linker region (shown as a gap in the structure) is located in flexible regions for both templates. Domain folds could then be maintained independently.

**Table legends**

**TableS1: Number of gene to gene and number of reciprocal gene to gene connections by distance.**

This table is similar to Figure 5B and provides numbers of gene to gene and of reciprocal gene to gene connections detected in each cell type, split by distance bins:

- < 150kb
- 150kb – 1Mb
- 1Mb – 5Mb
- > 5Mb.

This table shows that (1) the number of connections is similar in cell lines and tissues, (2) the distribution of connections in distance classes changes if we consider all or only reciprocal connections, (3) between one third and half of the reciprocal connections are cell-type specific and (4) all the figures are quite high meaning that chimeras are far from being exceptional.

**Table S2: Validation results of chimeric transcripts by RNase protection assays.**

This table lists the RACE name, the pool of poly-A+ RNA used, description of the probes, a summary of the RNase Protection Assay screening with a detailed interpretation of the results based on the autoradiography gel.

**Table S3: Names and characteristics of the hubs.**

For each of the 74 hubs the table provides:

- the number of observed connections,

- the number of expected connections,

- the difference between the two, which could be seen as their connectivity score.

**Table S4: Hubs have higher phylogenetic depth than non hubs.**

We consider four different gene sets (see supplementary figure S14 for a description):

- RACE interrogated (“raced”) genes
- reciprocally connected genes
- hubs
- non hubs

and for each of them, we provide the number of genes with an *Ensembl* gene ID, and the number and the proportion of this total, that has an ortholog in the 6 following species, as found using biomart on ensembl51:

- Yeast
- C. *elegans*
- *Drosophila*
- *Tetraodon*
- Chicken
- Mouse

For each of these species we then provide the Fisher exact test p-value obtained while testing the following hypothesis: “Being a hub is independent on having an ortholog in a given species”. A star above this number on the table means the p-value is significant (less than 0.01). Note that both the proportions of genes with an ortholog in each of the 6 species for RACE interrogated (“raced”) genes, hubs and non hubs, and the significance of the Fisher tests mentioned here are provided on Figure 9B.

**Table S5: Over-representation of cliques in chromosomes 21 and 22.**

For each chromosome, we report the number of cliques observed, as well as the mean and the standard deviation of the number of cliques expected.

**Table S6: Constitutive cliques.**

For each constitutive clique (the maximum size is 3), we provide:

- the names of the genes involved in the clique,
- the chromosome where the clique is observed,
- the list of cell types in which the clique is observed.

**Table S7: Overlap of maximal cliques.**

For each clique size, we report:

- the number of cliques observed,
- the number of corresponding edges if cliques were not overlapping,
- the number of observed edges.

**Supplementary data files**

1) Manually annotated exons:

<ftp://genome.crg.es/pub/Encode/ChimericRNAChr2122/suppl_materials_manual_annotation.zip>

2) RACE primers:

<ftp://genome.crg.es/pub/Encode/ChimericRNAChr2122/suppl_materials_RACE_primer.zip>

3) RACEfrags:

<ftp://genome.crg.es/pub/Encode/ChimericRNAChr2122/suppl_materials_RACEfrags.zip>

4) RT-PCR primer sequences and positive RT-PCR products:

<ftp://genome.crg.es/pub/Encode/ChimericRNAChr2122/suppl_materials_RT-PCR_sequences.zip>

5) Expression profiles of annotated genes based on tiling array data in 16 tissues and cell lines:

<ftp://genome.crg.es/pub/Encode/ChimericRNAChr2122/suppl_materials_gene_expression_profiles.zip>

6) 5C data on chromosome 21 for K562 and GM06990 cell lines:

<ftp://genome.crg.es/pub/Encode/ChimericRNAChr2122/suppl_materials_5C_data.zip>

7) Supplementary table S1:

<ftp://genome.crg.es/pub/Encode/ChimericRNAChr2122/tabS1.xlsx>

**References**

**1. Harrow, J., et al., *GENCODE: producing a reference annotation for ENCODE.* Genome Biol, 2006. 7 Suppl 1: p. S4 1-9.**

**2. Collins, J.E., et al., *Reevaluating human gene annotation: a second-generation analysis of chromosome 22.* Genome Res, 2003. 13(1): p. 27-36.**

**3. Kent, W.J., *BLAT--the BLAST-like alignment tool.* Genome Res, 2002. 12(4): p. 656-64.**

**4. Rozen, S. and H. Skaletsky, *Primer3 on the WWW for general users and for biologist programmers.* Methods Mol Biol, 2000. 132: p. 365-86.**

**5. Djebali, S., et al., *Efficient targeted transcript discovery via array-based normalization of RACE libraries.* Nat Methods, 2008. 5(7): p. 629-35.**

**6. Denoeud, F., et al., *Prominent use of distal 5' transcription start sites and discovery of a large number of additional exons in ENCODE regions.* Genome research, 2007. 17(6): p. 746-59.**

**7. Blanco, E., G. Parra, and R. Guigo, *Using geneid to identify genes.* Curr Protoc Bioinformatics, 2007. Chapter 4: p. Unit 4 3.**

**8. Salehi-Ashtiani, K., et al., *Isoform discovery by targeted cloning, 'deep-well' pooling and parallel sequencing.* Nature methods, 2008. 5(7): p. 597-600.**

**9. Walhout, A.J., et al., *GATEWAY recombinational cloning: application to the cloning of large numbers of open reading frames or ORFeomes.* Methods enzymol, 2000. 328: p. 575-92.**

**10. Gordon, D., C. Abajian, and P. Green, *Consed: a graphical tool for sequence finishing.* Genome research, 1998. 8(3): p. 195-202.**

**11. Searle, S.M., et al., *The otter annotation system.* Genome Res, 2004. 14(5): p. 963-70.**

**12. Altschul, S.F., et al., *Basic local alignment search tool.* J Mol Biol, 1990. 215(3): p. 403-10.**

**13. Sonnhammer, E.L. and J.C. Wootton, *Integrated graphical analysis of protein sequence features predicted from sequence composition.* Proteins, 2001. 45(3): p. 262-73.**

**14. Durbin, R. and E. Griffiths, *Acedb genome database*, in *Genetics, Genomics, Proteomics and Bioinformatics Online. Bioinformatics. Modern paradigms in Biology.*, B.C. Peter Clote, Massachusetts, USA. Willey Interscience, Editor. 2005.**

**15. Wilming, L.G., et al., *The vertebrate genome annotation (Vega) database.* Nucleic Acids Res, 2008. 36(Database issue): p. D753-60.**

**16. Milo, R., et al., *Network motifs: simple building blocks of complex networks.* Science, 2002. 298(5594): p. 824-7.**

**17. Khanin, R. and E. Wit, *How scale-free are biological networks.* J Comput Biol, 2006. 13(3): p. 810-8.**

**18. Csárdi, G. and T. Nepusz, *The igraph software package for complex network research.* InterJournal Complex Systems, 2006. 1695.**
