# Supplementary figures and images for: Evidence for Transcript Networks Composed of Chimeric RNAs in Human Cells
Source: PLoS One. 2012 Jan 4;7(1):e28213. doi: 10.1371/journal.pone.0028213 (PMC3251577; doi:10.1371/journal.pone.0028213)

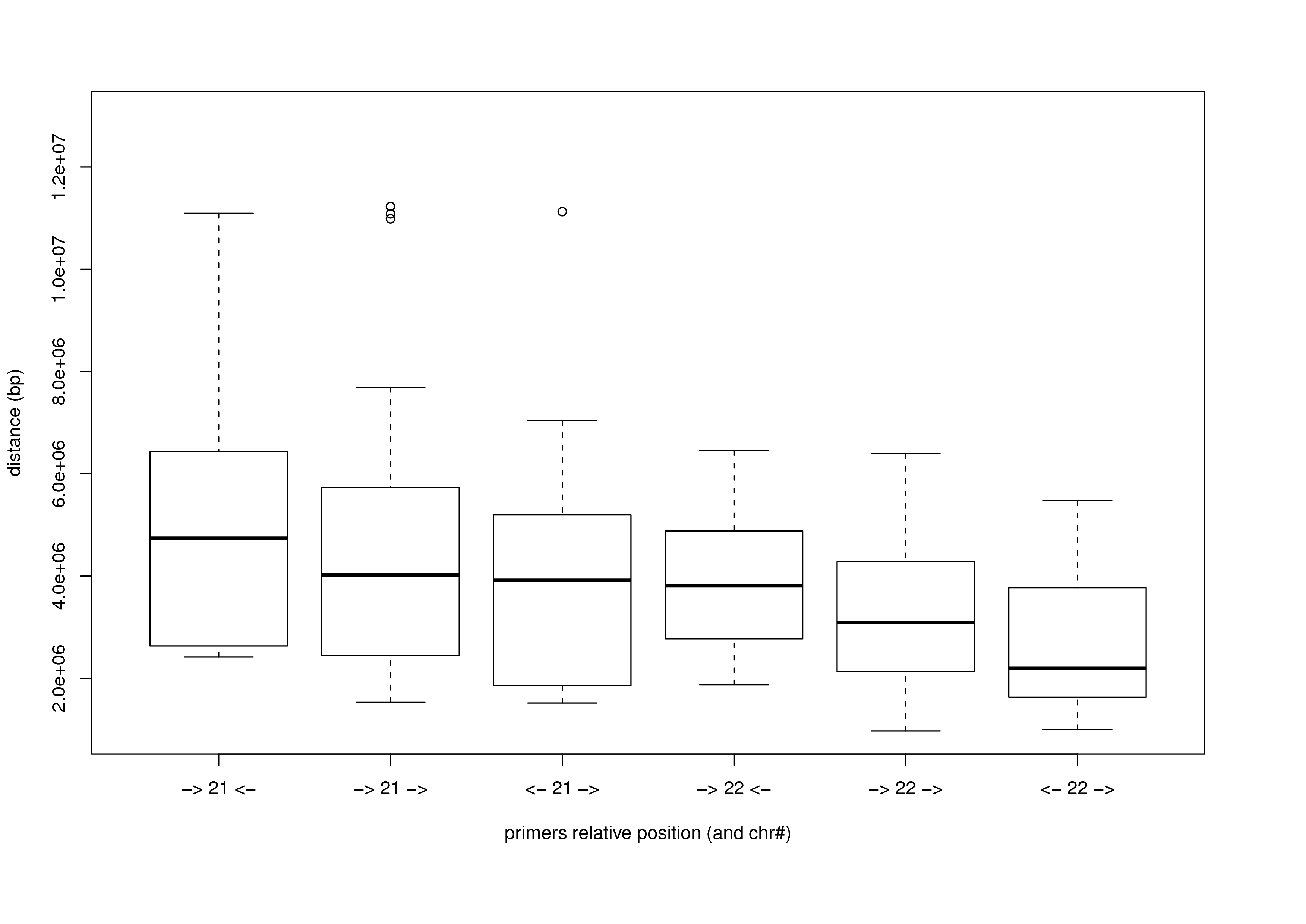

Supplement: Figure S1 — Distance between consecutive pooled primers. This figure represents the distribution of distances between consecutive primers of the same pool for each chromosome and each relative position of primer pairs: head to head (→ ←), head to tail (→→) and tail to tail (←→), as a box plot. The gene density of chromosome 22 being higher than that of chromosome 21, the distance between consecutive pooled primers is smaller for the latter than for the former. Also, as expected, the distance between consecutive pooled primers is higher for head to head than for head to tail configuration, and higher for head to tail than for tail to tail configuration. (TIFF) [file pone.0028213.s002.tif]

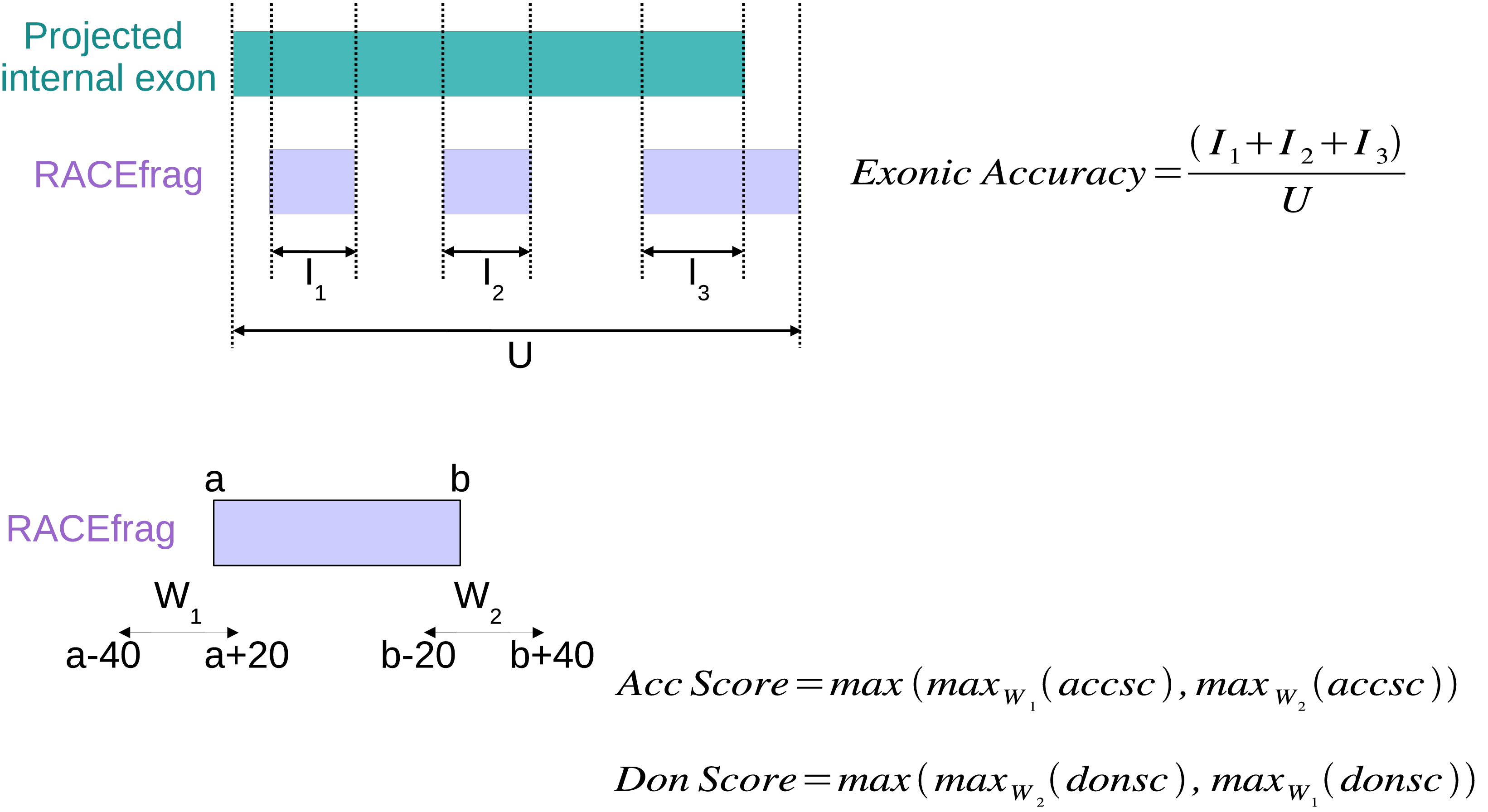

Supplement: Figure S2 — Two measures to assess RACEfrag sets. This figure describes the two measures used to assess RACEfrag sets while optimizing the parameters used for RACEfrag calling: the exonic accuracy and the splice site score. The exonic accuracy assesses the RACEfrag set with respect to a reference set: the projected internal exons. More precisely for each projected internal exon, considered as a reference, the exonic accuracy assesses the accuracy with which the RACEfrags overlapping this reference mimics this reference. This accuracy is measured in terms of intersection over union of the projected internal exons and the RACEfrags, i.e., for each projected internal exon with overlapping RACEfrags, the number of nucleotides in common between the two sets is divided by the number of nucleotides in either of the two sets. The exonic accuracy of a RACEfrag set is then the median of the exonic accuracy of projected internal exons with overlapping RACEfrags. Unlike the exonic accuracy the splice site score of a RACEfrag set does not depend on any reference but is rather intrinsic to the RACEfrag set. More precisely, the spice site score is divided into two sub-measures: the acceptor score and the donor score. Both scores involve the scanning of two windows around the RACEfrag boundaries, W1 around the left boundary and W2 around the right boundary, where both acceptor and donor sites have previously been found by the geneid program. The RACEfrag acceptor score is then defined as the score of the best acceptor site on the 2 windows, and the RACEfrag donor score as the one of the best donor site on the 2 windows. (TIFF) [file pone.0028213.s003.tif]

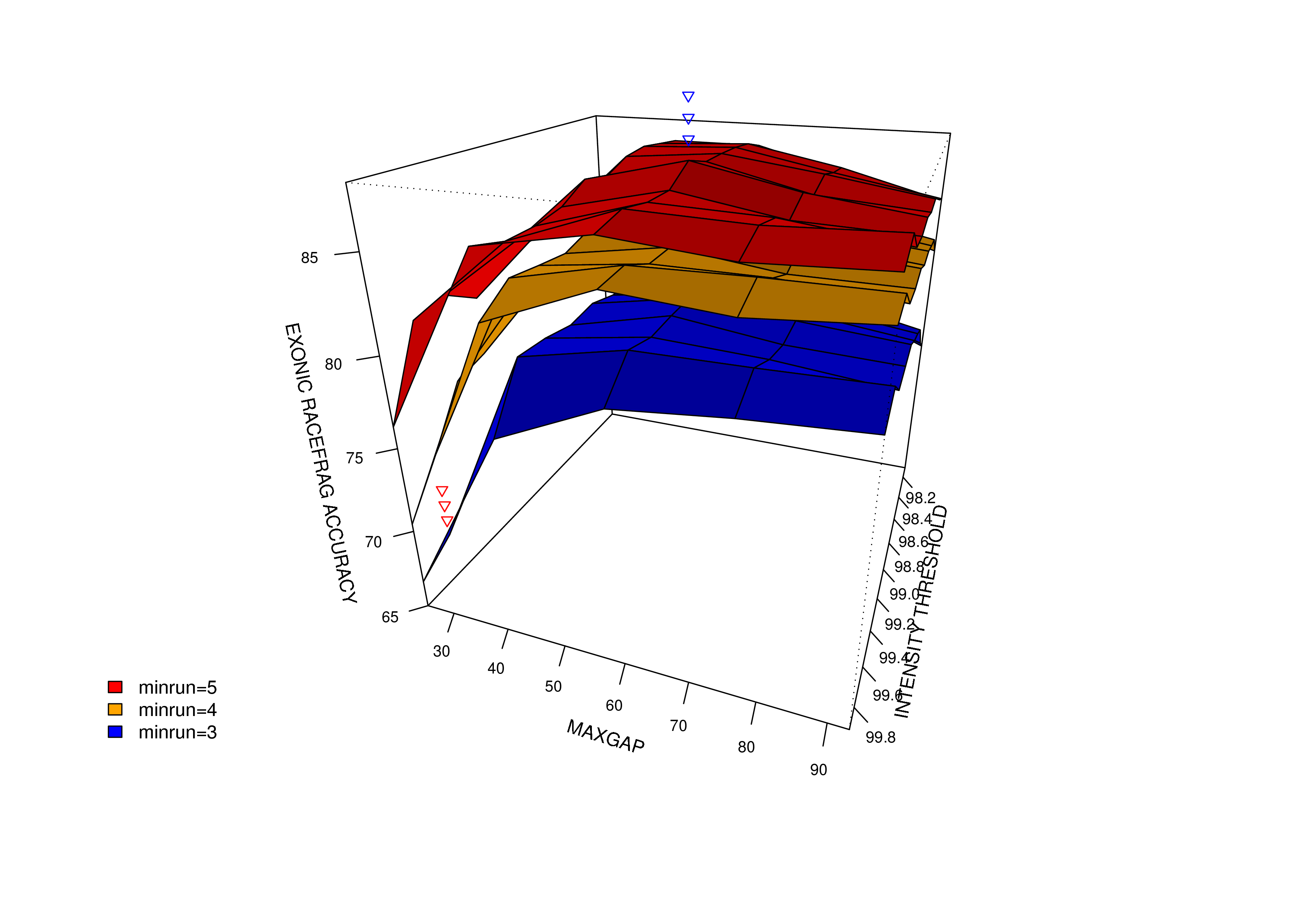

Supplement: Figure S3 — RACEfrag calling. This figure represents the exonic accuracy of 10 RACEfrag sets coming from 10 randomly chosen experiments, as a function of the intensity threshold (I) and the maxgap (M), for 3 different minrun values (m): 3, 4 and 5. The blue arrows indicate the maximum exonic accuracy found over all the possible values of the three parameters, and the red arrows the minimum exonic accuracy. The maximum is reached for I = 99.1%ile, M = 59 bp, m = 5 probes. (TIFF) [file pone.0028213.s004.tif]

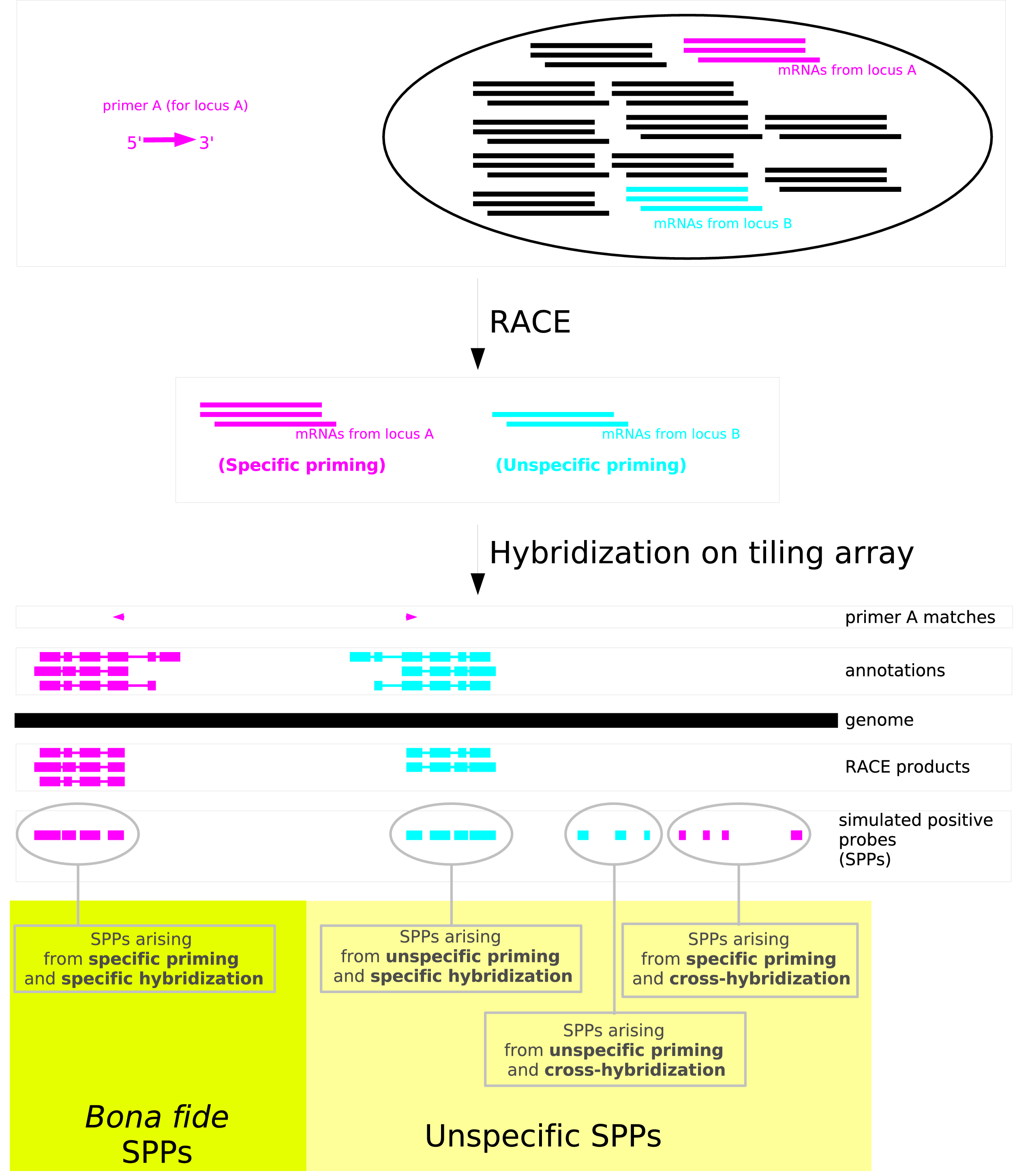

Supplement: Figure S4 — USPP filter. This simulation involves two steps: (1) RACE from a set of primers and a set of known transcripts; (2) hybridization of the obtained RACE products on tiling arrays. The RACEarray simulator generates a set of tiling array probes that are highlighted by the RACE products and that we call simulated positive probes (SPPs). These SPPs can be further divided into two categories: (1) bona fide SPPs, i.e. overlapping an exon of the target locus; (2) unspecific SPPs, also called USPPs, i.e. mapping outside of the target locus exons. In our model these USPPs correspond to false positives that originate from RACE mis-priming and/or from array cross-hybridization (see text for a more detailed explanation). (TIFF) [file pone.0028213.s005.tif]

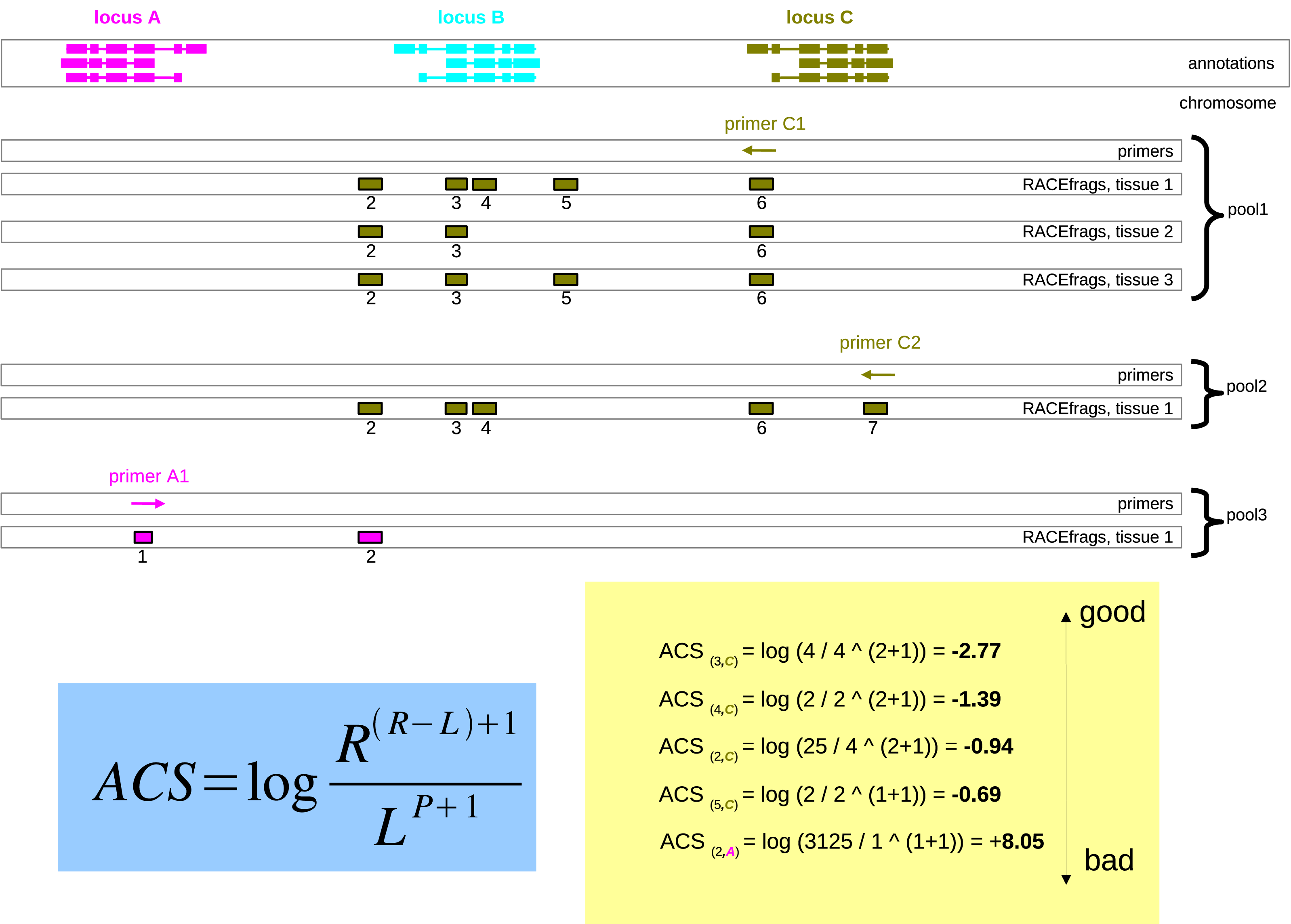

Supplement: Figure S5 — RACEfrag assignment. This figure is divided into three parts: 1) on the top, the annotations of a given chromosome are represented, which are here the different alternative transcripts of three loci: A, B and C; 2) in the middle, the primers and RACEfrags of three different pools in several tissues are represented; 3) on the bottom, the formula of the assignment confidence score is provided again as well as its application on 5 different (RACEfrag, locus) pairs (note that here two RACEfrags with the same coordinates are given the same identifier). The first two parts of the figure are thus dedicated to the description of the assignment method, while the third part shows how the assignment score behaves on already assigned RACEfrags associated to their locus. Primers are named and colored after the locus they are originating from, and RACEfrags after the locus they have been assigned to. In pool 1, primer C1 is active and points in the direction of all RACEfrags, so all RACEfrags of pool 1 are assigned to primer C1. In pool 2, it is the same with primer C2, and in pool 3 the same with primer A1. Then the ACS formula is applied to 5 different (RACEfrag, locus) pairs, and the lower the score the more confidence we have in the assignment of the RACEfrag to the locus. Here the (RACEfrag, locus) pair we are the most confident in is (3,C) since RACEfrag 3 appears 4 times in total and each time it appears it is assigned to locus C. Also, the fact that it is assigned to two different primers of locus C, primers C1 and C2, strengthens the confidence we have in this pair. The pair (4,C) is similar to the pair (3,C) except that RACEfrag 4 appears in 2 experiments instead of 4. It thus also has a good score, although less than the one of (3,C). The pair (2,C) is like the pair (3,C) except that RACEfrag 2 also appears in pool 3, tissue 1 where it is assigned to locus A. This makes it more uncertain we should assign RACEfrags 2 to locus C, as compared to RACEfrag 3, and this is wh [file pone.0028213.s006.tif]

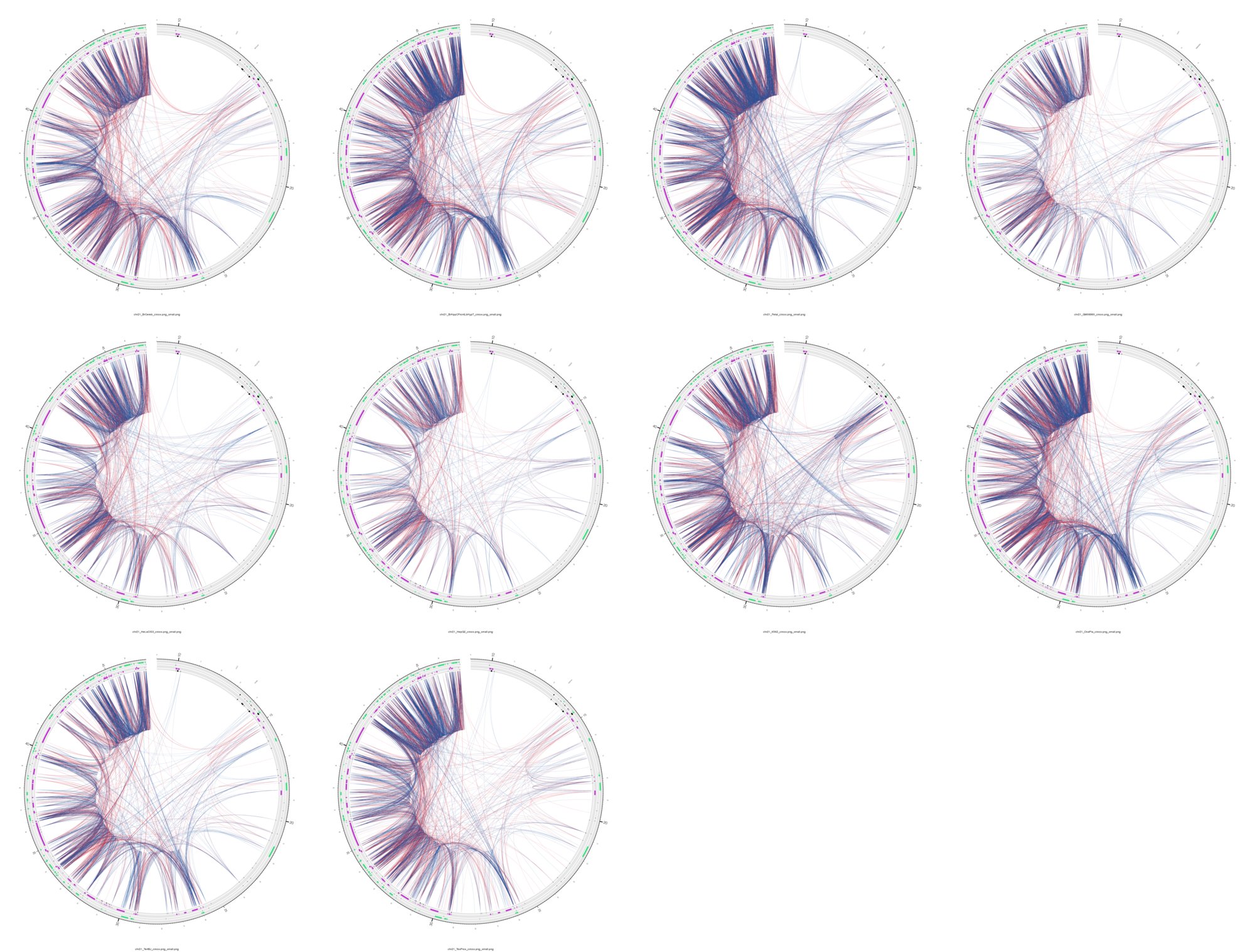

Supplement: Figure S6 — Chromosome 21 transcriptional networks. RACE connection networks in all 10 assayed cell types are represented. In each plot, the chromosome is depicted as a circle, and RACEfrag connections as inner links between genomic regions (5′ and 3′ RACE connections are red and blue, respectively). The circular tracks are, going inwards: (1) - chromosome scale (in megabases, starting at 14 Mb), (2) - plus-strand annotated genes (green), (3) - plus-strand annotated pseudogenes, (4) - minus-strand annotated genes, (5) - minus-strand annotated pseudogenes. (JPG) [file pone.0028213.s007.jpg]

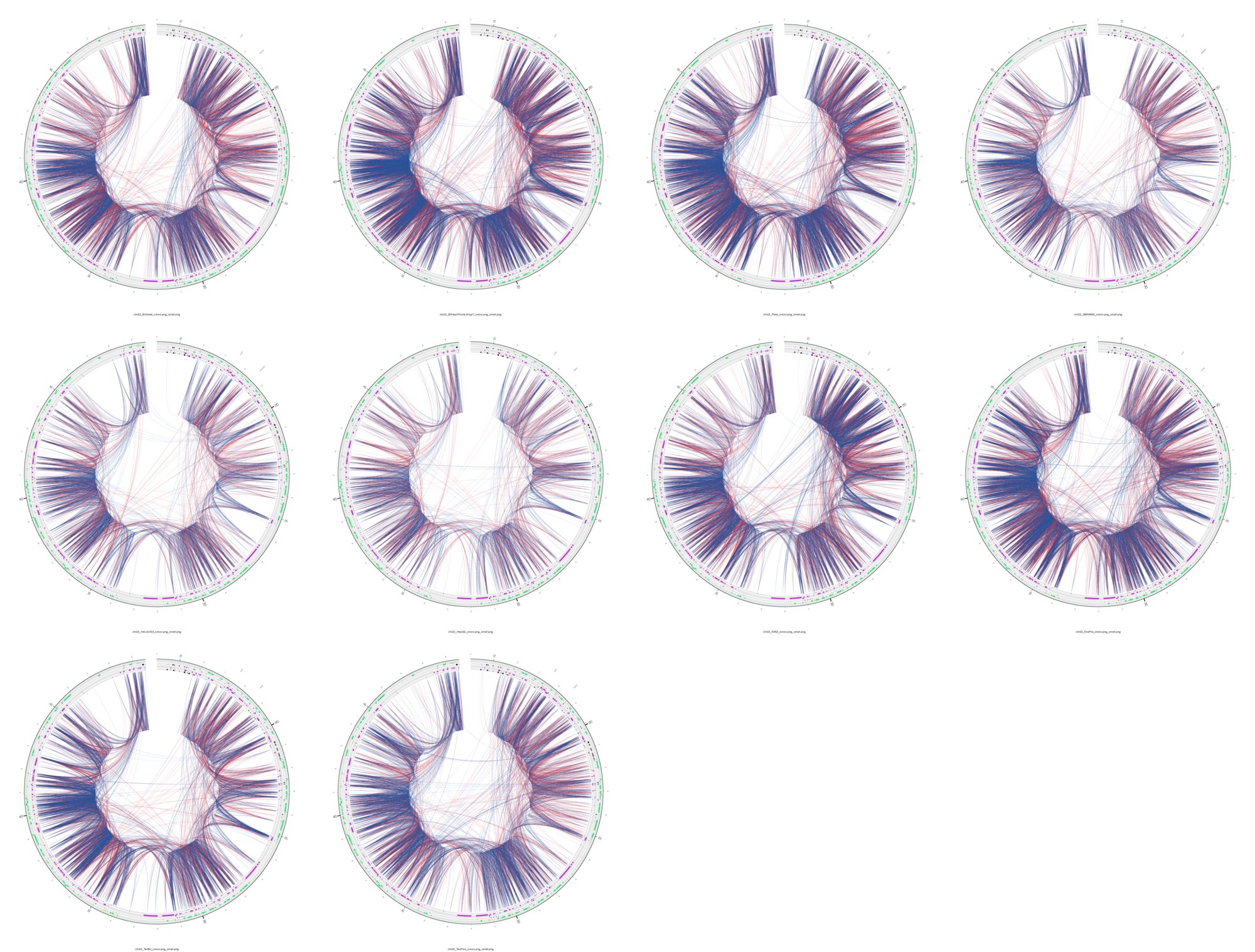

Supplement: Figure S7 — Chromosome 22 transcriptional networks. See legend of figure S6. (JPG) [file pone.0028213.s008.jpg]

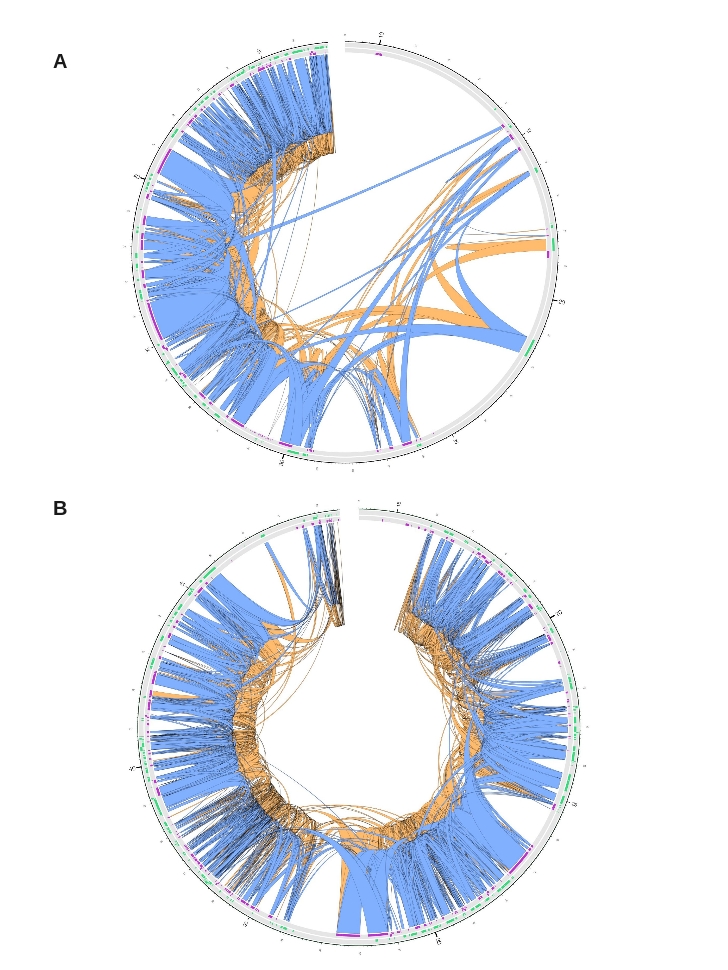

Supplement: Figure S8 — Reciprocal gene to gene connections in chromosome 21 (A) and 22 (B). All 2,324 pure and composite gene/gene reciprocal connections observed in the 10 cell types studied are represented as blue (connection involving two genes on the same chromosome strand) and orange (connection involving two genes on different strands) inner ribbons. See Figure 2A for further legend details. Pseudogene tracks were removed for clarity purposes (See Figures S9 and S10 for reciprocal gene/gene connections in each cell type). (JPG) [file pone.0028213.s009.jpg]

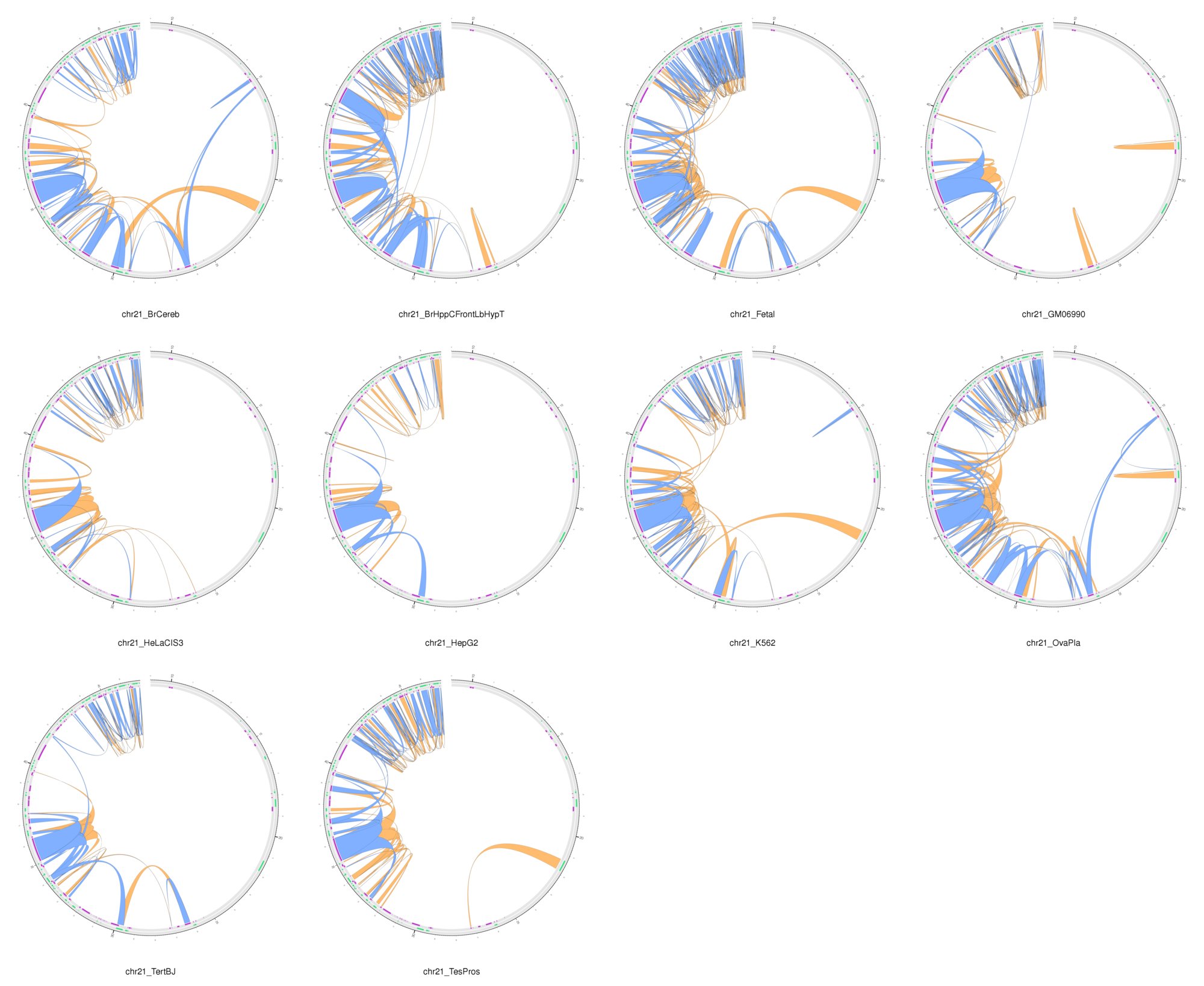

Supplement: Figure S9 — Reciprocal gene to gene connections observed in each cell type on chromosome 21. Networks of reciprocal gene to gene connections observed in each of the 10 assayed cell types are represented as blue (connection involving two genes on the same chromosome strand) and orange (connection involving two genes on different strands) inner ribbons. See Figures S6 and S7 for further legend details. Pseudogene tracks were removed for clarity purposes. (JPG) [file pone.0028213.s010.jpg]

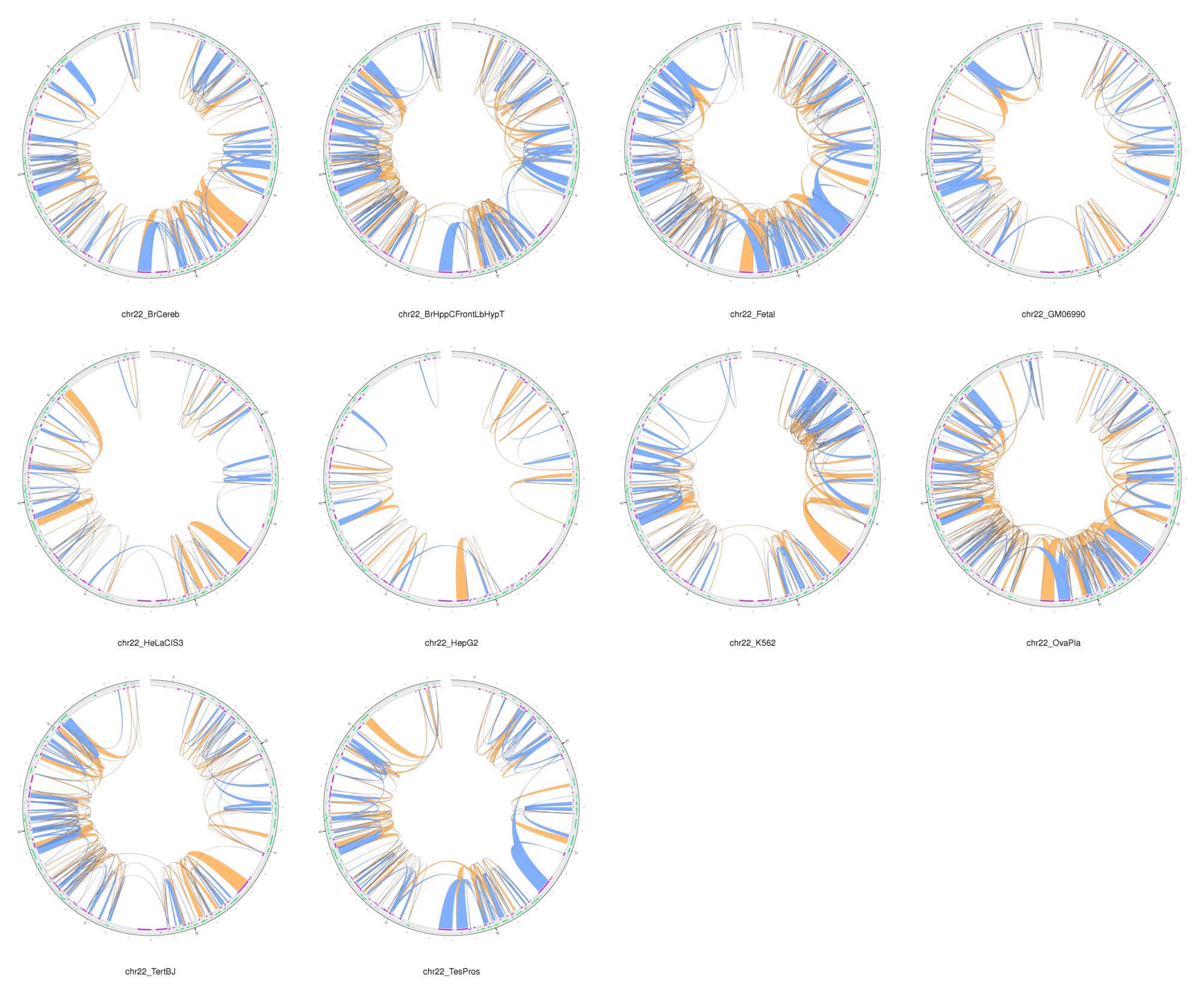

Supplement: Figure S10 — Reciprocal gene to gene connections observed in each cell type on chromosome 22. See legend of Figure S9. (JPG) [file pone.0028213.s011.jpg]

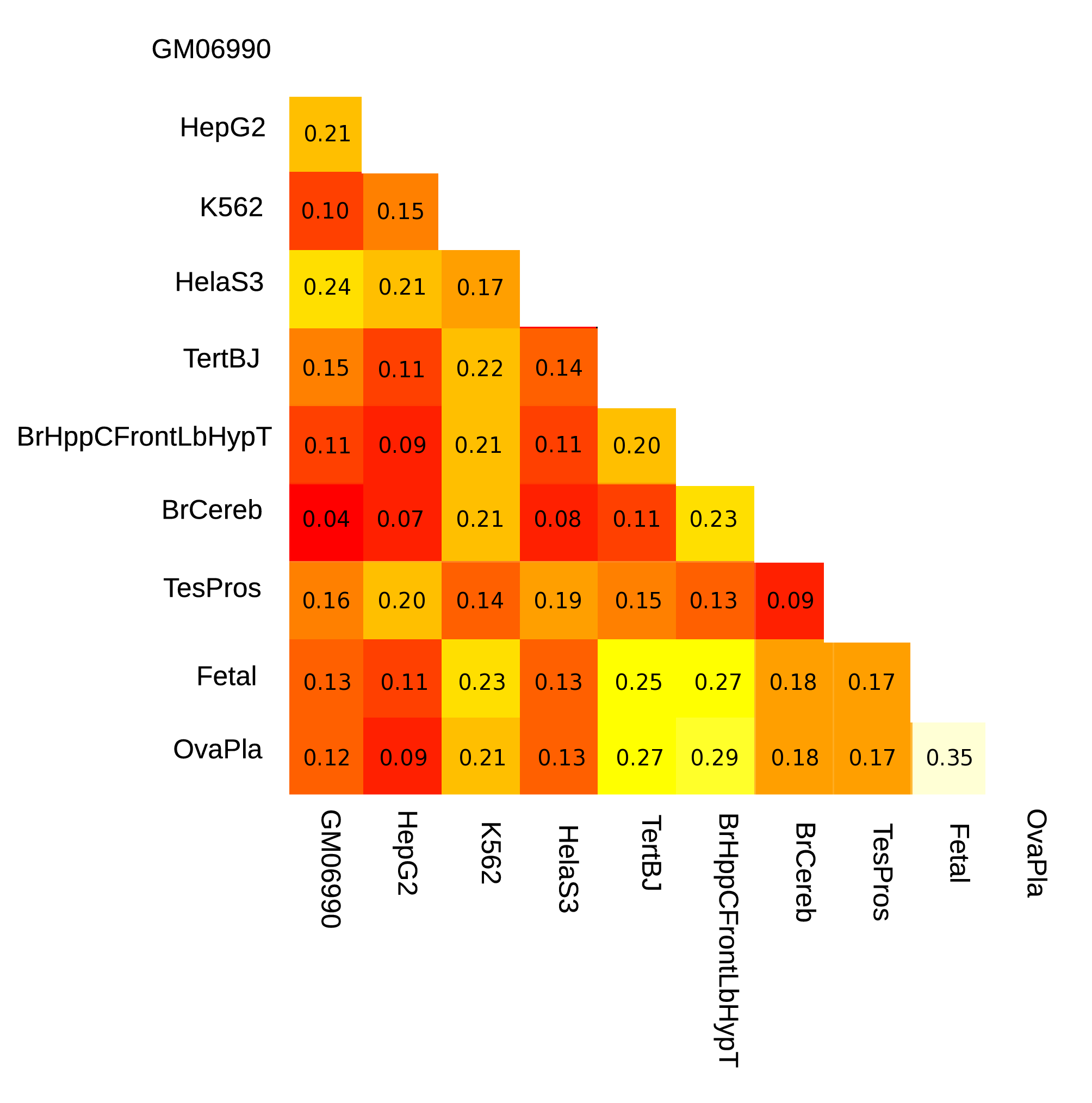

Supplement: Figure S11 — Pairwise correlations between cell types based on pure reciprocal gene to gene connections. This figure represents the pairwise correlations between the cell types used in the RACEarray experiments as a heatmap: the closer to the white, the more correlated. More precisely for each pair of cell types, the Pearson's product moment correlation between them was computed based on the number of reciprocal gene to gene connections commonly observed, in the universe of all possible reciprocal gene to gene connections. This number is the one indicated in the corresponding cell of the heatmap. Note that genes g1 and g2 form a possible reciprocal gene to gene connection if and only if there is a RACE primer in g1 pointing in the direction of g2 and a RACE primer in g2 pointing in the direction of g1. (PNG) [file pone.0028213.s012.png]

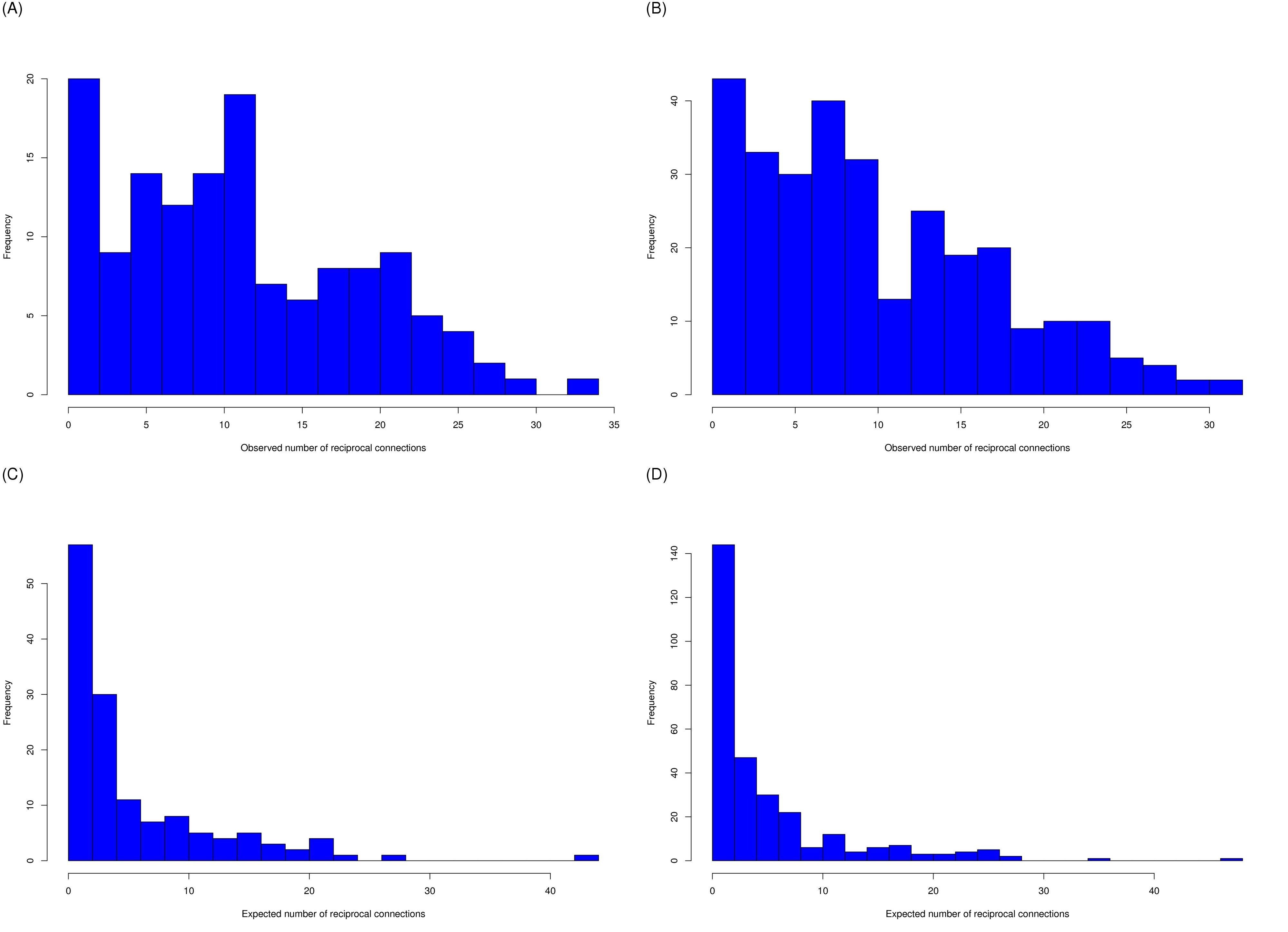

Supplement: Figure S12 — Number of observed (left) and of expected (right) gene to gene connections on chromosomes 21 (top) and 22 (bottom). The shape of the observed distributions is similar for the two chromosomes, as well as the shape of the expected ones, however the distributions are decreasing much more rapidly for the expected connections compared to the observed connections. (PNG) [file pone.0028213.s013.png]

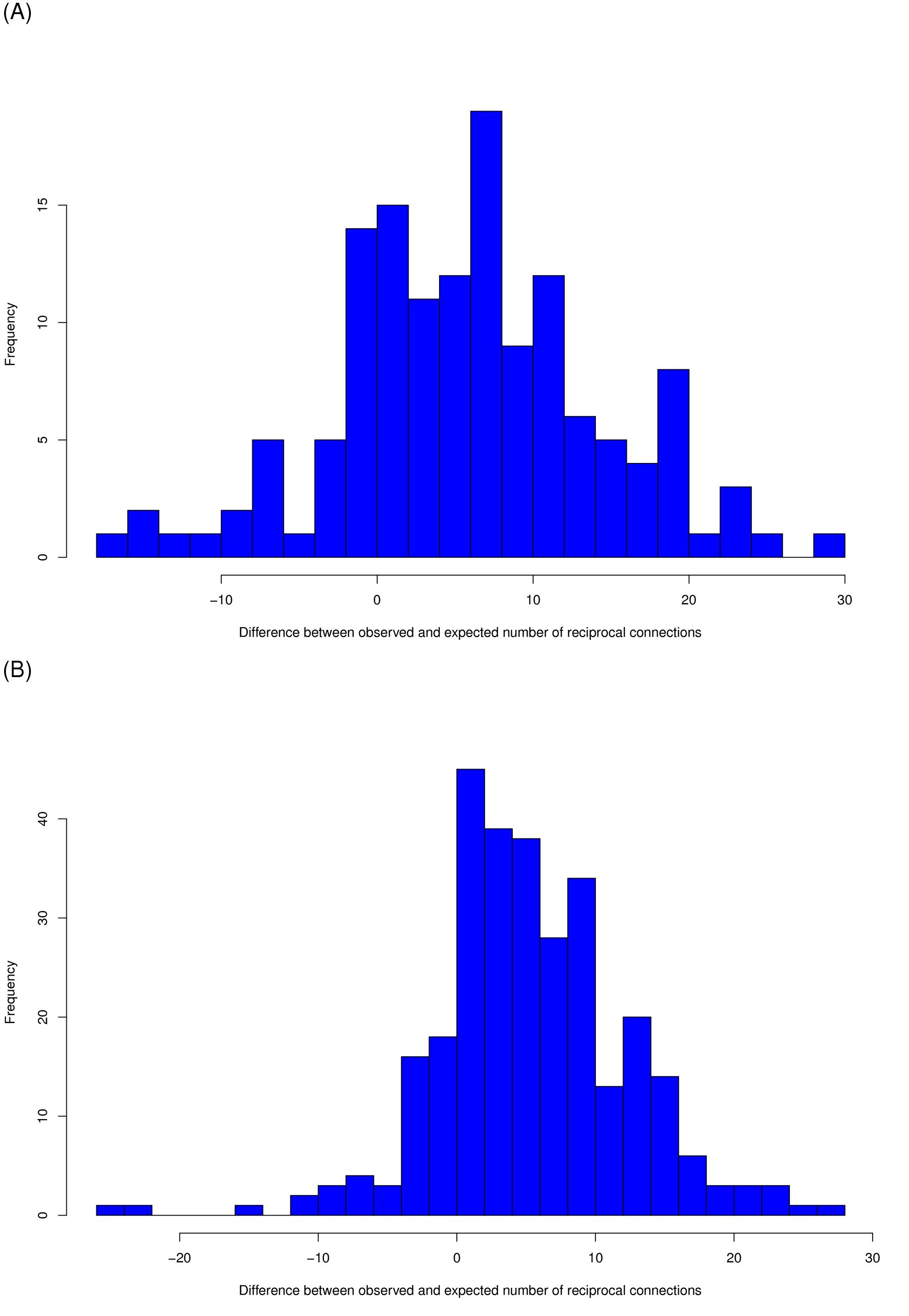

Supplement: Figure S13 — Difference between number of observed and number of expected gene to gene connections on chromosome 21 (A) and on chromosome 22 (B). These two histograms (A and B) represent the distributions of the difference between the number of observed and the number of expected gene to gene connections for reciprocally connected genes on chromosomes 21 and 22 respectively. These distributions are shifted towards the positive values, and have a mean of 6 and 5 respectively. In our analysis the difference between the number of observed and the number of expected gene to gene connections of reciprocally connected genes is used as a score for those genes and is used to delineate a set of genes much more connected than we would expect given their length and number of primers: the hubs. (PNG) [file pone.0028213.s014.png]

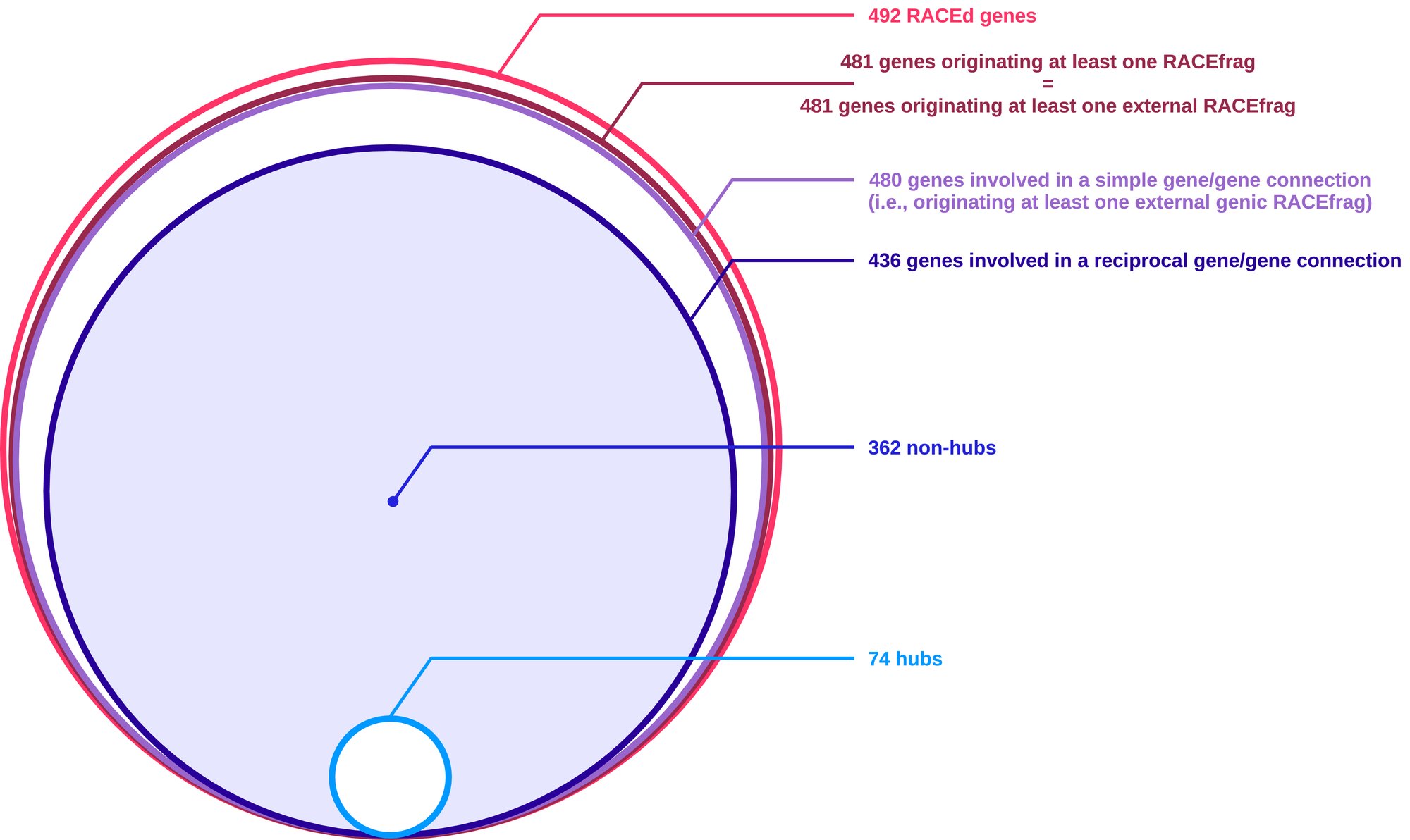

Supplement: Figure S14 — Different categories of genes used in the RACEarray experiments. Proportional Venn diagram representation of inclusion relationships between some of the most used sets of genes used in this study. The area highlighted in light blue corresponds to non-hub genes, which are all reciprocally connected. (PNG) [file pone.0028213.s015.png]

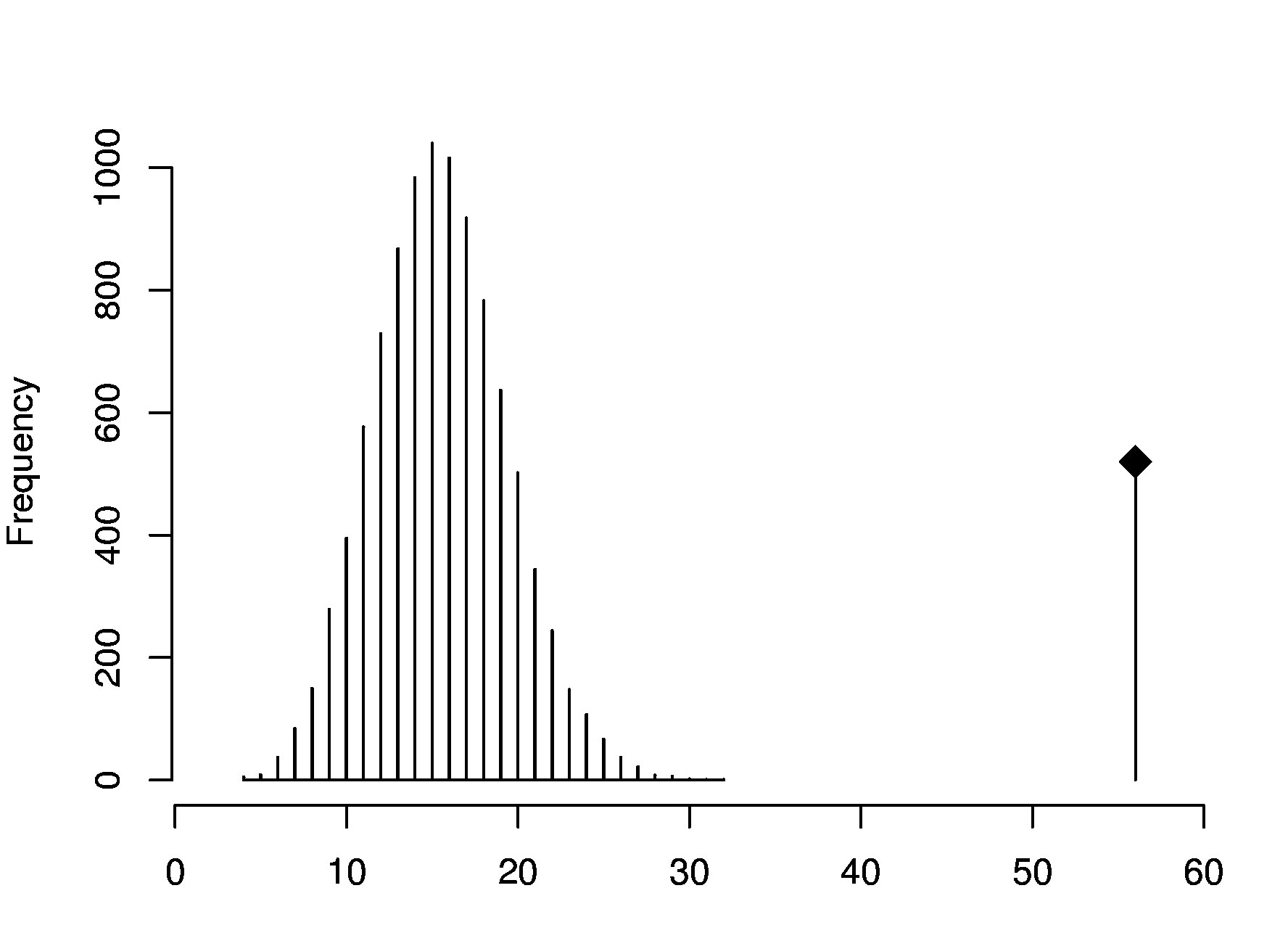

Supplement: Figure S15 — Expected number of gene to gene connections found by RACEarray and RNA PET ditags in K562. (JPG) [file pone.0028213.s016.jpg]

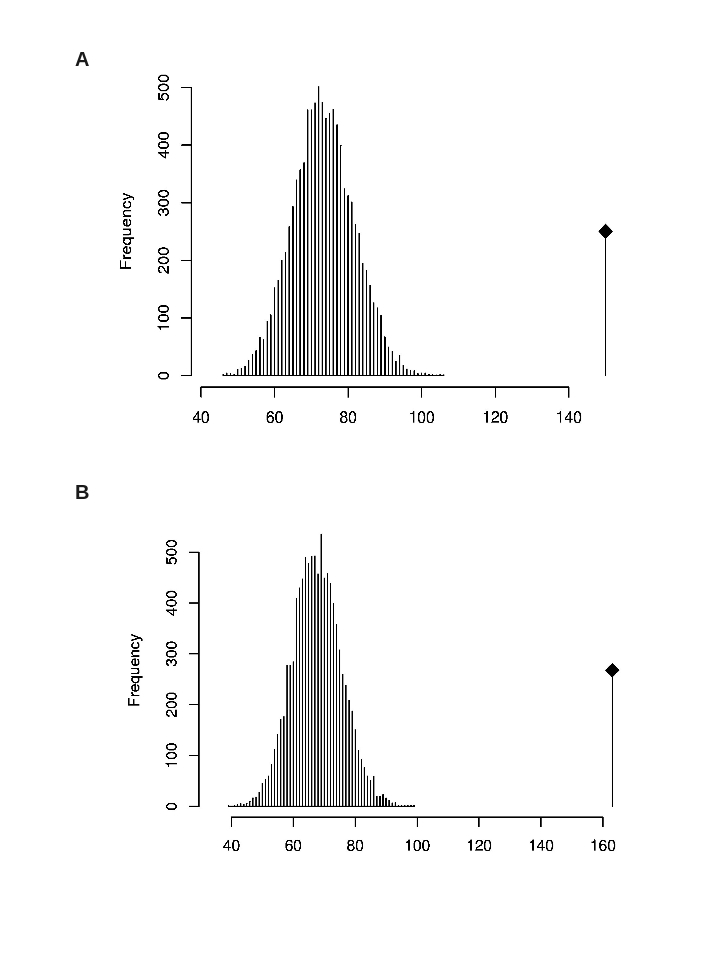

Supplement: Figure S16 — Expected number of gene to gene connections found by RACEarray and Illumina Human Body Map PE50 RNAseq (A) in Testes+Prostate and in Brain (B). (JPG) [file pone.0028213.s017.jpg]

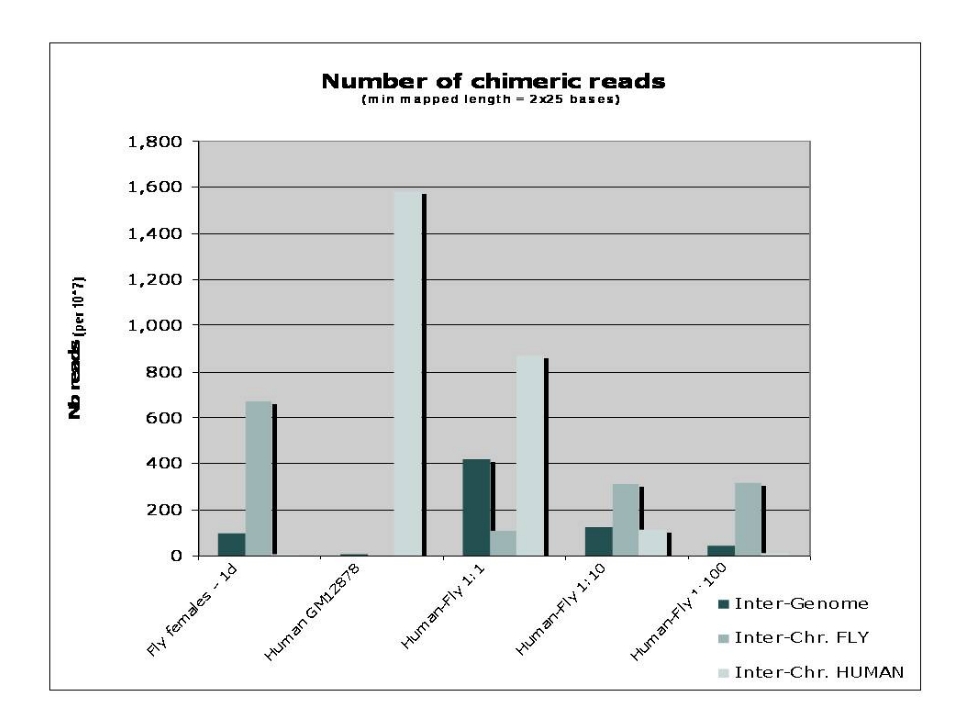

Supplement: Figure S17 — Interspecies chimeric RNAs used as a metric of technical artifacts. The number of reads/10 M total reads for intra-genomic and inter-genomic chimeric junction sites is plotted human and fly alone and various ratios of RNAs from human and fly (mixtures). A total of at least 25 nucleotides on each side of a chimeric junction site was chosen as a minimum to allow for unique mapping in each genome. (JPG) [file pone.0028213.s018.jpg]

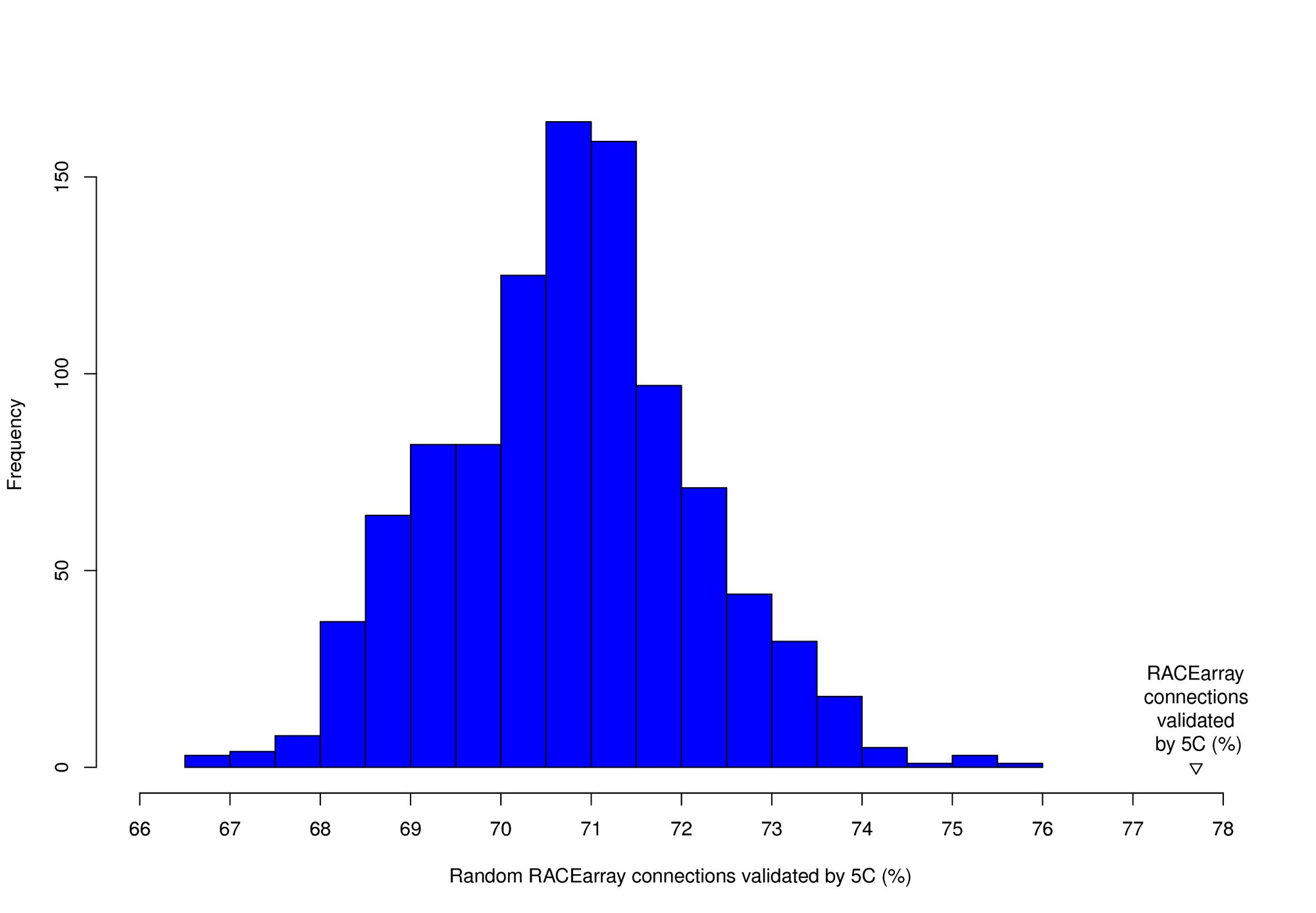

Supplement: Figure S18 — 5C data validates RACEarray gene to gene connections. This figure represents the distribution of the proportion of gene to gene connections validated by 5C in 1,000 sets of gene to gene connections detectable by RACEarray and by 5C with the same distributions of distance between connected genes and of length of connected genes as in the 638 connections detectable by both techniques that are actually observed. The mean of this distribution is 70.8 (standard deviation = 1.9), which is significantly lower than the observed proportion (496/638 = 77.7%, depicted by the arrow on the right, p-value<10−3). (JPG) [file pone.0028213.s019.jpg]

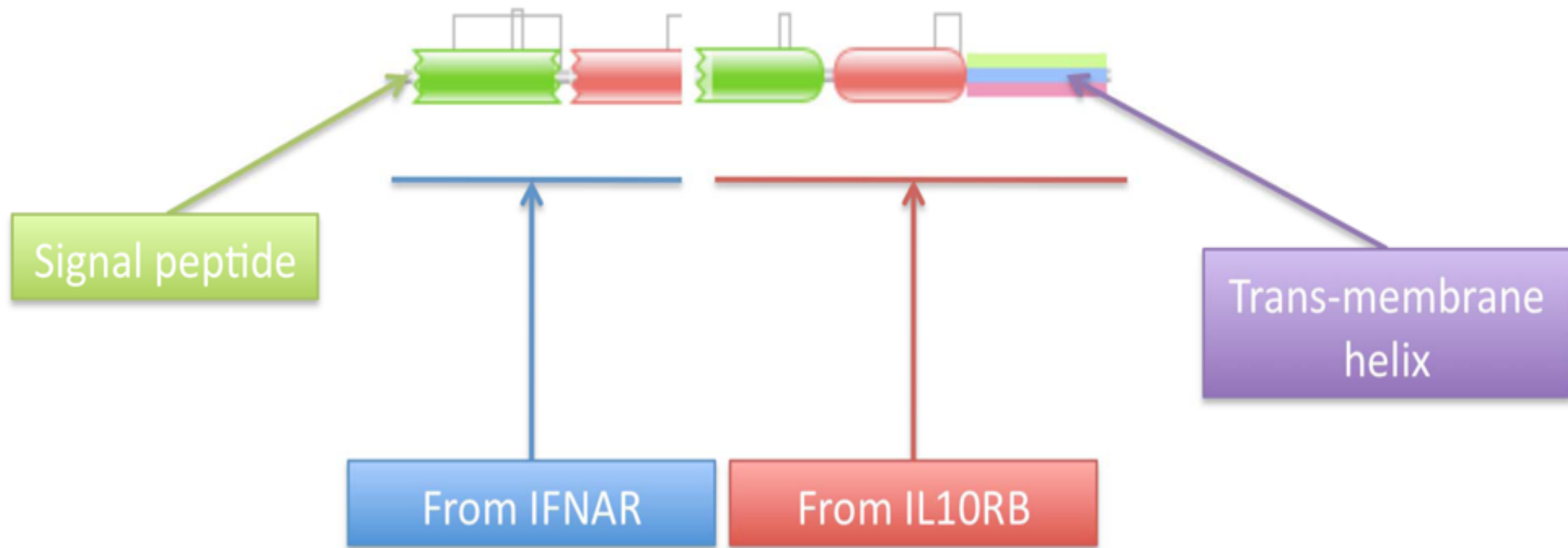

Supplement: Figure S19 — Domain organization for chimera OTTHUMP00000221101. Chimera OTTHUMP00000221101 results from the fusion of two receptors involved in immune response, Interferon-alpha/beta receptor 2 (IFNAR, N-terminal section) and Interleukin-10 receptor subunit beta (IL10RB, C-terminal section). The resulting protein will have an extra-cellular domain that is double the size of the usual extra-cellular receptor domain and that is composed of a repeat of paired tissue factor (green) and alpha/beta interferon receptor (red) domains. The chimeric protein also conserves a signal peptide signal and a single trams-membrane helix. A similar domain configuration is recorded in Uniprot for the chicken interferon receptor (Q5XPI1_CHICK). (PDF) [file pone.0028213.s020.pdf]

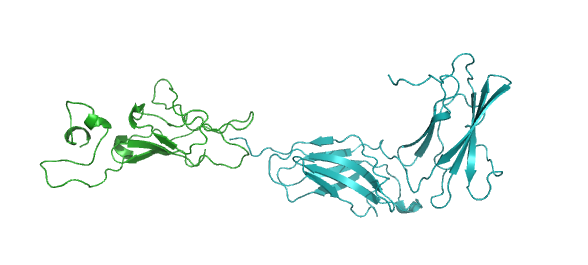

Supplement: Figure S20 — Model of possible structure of fused fragments for chimera OTTHUMP00000221101. Models for the N- and C-terminal sections have been obtained respectively from structures 2hym and 3g9v by comparative modeling (Modeller, http://salilab.org/modeller). Linker region (shown as a gap in the structure) is located in flexible regions for both templates. Domain folds could then be maintained independently. (PNG) [file pone.0028213.s021.png]

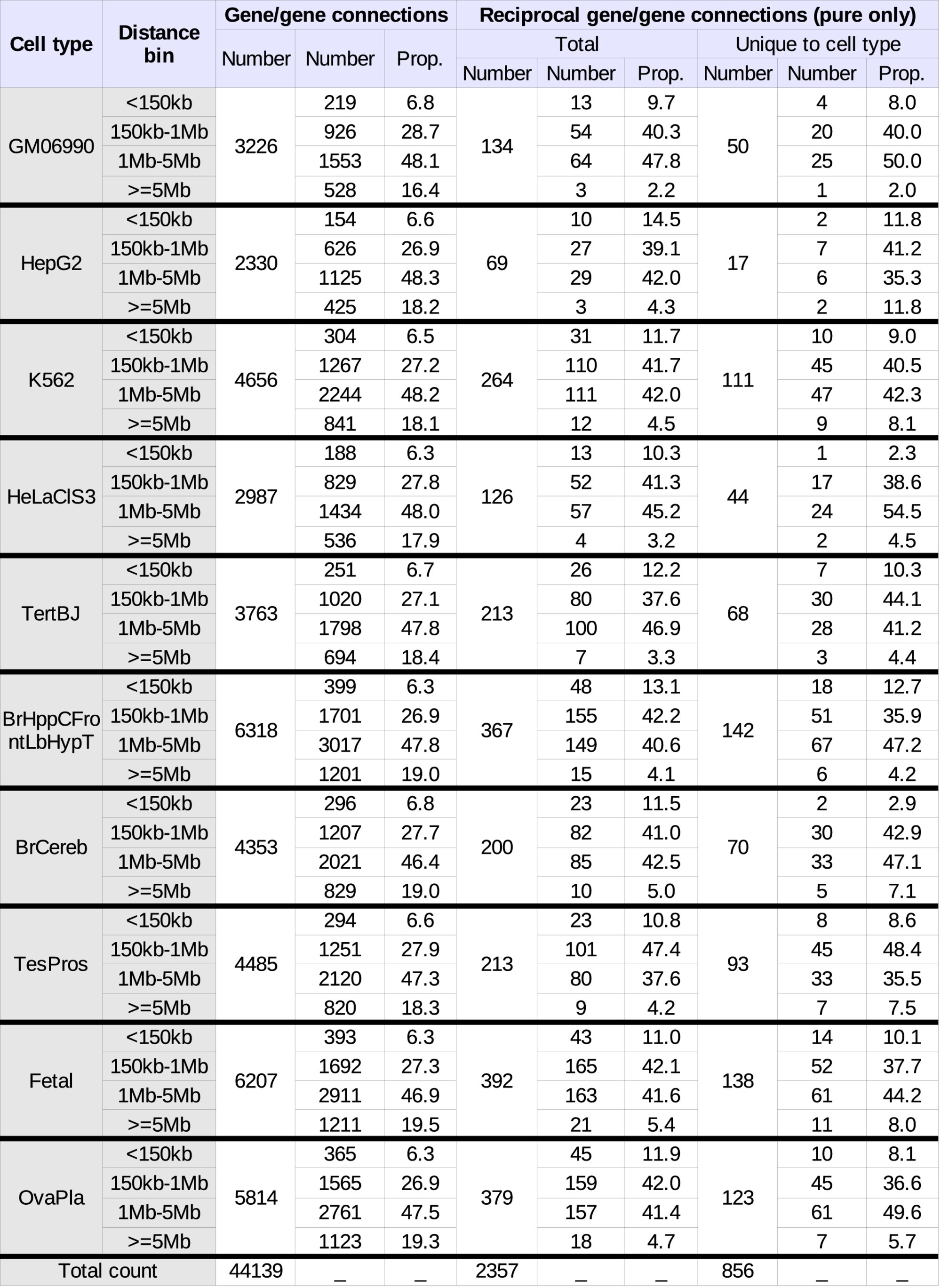

Supplement: Table S1 — Number of gene to gene and number of reciprocal gene to gene connections by distance. This table is similar to Figure 5B and provides numbers of gene to gene and of reciprocal gene to gene connections detected in each cell type, split by distance bins: - <150 kb -150 kb – 1 Mb -1 Mb – 5 Mb ->5 Mb. This table shows that (1) the number of connections is similar in cell lines and tissues, (2) the distribution of connections in distance classes changes if we consider all or only reciprocal connections, (3) between one third and half of the reciprocal connections are cell-type specific and (4) all the figures are quite high meaning that chimeras are far from being exceptional. (JPG) [file pone.0028213.s022.jpg]

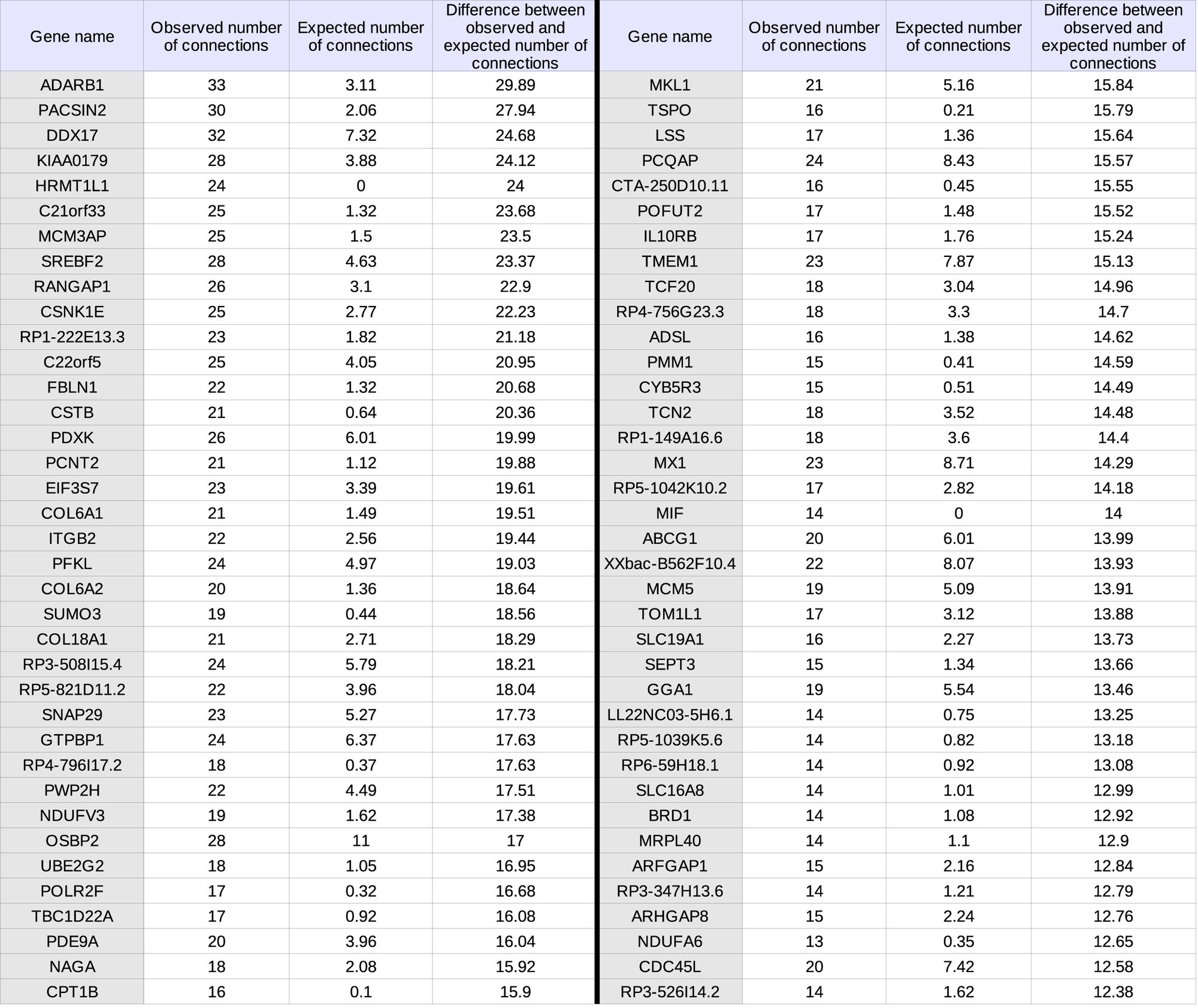

Supplement: Table S3 — Names and characteristics of the hubs. For each of the 74 hubs the table provides: - the number of observed connections, - the number of expected connections, - the difference between the two, which could be seen as their connectivity score. (JPG) [file pone.0028213.s024.jpg]

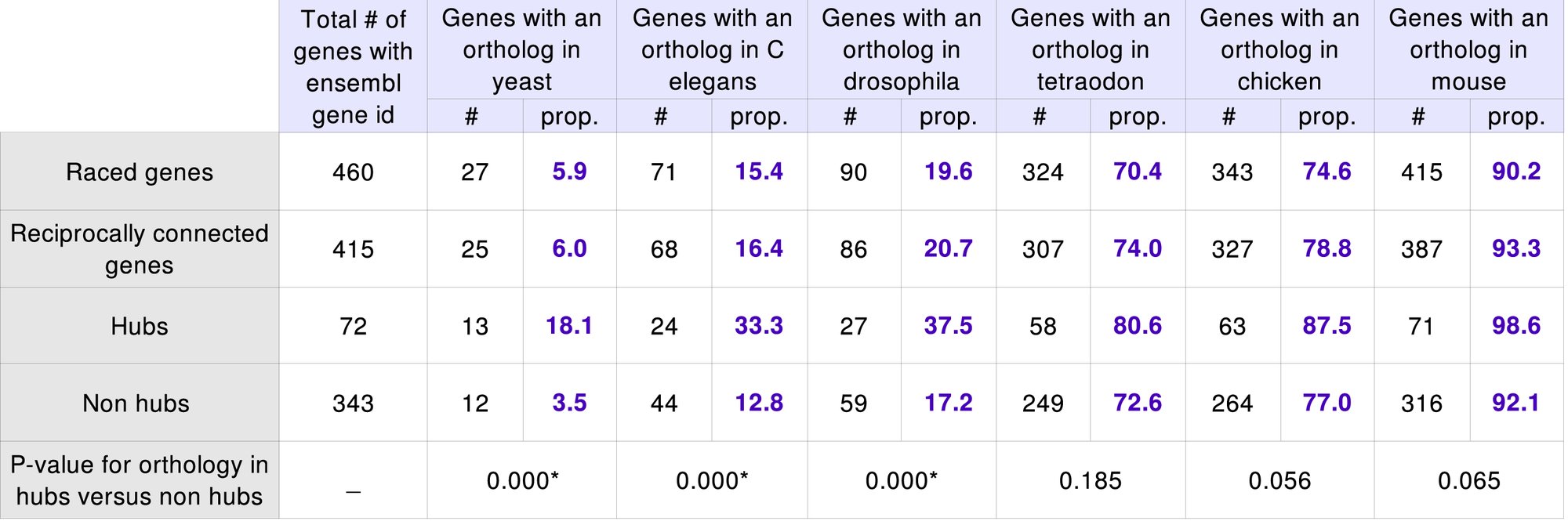

Supplement: Table S4 — Hubs have higher phylogenetic depth than non hubs. We consider four different gene sets (see Figure S14 for a description): - RACE interrogated (“raced”) genes - reciprocally connected genes – hubs - non hubs and for each of them, we provide the number of genes with an Ensembl gene ID, and the number and the proportion of this total, that has an ortholog in the 6 following species, as found using biomart on ensembl51: • Yeast • C. elegans • Drosophila • Tetraodon • Chicken • Mouse. For each of these species we then provide the Fisher exact test p-value obtained while testing the following hypothesis: “Being a hub is independent on having an ortholog in a given species”. A star above this number on the table means the p-value is significant (less than 0.01). Note that both the proportions of genes with an ortholog in each of the 6 species for RACE interrogated (“raced”) genes, hubs and non hubs, and the significance of the Fisher tests mentioned here are provided on Figure 9B. (JPG) [file pone.0028213.s025.jpg]

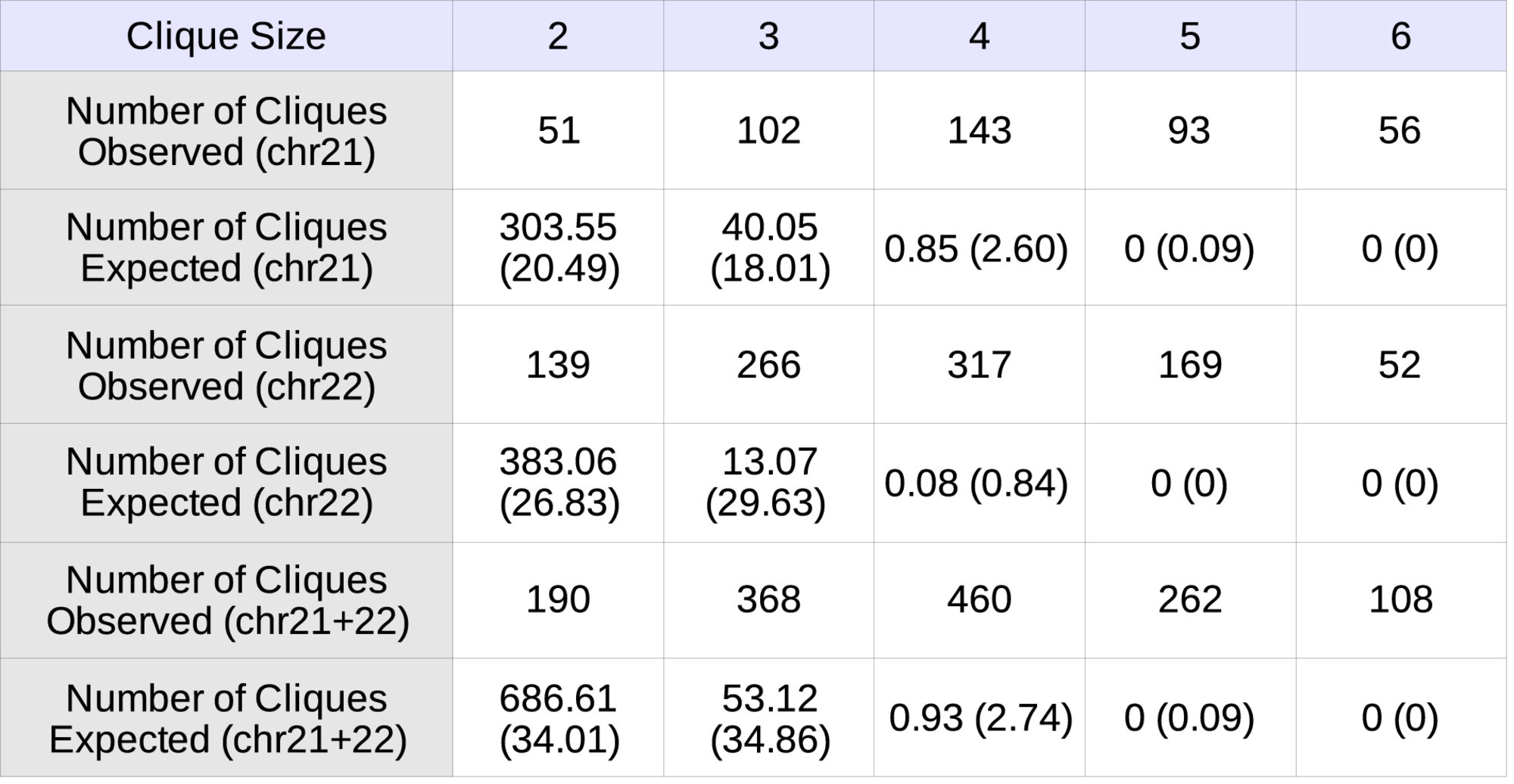

Supplement: Table S5 — Over-representation of cliques in chromosomes 21 and 22. For each chromosome, we report the number of cliques observed, as well as the mean and the standard deviation of the number of cliques expected. (JPG) [file pone.0028213.s026.jpg]

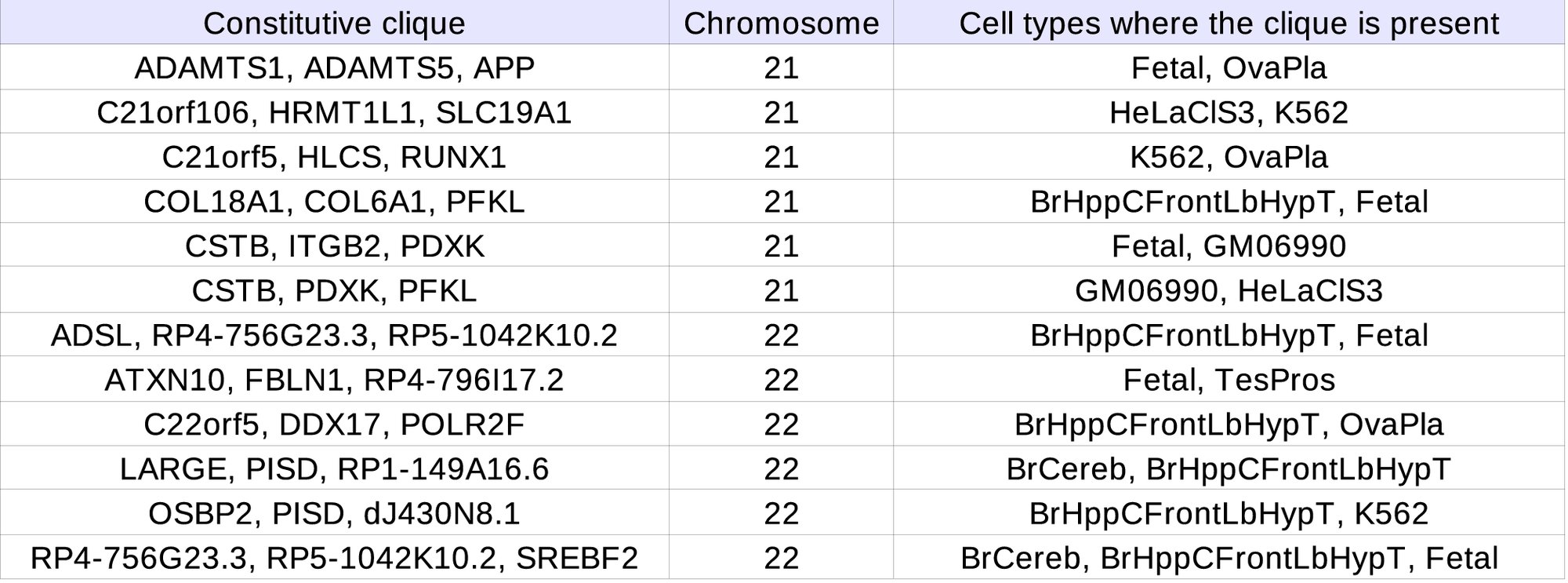

Supplement: Table S6 — Constitutive cliques. For each constitutive clique (the maximum size is 3), we provide: • the names of the genes involved in the clique, • the chromosome where the clique is observed, • the list of cell types in which the clique is observed. (JPG) [file pone.0028213.s027.jpg]

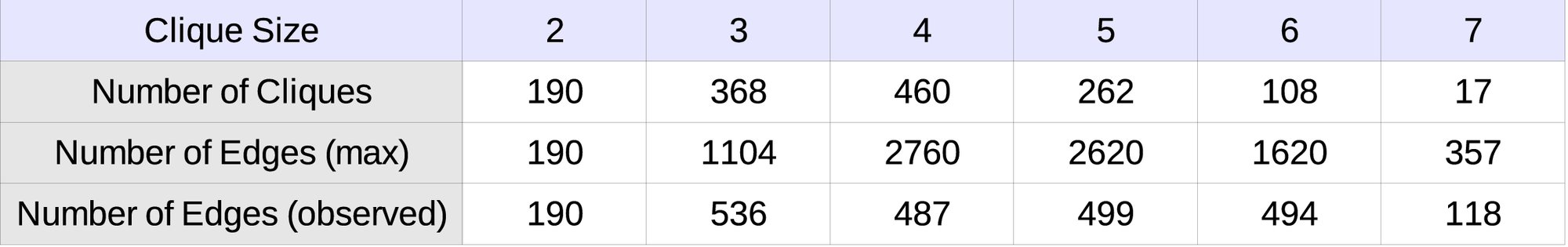

Supplement: Table S7 — Overlap of maximal cliques. For each clique size, we report: - the number of cliques observed, - the number of corresponding edges if cliques were not overlapping, - the number of observed edges. (JPG) [file pone.0028213.s028.jpg]
